# Comparison of prokaryotes between Mount Everest and the Mariana Trench

**DOI:** 10.1186/s40168-022-01403-y

**Published:** 2022-12-07

**Authors:** Yongqin Liu, Zhihao Zhang, Mukan Ji, Aoran Hu, Jing Wang, Hongmei Jing, Keshao Liu, Xiang Xiao, Weishu Zhao

**Affiliations:** 1 Center for Pan-third Pole Environment, Lanzhou University, Lanzhou, China; 2 State Key Laboratory of Tibetan Plateau Earth System, Resources and Environment (TPESRE), Institute of Tibetan Plateau Research, Chinese Academy of Sciences, Beijing, China; 3 State Key Laboratory of Microbial Metabolism, School of Life Sciences and Biotechnology, Shanghai Jiao Tong University, Shanghai, 200240 China; 4 International Center for Deep Life Investigation (IC-DLI), Shanghai Jiao Tong University, Shanghai, 200240 China; 5 School of Oceanography, Shanghai Jiao Tong University, Shanghai, 200240 China; 6 SJTU Yazhou Bay Institute of Deepsea Sci-Tech, Yongyou Industrial Park, Sanya, 572024 China; 7 Institute of Deep-Sea Science and Engineering, Chinese Academy of Sciences, Sanya, 572000 China; 8 Southern Marine Science and Engineering Guangdong Laboratory (Zhuhai), Zhuhai, Guangdong China

**Keywords:** Prokaryotic community, Mount Everest, Mariana Trench, Biodiversity, Metabolic capability, Shotgun metagenomics

## Abstract

**Background:**

Mount Everest and the Mariana Trench represent the highest and deepest places on Earth, respectively. They are geographically separated, with distinct extreme environmental parameters that provide unique habitats for prokaryotes. Comparison of prokaryotes between Mount Everest and the Mariana Trench will provide a unique perspective to understanding the composition and distribution of environmental microbiomes on Earth.

**Results:**

Here, we compared prokaryotic communities between Mount Everest and the Mariana Trench based on shotgun metagenomic analysis. Analyzing 25 metagenomes and 1176 metagenome-assembled genomes showed distinct taxonomic compositions between Mount Everest and the Mariana Trench, with little taxa overlap, and significant differences in genome size, GC content, and predicted optimal growth temperature. However, community metabolic capabilities exhibited striking commonality, with > 90% of metabolic modules overlapping among samples of Mount Everest and the Mariana Trench, with the only exception for CO_2_ fixations (photoautotrophy in Mount Everest but chemoautotrophy in the Mariana Trench). Most metabolic pathways were common but performed by distinct taxa in the two extreme habitats, even including some specialized metabolic pathways, such as the versatile degradation of various refractory organic matters, heavy metal metabolism (e.g., As and Se), stress resistance, and antioxidation. The metabolic commonality indicated the overall consistent roles of prokaryotes in elemental cycling and common adaptation strategies to overcome the distinct stress conditions despite the intuitively huge differences in Mount Everest and the Mariana Trench.

**Conclusion:**

Our results, the first comparison between prokaryotes in the highest and the deepest habitats on Earth, may highlight the principles of prokaryotic diversity: although taxa are habitat-specific, primary metabolic functions could be always conserved.

Video abstract.

**Supplementary Information:**

The online version contains supplementary material available at 10.1186/s40168-022-01403-y.

## Background

Extreme environments are widespread on Earth, such as hot springs, hydrothermal vents, deserts, glaciers, deep sea, and hadal trenches, which have a wide variety of harsh conditions and provide unique and diverse ecological niches for life on Earth [1]. Prokaryotes are the primary life forms in extreme environments, teaching us about life’s limits [2–4]. Prokaryotes are the most widespread form of life, encompassing incredibly diverse taxa and encoding the vast majority of biological functions, playing crucial roles in changing, shaping, and sustaining habitability on Earth [5–7]. The extraordinary diversity of prokaryotes is believed to be a product of ~ 3.8 billion years of evolution, 2 billion years longer than that of eukaryotic organisms [8]. It is generally accepted that prokaryotic species are closely related to the environmental conditions of their habitats [9] and most species-level genes (> 95% nucleotide identity) are specific to a single habitat [6]. However, the functional redundancy that multiple prokaryotic taxa encode the same biological function [10] and cross-stress adaptation behaviors for coping with distinct environmental conditions [3] are also reported in many studies. These two seemingly contradictory results bring us a new hypothesis: the prokaryotic taxa tend to adapt to the environment; in contrast, most metabolic pathways (including some adaptation mechanisms) are still conserved, even in two completely different extreme environments. To test this hypothesis, here we chose the highest habitats and the deepest habitats for prokaryotes on Earth, Mount Everest (ME) and the Mariana Trench (MT), respectively, for comparing the taxonomy and metabolic capability of the prokaryotic community and gaining new insights into the prokaryotic biodiversity in two isolated extreme environments.

Mount Everest (ME) is the highest area on Earth, with an 8.8-km elevation summit, and is covered by snow and ice [11]. Observations in situ reveal that > 6 km above sea level (a.s.l) on ME are environments with intense ultraviolet (UV) radiation, as well as the low but dramatically changeable temperatures that vary with seasons and day/night cycles [12]. The automatic meteorological station at 6.5 km a.s.l. recorded the highest net radiation, 392.52 W/m^2^, and temperature ranged from −19.3 to 9.4 °C, with an average of −5.5 °C from May to July 2021 (unpublished data). In addition, the high elevation of ME also causes lower pressure than the standard atmospheric pressure (0.0033 MPa at the summit of Mount Everest) [13] and lower nutrient concentration than normal environments (e.g., in soils) [11, 14, 15]. These extreme environmental parameters challenge all life forms living in ME. Bacterial abundances in surface snow, moraine lakes, glacier streams, and meltwaters of ME above 6 km were reported with a low concentration of cells, approximately 10^4^–10^5^ cells/mL [11, 16–18]. Seasonal variation has been observed for bacterial community structure in snow samples [19]. Both culture and 16S rRNA gene amplicon-based studies reveal that the bacterial community in ME is relatively simple, commonly predominated by Proteobacteria, Actinobacteria, Firmicutes, Cyanobacteria, and Deinococcus-Thermus [11, 14, 16, 17, 20]. Thus far, the recent published study on genome and gene catalog of glacier microbiomes is the only one publication that included the prokaryotic metagenome at 4.6–6.7 km on the Tibetan Plateau, while the metabolic potentials and adaptative mechanisms of prokaryotes in ME remain unclear which needs further investigation [4].

In contrast, the Mariana Trench (MT) is the deepest region on Earth. The 11-km water depth below sea level (b.s.l) of the Challenger Deep, the deepest area in MT, is far beyond areas reached by sunlight. For this reason, the MT is characterized by a stable and near-freezing temperature (~ 2°C), total darkness, and poor nutrient availability, but under an ultrahigh hydrostatic pressure (HHP) up to ~ 115 MPa [21, 22]. Cellular life living in MT usually needs unique mechanisms to cope with the HHP, such as intercellular accumulation of compatible solutes (e.g., TMAO) in both macro- and microorganisms [23–25]. The biomass density at the bottom of MT is significantly lower than that at the marine surface, 10^3^–10^4^ cells/mL in near-bottom water and 10^5^–10^7^ cells/mL in sediments [26, 27]. Microbiomes in MT samples have been reported to be with high novelties, especially in bottom-axis sediment samples [28]. In addition, the V-shaped topography of MT enables the collection of particulate organic matters (POMs) and heavy metals, e.g., arsenic (As) and selenium (Se), from the overlying water column, abyssal seafloor, and Earth’s upper crust [28, 29]. These accumulation effects result in a higher content of organic matters (OMs) and more intense microbial degradation activities at the bottom-axis than in adjacent slope sites in MT [30], as well as a higher concentration (> 2-fold) of As and Se in MT sediment samples than those at non-hadal sites [28]. The only two previous metagenomic studies of the sediments in the Challenger Deep of MT suggested the essential roles of predominant heterotrophs in recycling macromolecules and utilizing various carbon sources (e.g., peptides, carbohydrates, hydrocarbons, and aromatic compounds) [21, 28].

**Fig. 1:**
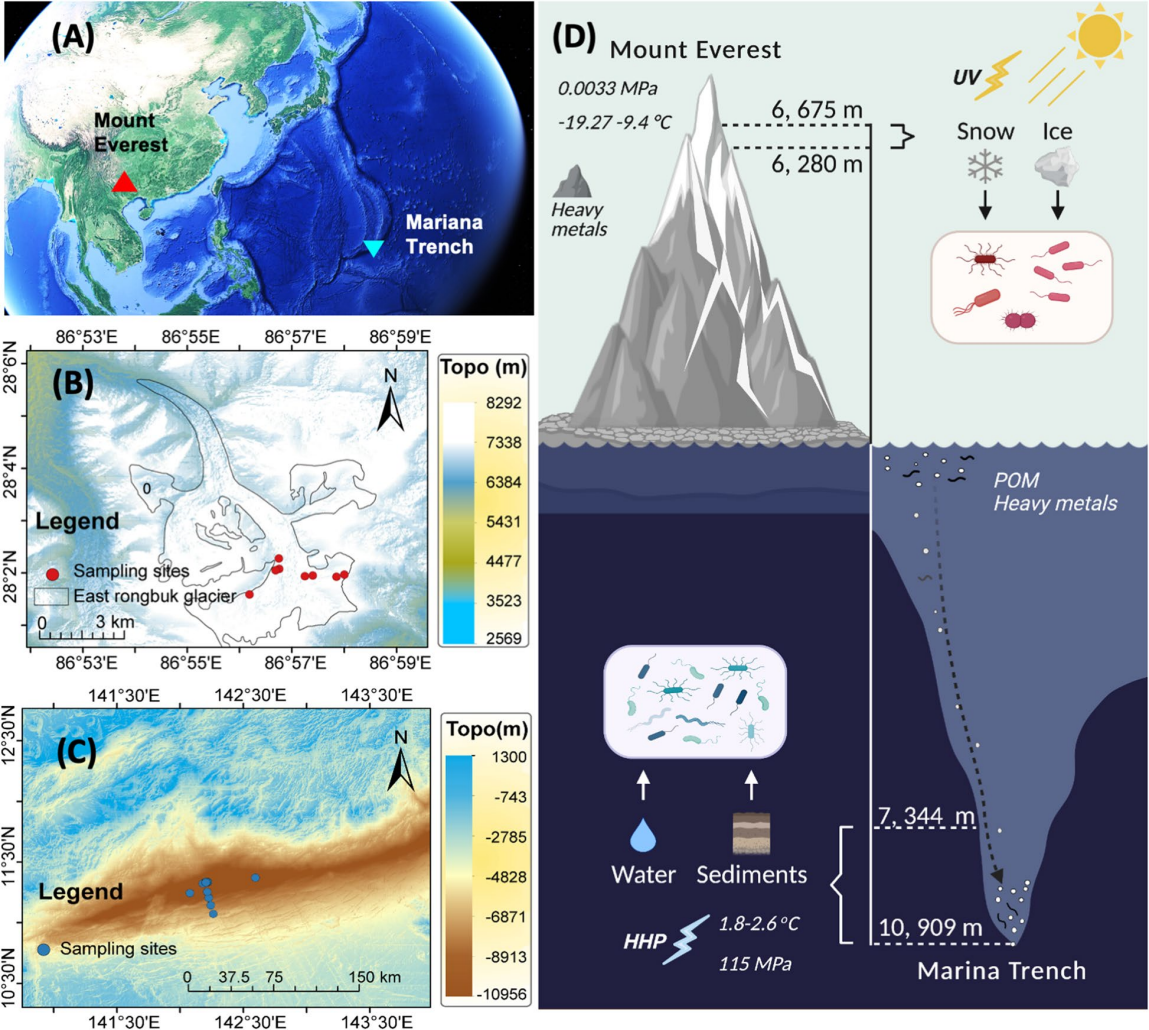
Sampling sites in Mount Everest (ME) and the Mariana Trench (MT). **A** Location of the ME and MT on the map of Google Earth. Detailed sampling sites in the ME (**B**) and the MT (**C**). **D** Schematic diagram of the samples used in this study

In this study, we compare the taxonomy and metabolic capability of prokaryotes between ME and MT by using nine samples of ice/snow at 6–7 km a.s.l. from the East Rongbuk Glacier of ME and 16 samples of water/sediment with depths of 7–11 km b.s.l from the Challenger Deep in MT (Fig. 1). Metagenomic analysis between ME and MT revealed a surprisingly opposite scenario in terms of taxonomy and metabolic capability. The taxonomic diversity between ME and MT was highly variable, with significant differences in community composition, genome size, GC content, and predicted optimal growth temperature between these two habitats. In contrast, the diversity of metabolic capabilities was much more consistent between ME and MT. Most metabolic modules (> 90%) were shared across ME and MT. Near-complete metagenome-assembled genomes (MAG)-based analysis suggested the functional redundancy that distinct prokaryotic taxa can carry the same metabolic functions in ME and MT. These results provide extreme examples for prokaryotic comparison from the habitats with the greatest height differences on Earth, providing a new perspective to understanding prokaryotic biodiversity in terms of taxonomy and metabolism and their specificity to different extreme habitats.

## Methods

### Sampling and preprocessing

Since the environmental conditions of ME and MT are so different, it is almost impossible to collect exactly the same type of sample to compare. Here we selected the most representative samples of ME or MT, including 9 samples (snow and ice) from ME and 16 samples (water and sediment) from MT (Fig. 1, Table 1). Among them, snow and ice represent the typical habitats that harbored diverse prokaryotes on glacier surface of Mount Everest [31]; similarly, water and sediment are the main habitats of prokaryotes in trenches [32]. For ME samples, 1 snow sample and 8 ice samples were collected at 8 sites on northern slope, the East Rongbuk Glacier, of Mount Everest with different elevations ranging from 6280 to 6675 m. The snow sample was collected at ~5-cm depth by a sterile steel spoon, and eight ice samples were collected with pre-clean ice axe, then surface 2 mm of the ice samples was scraped with a sterile scalpel for decontamination. Detailed information of sample sites is shown in Table 1. About 2L of each sample was placed in WhirlPak bags (WhirlPak®, Nasco, USA), and all samples were placed in icebox during transport from sampling site to the laboratory. Snow and ice samples were melted at 10 °C in the lab and filtered onto a 0.22-μm polycarbonate filter membrane (47-mm diameter; Millipore) for further DNA extraction.

**Table 1:** Sampling sites in this study

Location	Sample ID	Longitude (°E)	Latitude (°N)	Above sea water (m)	Sample type	Sampling method	T (°C)	Salinity (PSU)
**Mount Everest**	1806ZF6500I1	86.9364	28.0265	6500	Ice	Pre-clean ice axe	NA	NA
	1905ZF6280I1	86.9458	28.0380	6280	Ice	Pre-clean ice axe	NA	NA
	1905ZF6320I2	86.9459	28.0347	6320	Ice	Pre-clean ice axe	NA	NA
	1905ZF6360I3	86.9541	28.0323	6360	Ice	Pre-clean ice axe	NA	NA
	1905ZF6400I1	86.9566	28.0325	6400	Ice	Pre-clean ice axe	NA	NA
	1905ZF6400I2	86.9566	28.0325	6400	Ice	Pre-clean ice axe	NA	NA
	1905ZF6500I1	86.9642	28.0321	6500	Ice	Pre-clean ice axe	NA	NA
	1905ZF6675I1	86.9667	28.0328	6675	Ice	Pre-clean ice axe	NA	NA
	1905ZF6360S	86.9448	28.0342	6360	Snow	Sterile steel spoon	NA	NA
**Mariana Trench**	TY.041	142.2418	11.1440	− 8563	Water	Niskin Bottle	2.02	34.68
	TY.044	142.2624	11.0742	− 7344	Water	Niskin Bottle	1.80	34.70
	WQ.018	142.0764	11.2450	− 9659	Water	Niskin Bottle	2.22	34.70
	WQ.021	142.2172	11.3369	− 10,901	Water	Niskin Bottle	2.47	34.70
	WQ.022	142.1988	11.3334	− 10,875	Water	Niskin Bottle	2.46	34.70
	WQ.024	142.2114	11.3360	− 10,877	Water	Niskin Bottle	2.46	34.69
	YW.019	142.2068	11.3355	− 10,884	Water	Niskin Bottle	2.47	34.70
	YW.020	142.2151	11.3346	− 10,898	Water	Niskin Bottle	2.47	34.70
	YW.021	142.1791	11.3250	− 10,894	Water	Niskin Bottle	2.47	34.70
	YW.023	142.5955	11.3724	− 10,905	Water	Niskin Bottle	2.47	34.70
	GT04.1	142.2026	11.3316	− 10,925	Sediments	GravityCore	NA	NA
	GT04.2	142.2026	11.3316	− 10,925	Sediments	GravityCore	NA	NA
	GT04.3	142.2026	11.3316	− 10,925	Sediments	GravityCore	NA	NA
	GT04.4	142.2026	11.3316	− 10,925	Sediments	GravityCore	NA	NA
	TY.038	142.2031	11.3311	− 10,893	Sediments	BoxCore	NA	NA
	TY.042	142.2129	11.2547	− 10,109	Sediments	BoxCore	NA	NA

For MT samples, 10 water samples and six sediment samples were collected at 13 sites near the Challenger Deep in the Mariana Trench aboard the R/V “Tansuo-01,” with varying water depth ranging from 7344 m to ~ 11 km b.s.l, under hydrostatic pressure from 75 to 115 MPa. Among these 16 samples, 13 were collected from the bottom of the Challenger Deep with nearly the deepest water depth (> 10 km) on Earth. The temperature of these 16 MT sampling sites ranged from 1.8 to 2.6 °C and varies with the water depth (Table 1). All seawater samples were collected using Niskin bottles from the Mariana Trench during TS-09 cruise in September and October 2018. Samples from station TY044 and TY041 were filtered through 0.22-μm polycarbonate membranes (142 mm, Millipore) directly, and other 8 samples were filtered serially through 3-μm and 0.22-μm polycarbonate membranes aboard. After adding RNAlater^TM^ Stabilization Solution (Thermo Scientific, Wilmington, DE, USA), all filters were frozen at −80°C immediately until further analysis. In situ hydrographical parameters (e.g., salinity, temperature, and depth) were measured by a conductivity-temperature-depth (CTD) rosette system (Sea-Bird Electronics). Sediment samples were collected by box-core at stations TY038 and TY042 during TS-09 cruise, and by gravity-core at station GT04 during the cruise TS01-14. The box-core samples were mixed and frozen at −80 °C immediately until further analysis, while the gravity-core samples were cut at each 2–4 cm and then frozen at −80°C until further analysis. Detailed information of sampling is shown in Table 1.

### DNA extraction and sequencing

Metagenomic DNA of snow, ice, and seawater samples was extracted from 0.22-μm filters with a PowerSoil DNA Isolation Kit (Qiagen, Germantown, USA) following its protocol. The concentration of DNA was quantified by Qubit DNA Assay Kit with a Qubit 2.0 fluorometer (Invitrogen, Carlsbad, CA, USA), and the quality was checked by gel electrophoresis. The sequencing library was prepared using the KAPA hyper Prep Kit (Roche, Shanghai, China). The shotgun sequencing was performed on Illumina NovaSeq 6000 Platform PE150 (Illumina, San Diego, CA, USA) with 150 bp paired-end reads. Genomic DNA of sediment samples from TY038 and TY042 stations was extracted from ~ 5 g by modified SDS method as descript in a previous study [33]. DNA of sediment samples from GT04 station was extracted from ~ 0.3 g of sediment per sample using PowerSoil® DNA Isolation Kit (QIAGEN, Germany). The sequence library was built in Beijing Genome Institution (BGI, Shenzhen, China) and sequenced on the BGI MGISEQ-2000 platform, which has been reported can obtain comparable results as Illumina platform [34].

### Quality control and reads analysis

Metagenomic raw reads are adapter-trimmed using TrimGalore (version 0.6.6) and filtered using sickle (version 1.33) with parameters (--length-threshold 90 -t sanger -g) [35]. Filtered read quality is estimated and reported by fastQC (v0.11.4) and multiQC (version 1.9) [36]. Small-subunit rRNA (SSU rRNA) genes are assembled from clean reads using phyloFlash (v3.4) with parameters (-almosteverything), depending on SILVA database version 138.1 [37, 38]. To assess the alpha diversity, Shannon and richness indexes were evaluated based on counts of SSU sequences extracted from all clean reads of the metagenomes of ME and MT samples, from domain to species levels. Metagenomic contigs are assembled from clean reads using megahit (v1.2.9) with default parameters and --min-contig-len 500 [39]. Clean reads were then mapped to contigs using bbmap.sh (last modified on February 13, 2020) with parameters (nodisk k=13 minid=0.95 keepnames=t minaveragequality=5 trimreaddescriptions=t pairlen=350 rescuedist=650) [40]. Genes are predicted by prodigal (V2.6.3) with parameter (-p meta) [41, 42]. Genes not shorter than 99 bp (33 aa) are then annotated by KofamKOALA (version 1.3.0, database build 2021-01-04) and eggNOG (version 2.1.3, database 5.0.2) to assign KEGG KO to genes [43]. We also download a description of a pathway to module (ko00002), pathways, and module for further analysis. After gene annotation, gene coverage is calculated using featureCounts (v2.0.1) [44].

### Binning and MAG-level analysis

Metagenomic binning and refinement were performed on contigs using a combination of metabat2 (version 2:2.15), maxbin2 (version 2.2.7), concoct (version 1.1.0), and DAS Tool (version 1.1.2) (Additional file 1: Fig. S1) [45–47]. Megahit was run with nine combinations of parameters (--maxP with 60, 75, 90, and –minS with 60, 75, 90). Metabat2 was used with two combinations of parameters (-markerset with 107 and 40). Binnig results of the total 12 parallel methods are then refined using DASTool with parameters (--search_engine diamond –score_threshold 0). Next, binning results from all samples are collected and representative MAGs are choired using dRep (v3.0.0) with parameters (-comp 50 -con 10 -pa 0.9 -sa 0.95) [48]. Finally, 1176 MAGs with completeness ≥ 50 and contaminant ≤ 10 including 411 MAGs with completeness ≥ 90 and contaminant ≤ 5 are clustered into 648 clusters [49]. We used GTDB-Tk (v1.6.0) to taxonomically classify representative MAGs of all clusters with the GTDB release 202 [50, 51]. Next, we use prodigal without parameter (-p meta) and annotate gene function as descript above. Reads are mapped to genes of all MAG genomes by minimap (version 2.1) equivalent to the method used by coverM (version 0.6.1) [52].

### Statistical analysis

mOTUs are first filtered and only keep prokaryotes, and analyzed at phylum to species level, any taxon that exists in fewer than two samples or maps lower than ten reads are filtered. Mapped reads of genes are grouped by KO or KEGG module for each sample [6]. Distances between samples were calculated by *vegdist* in R package *vegan*, when calculating the binary Jaccard distance, we use binary format of reads table, and the Bray-Curtis distance between samples is calculated using total reads [53]. A method equivalent to TPM was used to normalize gene abundance data for metagenomic comparation. Briefly, reads mapped to genes were normalized by gene length and total mapped number following the formula below:

$$\textrm{TP}{\textrm{M}}_{\textrm{k}}=\frac{{\textrm{r}}_{\textrm{k}}/{\textrm{l}}_{\textrm{k}}}{\sum \left({\textrm{r}}_{\textrm{i}}/{\textrm{l}}_{\textrm{i}}\right)}\times {10}^6$$

in which *r*_*i*_ and *l*_*i*_ refer to the reads mapped to gene *i* and the length of gene *i*. The significant difference between different locations was calculated using *adonis* method in R package *Vegan*. Significance of the MAG-based comparisons of predicted G+C content, genome size, optimal growth temperature, and minimum generation times between different locations are using *Wilcox* test in *ggsignif* package in R [54]. Co-occurrence networks of genes were constructed based on their presence/absence across ME or MT-specific MAGs based on binary Jaccard distance. Edges in the network represent a co-occurrence level of > 0.3. A full record of all statistical analysis is included as Additional file 4 and was created using the *knitr* package in R [55].

### Phylogenomic analysis of MAGs

A phylogenetic tree of reconstructed representative genomes above middle-quality was constructed using the protein sequences of 40 universal gene markers. Approximate maximum-likelihood trees were generated using FastTree (version 2.1.10) and bootstrap values of both phylogenomic trees were evaluated based on 1000 replicates. Other 100 reference genomes from GTDB database were chosen according to fastANI similarity. Genes of each genome are predicted by prodigal in “single” mode, and fetchMGs (v1.2) was used to extract 40 conserved single-copy genes of prokaryotes [56]. Genomes with less than 20 marker genes extracted were filtered. Marker genes are aligned by MAFFT (v7.487) with parameters (--maxiterate 1000 --localpair) and concatenated to trim by trimAl (v1.4.rev15) with the automated1 mode. FastTree (version 2.1.10) was used to construct the tree with parameter (-gamma). All trees were further polished and visualized using the interactive tree of life (iTOL) v5 [57].

### MAG-based physiological predictions

Estimation of optimal growth temperature was performed for medium-high-quality MAGs using Growthpred by searching highly expressed gene (ribosomal protein gene) in genomes [58]. We discarded MAGs that belong to the Archaea domain and the number of highly expressed gene blow 10, then estimate minimum generation times using growthpred-v1.08.py with parameters (-r -t -c 0) [59].

## Results

### Description of prokaryotes from ME and MT

Total of the 9 ME and 16 MT samples yielded 4,846,075,106 clean raw read pairs (814,407,384 for ME, 4,031,667,722 for MT) after being trimmed and filtered, composing 98% of the raw reads (see the “Methods” section). Since eukaryotic reads account for only 3.45% in average of 25 metagenomes from ME and MT, we only focus on the prokaryotes in this study. Assembly of the above 25 metagenomes generated 16,958,325 scaffolds (average length 1238.34 bp). Binning of these contigs resulted in 1176 draft genomes with qualities above the middle-quality threshold (≥ 50% completeness and ≤ 10% contamination), among which 411 were above the high-quality threshold (≥ 90% completeness and ≤ 5% contamination) according to a widely accepted standard of MAGs [49]. Quality-controlled reads from all metagenomes had relatively high mapping rates (51.5%) to all MAGs above the middle-quality threshold, suggesting an acceptable representation and quality for the use of these MAGs to reflect the prokaryotic community in further analyses.

### Distinct taxonomic composition of prokaryotic communities between ME and MT

A total of 1,841,892 reads were mapped to 4084 molecular operational taxonomic unit (mOTU) sequences at the species level or higher based on the MiTAG method using phyloFlash (v3.4) (Methods, Additional file 1: Fig. S1A, Additional file 2: Table S1). A high proportion of microbial dark matter was observed in samples from these two extreme environments. For example, ~50% of family-level and ~80% of genus-level mOTUs were unclassified according to the SILVA 138.1 database [38]. A similar conclusion could be obtained from MAGs, in which 34.5% of high-quality MAGs were unclassified against the Genome Taxonomy database (GTDB) database at the species level.

**Fig. 2:**
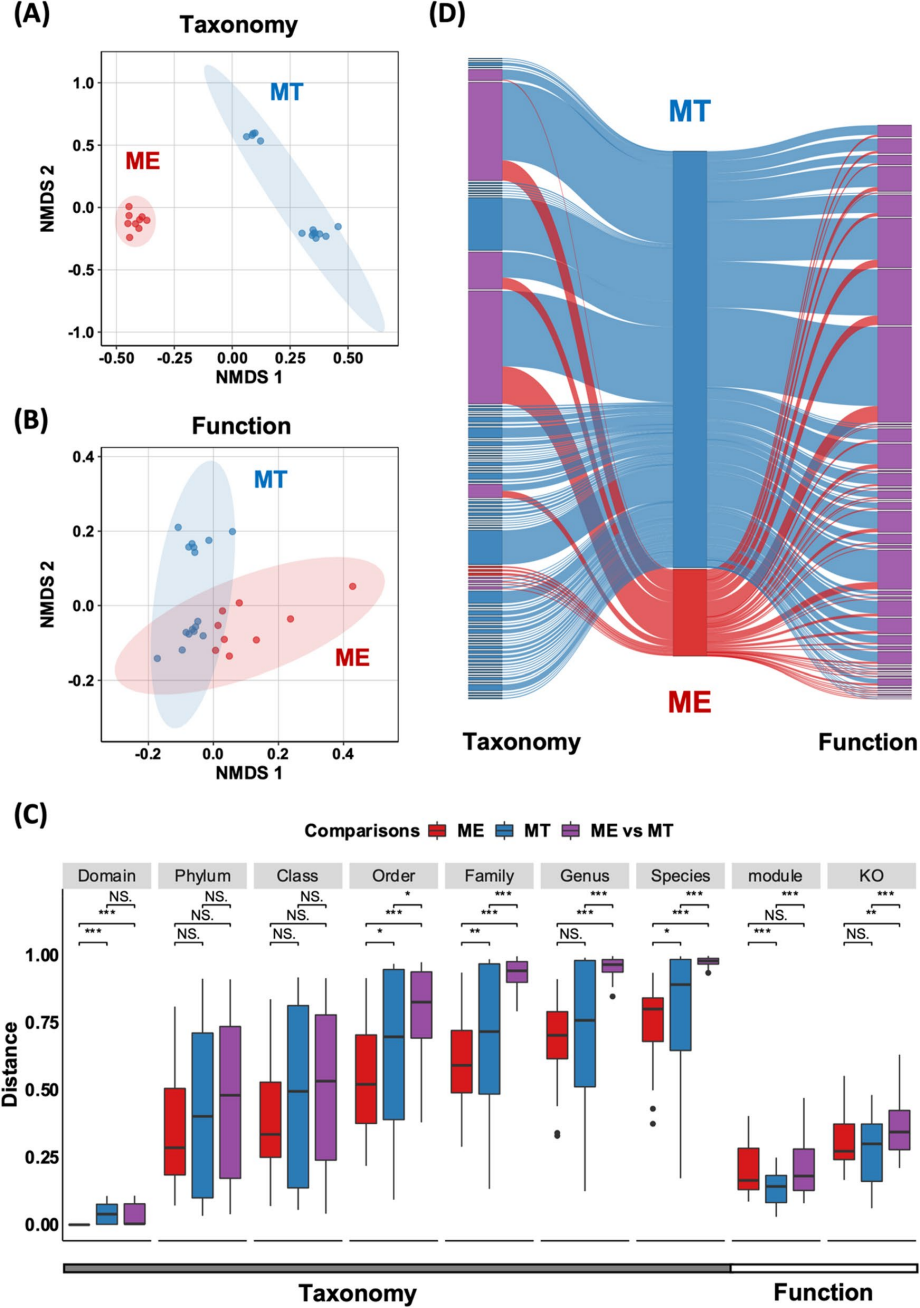
Taxonomic and functional diversity of microbiome in Mount Everest (ME) and the Mariana Trench (MT). **A** Taxonomic composition between ME and MT. Calculation was performed on species abundancy against the clean reads of metagenome. **B** Functional diversity between ME and MT. Calculation was performed on KO abundancy against the clean reads of metagenome. **C** Comparison of dissimilarities of function and taxonomy within ME, within MT and across two habitats. The dissimilarities of functions and taxonomy were reflected by the Bray-Curtis distance. Statistic significances were calculated by *Wilcox* test (*p-value < 0.05; **p-value < 0.001; ***p-value < 1e−5), while no significance was represented by “NS”. **D** Sankey network of taxonomy and function between ME and MT. Calculation was performed on 648 representative MAGs (above middle-quality, after removing redundancy). We used class level to reflect taxonomy and KEGG metabolic modules to reflect function. ME-specific taxa and functions were represented in red, and MT-specific were represented in blue, while common taxa and functions were represented in purple. More detailed information is shown in Additional file 1: Fig. S1 and Fig. S6

We independently calculated the alpha and beta diversity of prokaryotic metagenomes from ME and MT samples to reflect and compare the prokaryotic diversity and community dissimilarity (see the “Methods” section). The richness index of the prokaryotic community of ME was significantly lower than that in MT samples at the phylum level, while the opposite pattern was observed at the species level (*Welch’s t* test; *p* value < 0.05) (Additional file 1: Fig. S2). Beta diversity was assessed to compare the dissimilarity of prokaryotic communities between ME and MT. This dissimilarity between the two extreme environments increased from the domain to species level (Fig. 2C) and significantly differed at levels below class (*Adonis* test; *p* value < 0.05), regardless of the presence (binary Jaccard distance) or abundance (Bary-Cruit distance) of taxa (Fig. 2A, Additional file 1: Fig. S3). These comparisons of alpha and beta diversity reveal apparent differences in the taxonomic composition of prokaryotic communities between ME and MT. The relative abundance of taxa to class level among ME and MT samples calculated by mOTUs also shows the distinct community composition between ME and MT (Additional file 1: Fig. S1B).

### High environmental specificity in prokaryotic taxa

**Fig. 3:**
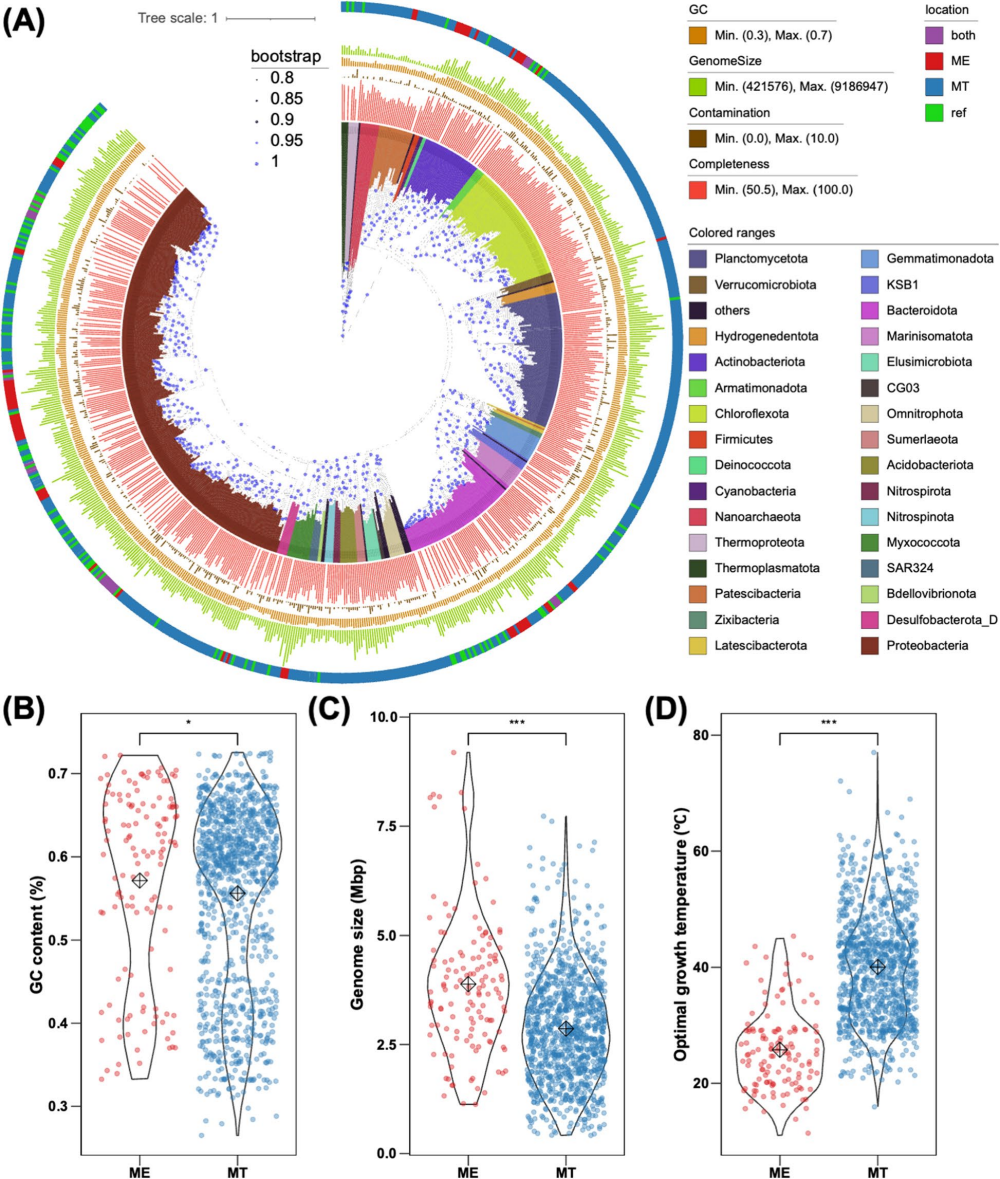
Phylogeny, taxonomy, and comparison genomic and predicted physiological features of MAGs between ME and MT. **A** Phylogenetic tree of MAGs from ME and MT in the species level. This phylogeny was generated using 648 genomes after dereplication clustered into species level (ANI ≥ 95). Bar plots are shown completeness (red) and contamination (brown) of MAGs. Colorstrip in outer ring represents the occurrence of MAGs between ME and MT in the genus level, such as red for ME, blue for MT, and purpose for present in both (shared genus labeled in outer line). Bootstraps are shown in the purple triangle and are based on 1000 replicated trees. Comparison of **B** genome size, **C** GC content, and **D** predicted optimal growth temperature of MAGs between ME and MT. Red dots and boxes represent the data from ME, while blue dots and boxes represent the data from MT. Significant difference between ME and MT is represented by “*” and “***” where the *p*-value is less than 0.05 and 1e−5 (*Wilcox* test), representatively

In addition to the analysis of clean reads, the MAG-based Sankey network also revealed the distinct prokaryotic taxa between ME and MT (see the “Methods” section; Fig. 2D). We further constructed a phylogenetic tree by using 648 representative MAGs (dereplicated at 95% average nucleotide identity (ANI)) and 100 reference genomes from the GTDB [50] (see the “Methods” section, Fig. 3A). Except for two unclassified MAGs, the remaining MAGs were distributed into 47 phyla. Among them, seven phyla were commonly identified at both ME and MT. Regardless of ME or MT, most MAGs belonged to the phylum Proteobacteria, followed by the phyla *Chloroflexota*, *Planctomycetota*, *Bacteroidota*, *Actinobacteriota*, *Marinisomatota*, and *Gemmatimonadota*. Specific phyla from ME samples were *Cyanobacteria* and *Deinococcota*. No archaea group was observed in metagenomes from ME, which matched the previous observation based on 16S rRNA gene [11]. Conversely, 38 specific phyla were observed only in MT samples, including four phyla belonging to the archaea domain, i.e., *Nanoarchaeota*, *Thermoplasmatota*, *Iainarchaeota*, and *Thermoproteota* (formerly known as *Thaumarchaeota*), and 34 bacterial phyla, such as *Chloroflexota*, *Marinisomatota*, *Nitrospirota*, *Planctomycetota*, *Elusimicrobiota*, *Hydrogenedentota*, and *Omnitrophota*.

Additionally, ME and MT shared a very small number of taxa, especially at a finer level (at or below the family) (Additional file 1: Fig. S6). For ~92% MAGs above middle-quality, the ANIs between genomes from ME and MT were all less than 80%, lower than the commonly accepted within-family cutoff of genome similarity [60, 61] (Additional file 1: Fig. S4). Furthermore, only eight genera were shared across ME and MT, including the genera *Acinetobacter* (15 MAGs), *Sphingomonas* (9 MAGs), *Brevundimonas* (5 MAGs), *Comamonas* (6 MAGs), *Phenylobacterium* (3 MAGs), *Pedobacter* (3 MAGs), and *Nocardioides* (9 MAGs) (Fig. 3A, Additional file 1: Fig. S6). At the species level, 29 and 99 species were unique to ME and MT, respectively (Additional file 1: Fig. S6). The only shared species was annotated as *Comamonas tsuruhatensis*, and the ANI was 96% among *C. tsuruhatensis* MAGs in ME and MT (Additional file 1: Fig. S4). The above results fully demonstrated that the taxonomic diversity of prokaryotes varied considerably between ME and MT and that most taxa at or below the family level are specific to either ME or MT.

### Distinct prokaryotic genomic and physiological characteristics between ME and MT

We further compared the genomic and physiological characteristics of prokaryotes between ME and MT based on all MAGs above middle quality (Fig. 3B–D, Additional file 1: Fig. S8A-C). The medium size of prokaryotic genomes from ME was ~ 4 Mbp, significantly larger than those from MT at ~ 3 Mbp (*Wilcox* test; *p* value < 1e−5) (Fig. 3B). The medium GC content of genomes in ME was ~ 57%, which is slightly higher than that in MT of ~ 56% (*Wilcox* test; *p* value < 0.05) (Fig. 3C). We also predicted the optimal growth temperature (OGT) according to MAGs above middle-quality as an indicator to represent the physiological features of prokaryotes at ME and MT (see the “Methods” section). These predictions showed that prokaryotes from ME had a significantly lower OGT than those from MT (*Wilcox* test; *p* value < 0.05), of which medium OGTs at 25 °C and 40 °C, respectively (Fig. 3D). The above results suggested significant differences in the genomic and physiological features of prokaryotes between these two distinct extreme environments.

### Similar metabolic capabilities between the communities in ME and MT

In contrast to the significant differences in taxonomic characteristics between ME and MT, the metabolic capabilities of prokaryotic communities were more conserved. The two groups for ME and MT were clearly separated in the nonmetric multidimensional scaling (NMDS) ordination of taxonomy below the family level (Fig. 2A, Additional file 1: Fig. S3). However, the NMDS ordination of KEGG orthologs (KO) overlapped between ME and MT (Fig. 2B). The dissimilarity of KO composition was significantly lower than the dissimilarity of taxonomic composition (*Wilcox* test, *p* value < 1e−5) (Fig. 2C). For metabolic modules, the dissimilarity between the two habitats was even as low as the cross-sample variation in ME (Fig. 2C). Similar trends were also revealed by MAG-based analysis (Additional file 1: Fig. S6, Fig. S7). The overlapped KOs between ME and MT occupied 73% of all annotated KOs from MAGs above middle quality, whereas the ME-specific and MT-specific KOs were only 10% and 17%, respectively. After mapping KOs to Kyoto Encyclopedia of Genes and Genomes (KEGG) pathway modules, 91% of mapped function modules were conserved, while only 1 and 8% were specific to ME and MT, respectively. These results imply that almost all metabolic capabilities are shared between ME and MT.

### Metabolic capabilities of prokaryotes in ME and MT

The MAG-based Sankey network of taxonomy and metabolism showed that distantly related microorganisms exhibited similar metabolic capabilities in ME and MT (Fig. 2D). To explore the relationship between metabolic capabilities and the taxa that perform them, we first mapped metabolic genes of all MAGs above middle-quality to metabolic pathways. Furthermore, we searched representative genes of target pathways against MAGs to obtain their taxonomic distribution in ME and MT (see the “Methods” section). We found surprising commonalities of metabolic capabilities between ME and MT, and distinct but various taxa could perform most metabolic pathways. We describe the essential functions that could be related to elemental cycling directly and adaptation to extreme stresses in the following paragraphs.

#### Carbon cycling

##### Degradation of refractory organic matters (ROMs)

**Fig. 4:**
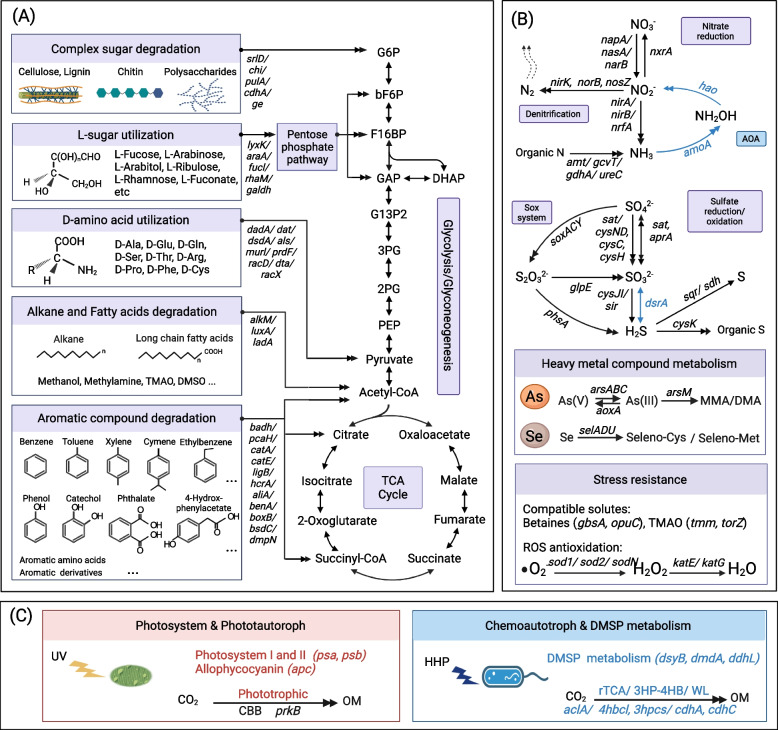
Schematic diagram of prokaryotic metabolic versatile in ME and MT. **A** Refractory organic matter utilization. **B** Metabolism of nitrogen, sulfur, heavy metal, and ROS. **C** Distinct pathways in ME and MT. Arrows represent biochemical reactions between two compounds. Double arrows indicate that multiple reactions are involved in the conversion between two shown compounds. Key genes in each pathway are shown in italic, which are corresponding to the gene IDs in Additional file 3: Table S2

**Fig. 5:**
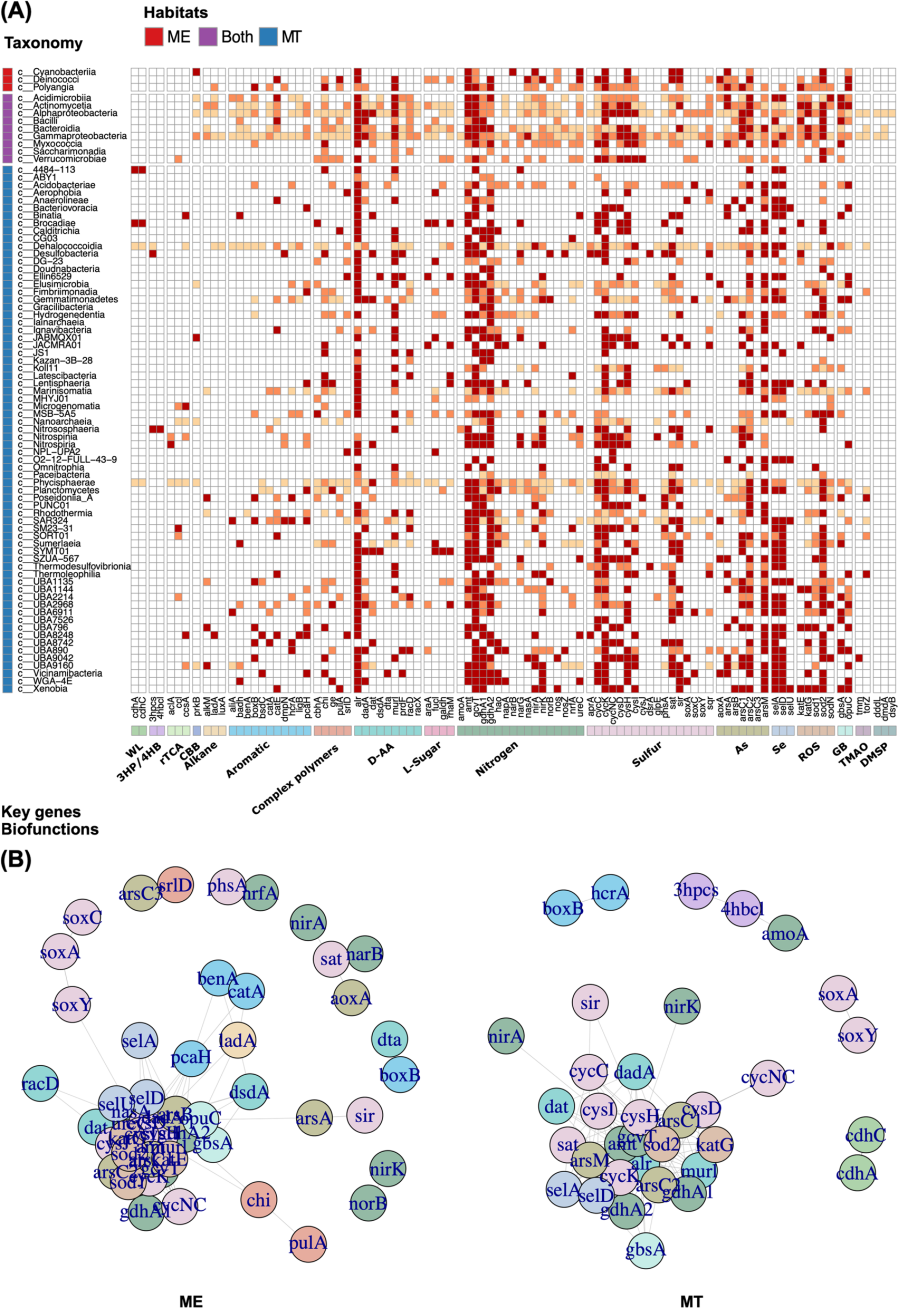
Distribution and co-occurrence of selected metabolic genes represented various biofunctions in MAGs. **A** Percentages of selected metabolic genes across class level in MAGs found in ME only, MT only, and both. Percentages of genomes of each class were shown in four levels: > 50% in dark red, 20–50% in orange, < 20% in light orange, while none in white. All MAGs used are above middle-quality (> 50% completeness and < 10% contamination). Key genes are corresponding to the gene IDs in Fig. 4 and Additional file 3: Table S2. **B** Co-occurrence network of selected metabolic genes in MAGs of ME or MT. All nodes with edges that binary-Jaccard distance > 0.3 are presented with circles. Colors of each node are corresponding to the color bar of biofunctions for selected metabolic genes in **A**

Prokaryotes in both ME and MT exhibited metabolic versatility to utilize complex organic matters, especially for those forms that are generally considered to be refractory, including complex polymers (e.g., cellulose, lignin, and chitin), aromatic compounds (e.g., benzene, aromatic hydrocarbons, aromatic amino acids, and other derivatives), alkanes (short and long chain), and abiotic enantiomers (D-amino acids and L-sugars). Utilization of these ROMs relied on enzymes to break the complex or rigid structures of ROMs at the first step and then through a series of enzymes to produce intermediates that are metabolic nodes linked to central carbon metabolism (Fig. 4A). Most representative genes involved in ROM degradation were not restricted to certain species or clades but had a wide distribution among taxa in both ME and MT (Fig. 5A, Additional file 1: Results, Additional file 3: Table S2). Notably, for D-amino acid (D-AA) metabolism, the catabolic capacities for nine D-AAs, including two typical D-AAs in bacterial cell walls (i.e., D-Ala, D-Glu) and seven non-canonical D-AAs (i.e., D-Gln, D-Ser, D-Thr, D-Arg, D-Pro, D-Phe and D-Cys), were found in MAGs from both ME and MT. Among them, *dadA*, the typical gene for a broad-spectrum D-AA dehydrogenase in D-AA catabolism [62], was abundant and found in 445 (of 1176) MAGs (59% in ME and 35% in MT). Almost all identified class-level clades (81/84) contain at least one D-AA metabolic gene(s) (Fig. 5A, Additional file 1: Fig. S9A, Additional file 3: Table S2). Like the observations that the same metabolic genes occurred in various taxa, the degradation of complex polymers, aromatic compounds, alkanes, and L-sugars were also found among varied taxa (Figs. 4A and 5A, Additional file 3: Table S2). This result suggested a wide distribution of ROM utilization capacity in prokaryotes of both ME and MT.

##### CO_2_ fixation

**Fig. 6:**
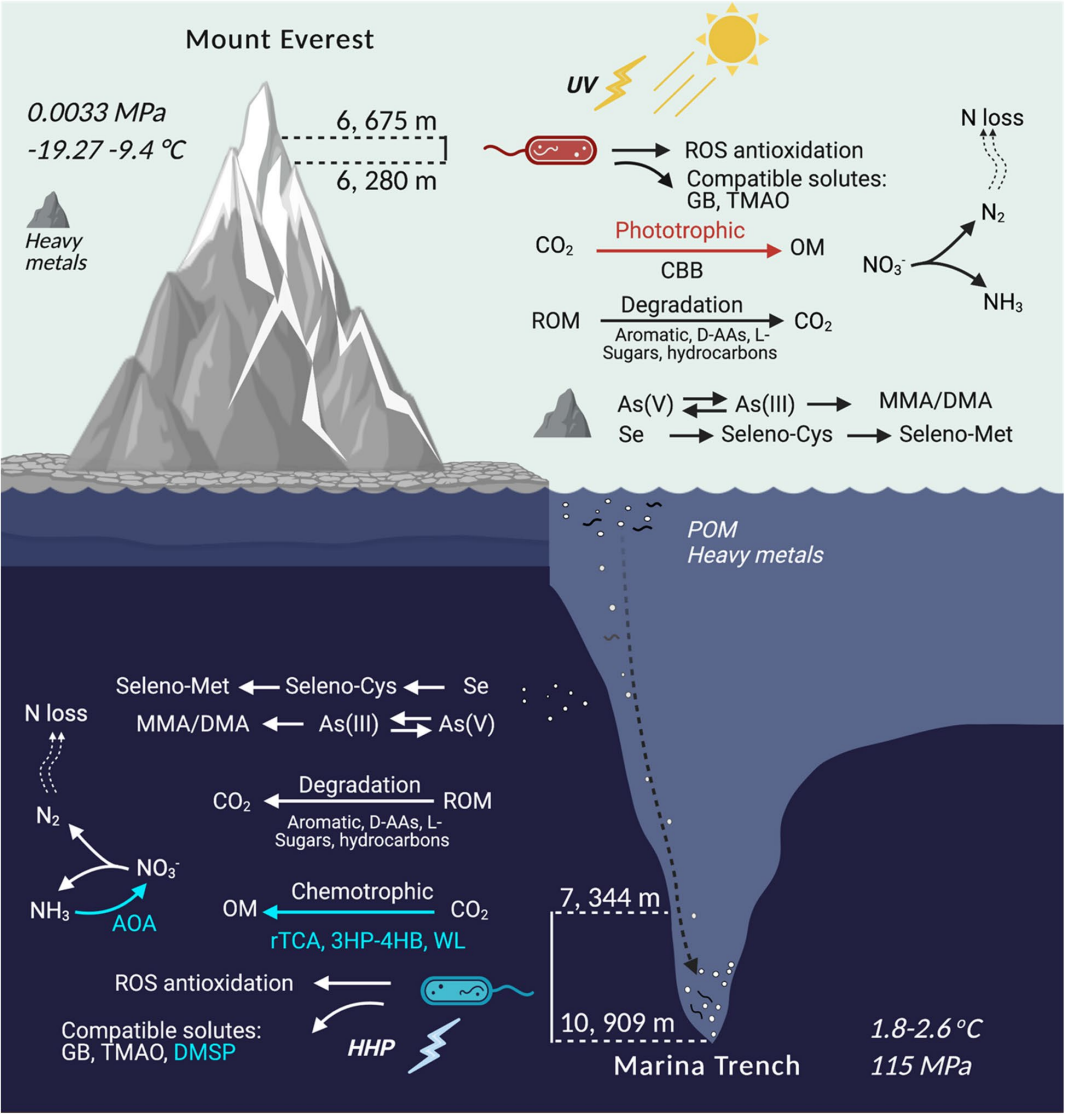
Schematic representation of major prokaryotic biofunctions compared between the ME and MT. ME-specific pathways are in red, while MT-specific pathways are in blue. OM, organic matter. ROM, refractory organic matter

Although evidence for autotrophic CO_2_ fixation to produce organic matters (OMs) was detected in both ME and MT, CO_2_ fixation-associated pathways were specific in ME and MT, (Figs. 4C, 5A, and 6, Additional file 3: Table S2). Genes involved in the typical chemoautotrophic pathways were only detected in metagenomes and MAGs of MT, including the Wood–Ljungdahl (WL) pathway (*cdhA* and *cdhC*), reductive Krebs (rTCA) cycle (*aclA*, *aclB*, *ccsA*, *ccsB*, *and ccl*), and 3-hydroxypropionate/4-hydroxybutyrate (3HP-4HB) cycle (*3hpcs* and *4hbcl*) (Additional file 1: Fig. S9A, Additional file 3: Table S2). Although gene for the Calvin–Benson–Bassham (CBB) cycle (*prkB*) was observed in both ME and MT, a series of genes in the photosystems I and II (*psaA-X* and *psbA-Z*) and allophycocyanin (AP, *apcA-F*) system, which is essential for photosynthesis, were only relatively complete in the ME-specific clade Cyanobacteria (Additional file 3: Table S2).

#### Nitrogen and sulfur cycling

Although our understanding of prokaryote-mediated nitrogen and sulfur cycling is still incomplete for both ME and MT, our results show that most of the known valence transition processes between the essential compounds of nitrogen and sulfur can be observed in the prokaryotic MAGs of both ME and MT (Fig. 4B). Detailed processes in nitrogen and sulfur metabolism and the taxa that perform them at ME and MT are described as follows.

##### Nitrogen metabolism

The pathways of dissimilatory nitrate reduction to ammonia (*napA/nasA/narB* and *nirA/nirB/nrfA*) and denitrification (*nirK, norB* and *nosZ*) are both complete in prokaryotic MAGs from ME and MT, which suggested the stable capacities of prokaryotic communities to use nitrate and nitrite as electron acceptors in ME and MT (Fig. 4B, Supplemental Text). The dissimilatory nitrate reduction was complete in 57% of the total MAGs in ME and 19% in MT, and the denitrification was only complete in 2 MAGs in ME and 7 MAGs in MT. These nitrate/nitrite respiratory genes are widely distributed among ME-specific, MT-specific, and cross-habitat class-level clades (Fig. 5A, Additional file 1: Fig. S9B, Additional file 3: Table S2). In addition, the representative gene of the cleavage system from organic nitrogen to ammonium, the *gcvT* gene for aminomethyltransferase, and the ammonium transporter gene *amt* had relatively high abundance in both ME and MT. Both *gcvT* and *amt* can be observed in more than 70% of MAGs covered in 65 class-level clades, which indicates the potential importance of recycling and exchanging nitrogen sources between cells and inorganic ammonium in both ME and MT.

Compared to prokaryotic functions in MT, the nitrogen cycling in ME is relatively incomplete, i.e., the aerobic ammonia oxidation pathway is missing according to both read-based and MAG-based analyses (Figs. 4B, 5A, and Fig. 6, Additional file 1: Fig. S9B). This is caused by the absence of the typical ammonia oxidation archaea (AOA), *Thaumarchaeota* (class *Nitrososphaeria*), from the prokaryotic community of ME. The representative gene *pmoA-amoA* for ammonia oxidation and genes *3hpcs* and *4hbcl* for the 3HP-4HB cycle, the specific CO_2_ fixation pathway in *Thaumarchaeota*, were only found in the MAGs of *Thaumarchaeota* from MT (Fig. 5A). Interestingly, the *hao* gene encoding hydroxylamine dehydrogenase was not only associated with the AOA but also found in 25 other MT-specific classes, which supported the previous assumption that the *hao* gene can be involved in other nitrogen processes in additional to aerobic ammonia oxidation [63].

##### Sulfur metabolism

Most genes in known sulfur metabolic processes can be found in MAGs of both ME and MT, including pathways of sulfate reduction (*sat*, *cysND*, *cysC*, *cysH* for assimilation, and *aprA* for dissimilation), assimilatory sulfite reduction (*cysJ*, *cysI*, and *sir*), sulfur oxidation (SOX) (*soxA*, *soxC*, *soxY*, and *glpE*), thiosulfate reduction (phsA), and sulfide oxidation to sulfur (*sqr* and *sdh*) (Fig. 4B, Additional file 1: Results, Fig. S9B).

However, the *dsrA* gene involved in converting sulfite to sulfide in dissimilatory sulfate reduction was only present in 12 MT-specific MAGs, restricted to two classes, *Desulfobacteria* and *Thermodesulfovibrionia* (Figs. 4B and 5A, Additional file 3: Table S2). *Desulfobacteria* is the typical sulfate-reducing bacteria (SRB) in subseafloor sediments [64], while *Thermodesulfovibrionia* is a recently reported sulfur-nitrogen coupling bacteria [65]. These results indicated a potentially essential role in the dissimilatory sulfate reduction in MT, but further investigation is still needed.

#### Heavy metal metabolism

Heavy metals, such as arsenate-As (V), arsenite-As (III), and selenium (Se), were reported to accumulate together with the sinking of POM by V-topology in MT [28, 66] and were also determined to be present in snow collected from the East Rongbuk Glacier in ME [67]. Therefore, it is reasonable to suppose that prokaryotes in both ME and MT possibly contained metabolic capabilities for heavy metal utilization and/or detoxication. Our MAG-based results supported this assumption. Indeed, many MAGs in both ME and MT with various class-level taxa harbored arsenic-related genes encoding arsenic detoxication (e.g., *arsC1* and *arsC2*), transport (e.g., *ArsB* and *Acr3*), and metabolism (e.g., *arsM* and *aoxA*) (Figs. 4B and 5A, Additional file 3: Table S2). Among them, representative genes for arsenate reduction from As(V) to As(III), *arsC1* (glutaredoxin) and *arsC2* (thioredoxin), were the most abundant among As-related genes in both ME and MT (Additional file 1: Fig. S9C). These two *arsC* genes were found in ~50% of total MAGs in either ME or MT. More than 80% of class-level clades harbored at least one *arsC* gene(s), indicating that the detoxication of As(V) is essential for prokaryotes in both ME and MT (Figs. 4B and 5A, Additional file 3: Table S2). Additionally, arsenic metabolism genes, e.g., *arsM* and *aoxA* (named as *aioA* in some studies), which are predicted to be less common than the *arsC* genes but highly endemic according to global soil microbiome analysis [68], were also found in both ME and MT. The *aoxA* gene for arsenite oxidation from As(III) to As(V) was found in a similar proportion (~ 10%) of total MAGs in both ME and MT. However, the assimilatory gene *arsM* for producing methylarsenite from arsenite [69] was found to account for higher coverage of total MAGs in MT (51%) than ME (8%) (Additional file 1: Fig. S9C, Additional file 3: Table S2).

Selenium (Se) is a trace element involved in the biosynthesis of selenium-containing amino acids, e.g., selenocysteine (Sec), most of which serve oxidoreductase functions [70]. Biosynthesis of Sec is not a common trait of prokaryotes [71]. Considering that the accumulation of Se (up to ~ 200 ng/g sample) has been reported in sediments of the bottom-axis of MT in a previous study [28], we found a relatively high abundance of representative Se-related genes among reads in MT as expected. Unexpectedly, Sec-related genes were also observed in ME, with a similar relative abundance to that in MT (Additional file 1: Fig. S9C), although the Se concentration in ME was measured at trace levels (45 ng/g sample) [72]. The selenophosphate synthetase gene *selD*, which is used to define the overall Se utilization from environments [71], was both overserved in 40% of total MAGs in ME and 47% in MT, being widely distributed in > 50% of class-level clades. A similar abundance of *selA*, *selB*, and *selU* genes encoding the synthesis of selenium-containing amino acids was observed in both ME and MT (Figs. 4B and 5A, Additional file 3: Table S2).

#### Stress resistance

##### Compatible solutes

The uptake and metabolism of compatible solutes is a typical strategy for stress adaptation across three domains of life. We found that these functional genes were shared in prokaryotes in both ME and MT (Figs. 4C and 5A, Additional file 1: Fig. S9C, Additional file 3: Table S2). Among all types of compatible solutes, glycine betaine (GB) is probably the most frequently used across three domains of life. The As-containing homolog of GB, arsenobetaine (As-GB), was reported to share the uptake and biosynthesis genes with GB and play a cytoprotective role against extremes in osmotic pressure and low temperature [73]. In our results, ~40% of total MAGs in ME and ~25% in MT harbored the representative gene *opuC* encoding transporters for both GB and As-GB, and ~28% of MAGs in ME and 33% in MT contained the representative gene *betB* encoding (arseno)betaine-aldehyde dehydrogenase to synthesize GB or/and As-GB. Both *opuC* and *betB* were distributed in various class-level clades (36%), including ME-specific, MT-specific and cross-habitat clades, indicating that genes for both the uptake and biosynthesis of GB and As-GB are widely distributed in prokaryotes in ME and MT.

In addition to the common compatible solutes mentioned above, prokaryotes in marine environments and deep-sea environments can synthesize or utilize some special compatible solutes for preserving macromolecules from high salinity and HHP environments, usually called as osmolytes or piezolytes. Trimethylamine N-oxide (TMAO) is a general piezolyte that commonly functions in both macro- and microorganisms from the deep sea [24, 74]. Interestingly, both biosynthesis and utilization genes for TMAO were found in MAGs from ME and MT (Figs. 4C, 5A, and 6, Additional file 1: Fig. S9C, Additional file 3: Table S2). The TMAO-producing gene *tmm* was encoded by 36 MAGs of MT (*Alphaproteobacteria*, *Gammaproteobacteria* and UBA9042) but was only found in one MAG of ME (*Alphaproteobacteria*). The relative abundance of the *tmm* gene among all clean reads in MT was significantly higher than that in ME (*Wilcox’s* test, *p* value < 0.001). The TMAO utilization gene *torZ* required to use TMAO as an anaerobic electron acceptor to produce trimethylamine (TMA) was also found in both ME and MT, with similar abundance (Additional file 1: Fig. S9C). However, the *torZ* gene had a wider distribution than the *tmm* gene, which can be found in 11 class-level clades. Among these classes, > 50% of MAGs belong to *Fimbriimonadia*, *Thermoleophilia*, and SORT01.

In contrast, *dimethylsulfoniopropionate* (DMSP)-related genes were restricted to prokaryotes in MT but not found in either the metagenome or MAGs from ME. DMPS is one of the most abundant organosulfur compounds in marine environments and has been reported to be produced in considerable amounts by bacterial cells in MT and act as a compatible solute in bacterial cells [75–77]. Representative genes involved in the biosynthesis (*dsyB*), demethylation (*dmdA*), and cleavage (*dddL*) of DMSP were only observed in MAGs of MT (Figs. 4C, 5A, and 6, Additional file 1: Fig. S9C, Additional file 3: Table S2). Among them, nine MAGs belonging to *Alphaproteobacteria* contained the DMSP-producing gene *dsyB*; 43 MAGs belonging to *Alphaproteobacteria*, *Gammaproteobacteria*, and *Bacterioidia* contained the DMSP demethylation gene *dmdA*; 14 MAGs belonging to *Alphaproteobacteria* and *Gammaproteobacteria* contained the DMSP cleavage gene *dddL* [78]. This result suggested a specific essential role of DMSP in MT ecosystems.

##### Antioxidation of reactive oxygen species (ROS)

Antioxidation was another shared pathway for stress resistance in prokaryotes across the ME and MT. Genes for typical ROS antioxidant enzymes, including superoxide dismutases, i.e., *sod1* (Cu-Zn family), *sod2* (Fe-Mn family), and *sodN* (Ni family), and catalase, i.e., *katE* and *katG*, were present in both ME and MT (Figs. 4B and 5A, Additional file 3: Table S2). Among them, the relative abundance of the *katE* and *sod2* genes in ME was significantly higher than that in MT; however, the Ni-*sodN* in MT was slightly higher than that in ME (*Wilcox’s* test, p value < 0.05) (Additional file 1: Fig. S9C). All of them were widely distributed in MAGs from ME and MT. The *sod2* gene had the highest coverage among all these tested ROS antioxidant genes, at ~91% of MAGs in ME and ~56% of MAGs in MT. Tested genes for ROS antioxidation were present at least one in ~80% of identified class-level clades. Our observations illustrated that ROS antioxidation is common and essential for prokaryotes regardless of whether they live at ME or MT.

### Distinct cooccurrence of metabolic functions across MAGs compared between ME and MT

Interestingly, the cooccurrence of different metabolic genes was quite different across prokaryotic genomes in ME and MT (see the “Methods” section, Fig. 5B, Additional file 1: Fig. S10). Generally, a closer correlation of the central clustered genes was observed in ME, while the cooccurrence of different genes in MT was more loosely correlated. Notably, in the cooccurrence network of ME, the degradation genes involved in aromatic compounds (*benA*, *catA*, *pcaH*), alkane (*ladA*), and D-AAs (*dsdA*) were correlated with each other and linked with the genes related to the universal compatible solute GB (*gbsA* and *opuC*) as nodes, and the degradation genes of complex polymers were correlated with functional genes involved in nitrogen, sulfur, and heavy metal metabolism. In contrast, there seems to be no such a cooccurrence of genes related to ROM utilization (except D-AA-related genes) among themselves or genes involved in other tested metabolic functions. In the cooccurrence network of MT, genes related to chemotrophic CO_2_ fixations were separated from the central cluster. For example, the gene *pmoA-amoA* for ammonia oxidation and genes *3hpcs* and *4hbcl* for the 3HP-4HB pathway, specific to *Thaumarchaeota* from MT, formed a separated cluster. A similar situation occurred for the *cdhC* and *cdhA* genes for the WL pathway in MT. The above results indicated that the combination of different metabolic modules, especially those related to different elemental cycling, may be specific to ME or MT. Despite the overall distinct cooccurrence network, regardless of ME or MT, the metabolic genes related to nitrogen, sulfur, As, and Se were highly correlated. This result indicated potential crosstalk among cycling of different elements in both ME and MT. In addition, the ROS antioxidation genes and GB biosynthesis gene (*gbsA*) were also observed in the central cooccurrence cluster, correlated with genes related to N, S, As, and Se, which suggested that prokaryotes in both ME and MT play an essential role in ROS antioxidation and biosynthesis of the universal compatible solute GB.

## Discussion

In this study, we investigated and compared prokaryotic communities by performing metagenome analysis between two geographically isolated extreme environments, ME and MT, the highest and deepest places on Earth, respectively. We observed significant differences in taxonomy between these two habitats, with many specific taxa that were only found in ME or MT. For taxonomy, our result that taxa tend to adapt and be specific to their habitats is consistent with previous global studies [6]. As the community composition is shown (Additional file 1: Fig. S1B), *Gammaproteobacteria* were the most abundant group in most samples of both ME and MT, which were reported to account for ~ 30% of glaciers [79, 80], ~60% in hadal trenches [81–84], and widely distributed in other cold environments, such as the Antarctic lakes and salt-cones [85, 86]. The shared genera between ME and MT, such as *Acinetobacter and Sphingomonas*, *were also reported as the dominant group in cold environments, including worldwide* glacier environments [87–91], clouds [92], and seawater [93]. *In addition,* the taxa specific to ME or MT we observed in this study are generally consistent with the typical prokaryotic clades in glaciers or trench environments, respectively. For example, the ME-specific phylum, *Cyanobacteria*, was reported as the pioneer colonizers of the glaciers, inhabiting ice surfaces [94], snow [95], and other glacier habitats [96, 97], playing a keystone role as the leading primary producer in the microbial food web in glacier environments (e.g., photosynthetic carbon and nitrogen fixation) [98–101]. Another ME-specific phylum we found in this study is *Deinococcota*, *which is* usually reported as radiation-resistant bacteria [21], and has been observed in ME-related glaciers under high UV conditions [12]. Similarly, the prokaryotic community composition observed in this study is quite comparable to other studies on the microbiome in hadal trenches. The MT-specific archaeal phylum, *Nitrososphaeria* (AOA), was the most abundance archaea in this study, which were also reported as the dominant archaea group with up to 30% in both water and sediment samples in hadal trenches [27, 102, 103].

This taxonomic variability revealed in this study provides clues to key environmental factors that affect prokaryotic life mostly in ME and MT. Our comparison revealed a significant difference in the genomic and predicted growth features of prokaryotes between ME and MT (Fig. 3, Additional file 1: Fig. S7). Although both ME and MT are low-temperature environments, the in situ temperature in MT is relatively stable (approximately 2 °C), while the temperature of ME, as mentioned above, is much more changeable (span 29 °C, ranging from −19.3 to 9.4 °C in summer) and varies with seasons and day/night cycles, which is also observed in other glacial ecosystem [104, 105]. The tolerance to temperature variations of prokaryotes in ME possibly requires special mechanisms with higher energy costs than adaptation to a stable low-temperature environment in MT, which may cause different genome size, GC contents, and growth features of prokaryotes. Moreover, growth temperature was reported to be closely related to mutation rate and population size in previous studies, especially under temperature fluctuations [106, 107]. Therefore, the difference between stable versus changeable in situ temperatures may cause distinct specialization or environmental selection mechanisms and processes in prokaryotes of ME and MT, which may be further reflected in the taxonomic diversity and composition of prokaryotic communities. Furthermore, bacterial genomes are commonly considered to tend to increase in size by aggregating adaptive gene modules to provide greater metabolic flexibility [108]. As alpha diversity showed above, prokaryotic communities in ME had lower diversity at a high taxonomic level (phylum) but had higher diversity at a low taxonomic level (species) than those in MT. The significantly higher genome size of prokaryotes in ME may be an explanation for why the prokaryotic community in ME has a lower taxonomic diversity than MT, but further investigation is needed. Finally, the cooccurrence network of versatile metabolic genes between ME and MT indicated that although the metabolic capabilities of the prokaryotic community are similar, distinct combinations of metabolic pathways are performed by different taxa, which could be a specific strategy for adaptation to distinct habitats. Although most metabolic pathways are shared between ME and MT and distributed among various taxa, some specialized functions are still restricted within certain taxa (i.e., AOA and SRB) and are only found in MT. These specific functions and the taxa that perform them indicated a potential essential role for ammonia oxidation in the nitrogen cycle and dissimilatory sulfate reduction in the sulfur cycle in MT.

In contrast to the taxonomy, to our surprise, prokaryotic metabolic capabilities exhibited more commonalities across these two extreme habitats, with > 90 metabolic modules overlapping. The Sankey network and taxonomic distribution of representative functional genes confirmed the inference of functional redundancy in which different taxa possibly harbor the same metabolic functions in ME and MT (Figs. 2D and 6). Although this functional redundancy phenomenon has been observed in some host-associated environments and bioreactors [11, 109–112], here is the first observation across such two distinguished and isolated extreme environments. The high species variability and metabolic commonalities between ME and MT make us rethink the relationship between taxonomy, metabolism, diversity, and adaptation of prokaryotes. These results may shed light on the principle of prokaryotic diversity: although taxa are specific to their habitats, primary metabolic functions could be still conserved.

These surprising metabolic commonalities between prokaryotes in ME and MT indicated underlying common strategies for prokaryotic adaptation to different extreme environmental conditions, although the geological topography and environmental conditions (e.g., sunlight, pressure, source and composition of nutrients) are intuitively different. For example, the stress of intense UV radiation in ME and the HHP in MT are seemingly incomparable at first glance. However, both UV and HHP can cause peroxidation damage for cells [22, 113], which makes antioxidants essential for prokaryotic adaptation in both ME and MT. This can explain why ROS antioxidation is common and widespread among diverse taxa of prokaryotes from both ME and MT (Fig. 5). Another example is the utilization of arsenic and selenic compounds, which is common in both ME and MT (Figs. 4, 5, and 6). A recent study reported a relatively high concentration (μg level) of As and Se in sediment samples from the bottom-axis of MT and pointed out the potentially important role of prokaryotes in redox balancing [28]. However, the concentration of As and Se in ME was reported to be two orders of magnitude lower than that in MT [72], and a similar relative abundance of genes enabling utilization of As and Se compounds was observed in ME in this study, which indicates a possible general requirement of cells for these two heavy metals and functions in their cycling [114–116]. Furthermore, both ME and MT can be considered nutrient-poor environments. The versatile metabolic genes encoding the ROM utilization enzymes in both ME and MT suggested a common strategy to survive under nutrient-poor environments where the ROM usually composes a more significant proportion of the organic matter than under normal conditions (e.g., in soil and surface seawater) [117]. Generally, a similar metabolic backbone and the basic biological rules are the basis of the common strategies for prokaryotes regardless of where they live.

## Conclusions

Although it is too early to draw a clean conclusion on how prokaryotic diversity formed and which factors influenced it, our results already suggest a possible relationship between prokaryotic taxonomy and metabolism: habitats tend to select the taxonomic composition of the prokaryotic community, while their metabolic functions could be very similar. This conclusion is valid at least in the comparison of prokaryotes between the highest and deepest habitats on Earth, and whether this conclusion could be extended to a broader scale still requires more investigations with a combination of a massive number of environmental parameter measurements, geochemical data, and multiple omics data for prokaryotes.

## Supplementary Information

**Additional file 1.** Results and figures.

**Additional file 2.** Dataset Table S1.

**Additional file 3.** Dataset Table S2.

**Additional file 4.** Workflow.

## Data Availability

The datasets generated during and/or analyzed during the current study are available in the NCBI repository in the accession number of projects PRJNA813429 and PRJNA859662. A full record of all statistical analysis is included as Additional file 4 and was created using the *knitr* package in R. All original scripts and result tables are available in GitHub (https://github.com/weishuzhao/ME-MT). Corresponding taxonomic classifications and information of metagenomic assembled genomes with annotations have all been included as Additional files 2 and 3, respectively.

## Abbreviations

2PG: D-Glycerate 2-phosphate
3HP-4HB: 3-Hydroxypropionate/4-hydroxybutyrate pathway
3PG: 3-Phospho-D-glycerate
Acetyl-P: Acetyl phosphate
AKG: 2-Oxoglutarate
ANI: Average nucleotide identity
AOA: Ammonia oxidation archaea
As-GB: Arsenobetaine
bF6P: Beta-D-fructose 6-phosphate
CBB: Calvin-Benson-Bassham cycle
D-AA: D-Amino acid
DHAP: Dihydroxyacetone phosphate
DMA: Dimethylarsinic acid
DMSP: Dimethylsulfoniopropionate
F16BP: Beta-D-fructose 1:6-bisphosphate
G13P2: D-Glycerate 1:3-diphosphate
G6P: D-Glucose 6-phosphate
GAP: Glyceraldehyde 3-phosphate
GB: Glycine betaine
GTDB: Genome Taxonomy database
HHP: High hydrostatic pressure
KEGG: Kyoto Encyclopedia of Genes and Genomes
KO: KEGG orthology
ME: Mount Everest
MMA: Monomethylarsonic acid
mOTU: Molecular Operational Taxonomic Unit
MT: The Mariana trench
NMDS: Nonmetric multidimensional scaling
OGT: Optimal growth temperature
OM: Organic matter
PEP: Phosphoenolpyruvate
POM: Particle organic matter
PSU: Practical Salinity Unit
ROM: Refractory organic matter
ROS: Reactive oxidative species
rTCA: Reductive tricarboxylic acid cycle
SRB: Sulfate-reducing bacteria
SOX: Sulfur oxidation
TMA: Trimethylamine
TMAO: Trimethylamine N-oxide
UV: Ultraviolet
WL: Wood–Ljungdahl
