# Supplementary material for: Comparison of prokaryotes between Mount Everest and the Mariana Trench
Source: Microbiome. 2022 Dec 7;10:215. doi: 10.1186/s40168-022-01403-y (PMC9727886; doi:10.1186/s40168-022-01403-y)
Supplement: Supplementary file 2 — Additional file 1. Results and figures. [file 40168_2022_1403_MOESM1_ESM.pdf]

Comparison of prokaryotes between the Mount Everest and the Mariana Trench

Yongqin Liu [1,3,9 #], Zhihao Zhang [3,9 #], Mukan Ji [1], Aoran Hu [2,7], Jing Wang [4,6,7], Hongmei Jing [5,8], Keshao Liu [3], Xiang Xiao [2,6,7,8 *], Weishu Zhao [2,6,7 *]

[#] These authors contributed equally to this article.

[*]**Correspondence:**

Xiang Xiao (zjxiao2018@sjtu.edu.cn) and Weishu Zhao (zwsh88@sjtu.edu.cn)

Affiliations

[1] Center for Pan-third Pole Environment, Lanzhou University, Lanzhou, China.

[2] State Key Laboratory of Microbial Metabolism, School of Life Sciences and Biotechnology, Shanghai Jiao Tong University, Shanghai, 200240, China.

[3] State Key Laboratory of Tibetan Plateau Earth System, Resources and Environment (TPESRE), Institute of Tibetan Plateau Research, Chinese Academy of Sciences, Beijing, China.

[4] School of Oceanography, Shanghai Jiao Tong University, Shanghai, 200240, China.

[5] Institute of Deep-Sea Science and Engineering, Chinese Academy of Sciences, Sanya, 572000, China.

[6] SJTU Yazhou Bay Institute of Deepsea Sci-Tech, Yongyou Industrial Park, Sanya, 572024, China.

[7] International Center for Deep Life Investigation (IC-DLI), Shanghai Jiao Tong University, Shanghai, 200240, China.

[8] Southern Marine Science and Engineering Guangdong Laboratory (Zhuhai), Zhuhai, Guangdong, China.

[9] University of Chinese Academy of Sciences, Beijing, China.

Supplemental Results

Utilization of refractory organic matters

Nitrogen and sulfur metabolism

Supplemental Figures

Fig. S1. Taxonomic annotation of mOTU of samples from ME and MT.

Fig. S2. Alpha-diversity indexes at different taxonomy levels.

Fig. S3. Beta-diversity at each classification level of taxonomy and KO in MT and ME samples.

Fig. S4. Distribution of the best average nucleotide identity (ANI) of MAGs between ME and MT.

Fig. S5. Distribution of sequence similarities between ME and MT.

Fig. S6. Venn plots of MAGs at each taxonomic level, KOs and metabolic modules comparation.

Fig. S7. Labeled Sankey network of taxonomy and function between ME and MT.

Fig. S8. Comparison among the MAGs belonging to ME-specific (red), MT-specific (blue) and cross-habitat classes.

Fig. S9. Abundancy of representative metabolic genes in samples from ME (red) and MT (blue).

Fig. S10. Co-occurrence trees of tested metabolic genes compared between ME and MT.

Supplemental Datasets and Tables (accompanying excel files)

Additional file 2: Table S1. mOTU table and rarefied table at species level and KO.

Additional file 3: Table S2. Distribution of all annotated KOs within MAGs above middle-quality in ME and MT.

Supplemental Results

Utilization of refractory organic matters

For complex polymers, cellulose and lignin are reported as the most abundant but typical refractory terrestrial biopolymers on Earth[1], and chitin is the most abundant refractory biopolymer in marine and has a number of different marine and terrestrial sources[2,3]. The representative *ge* encoding glucuronoyl esterase in degradation of lignin-cellulose[4] was found in 130 (of 1,176) of all MAGs of ME and MT, with similar proportion of MAGs in each habitat, 9% of total MAGs in ME and 11% in MT. These MAGs belong to 17 class-level clades, including 4 (of 9) cross-habitat classes (i.e., *Alphaproteobacteria*, *Bacteroidia*, *Gammaproteobacteria*, *Verrucomicrobiae*), and 13 (of 72) MT-specific clades (e.g.,

Acidobacteriae, *Planctomycetes*, *Hydrogenedentia*, etc) **(Fig. 5A, Additional file 3: Table S2).** The chitinase encoded gene *chi* for chitin degradation was found in 184 (of 1,176) MAGs in ME and MT samples (32% of total MAGs in ME and 14% in MT), distributed in 2/3 ME-specific classes (i.e., *Deinococci* and *Polyangia*), 21 (of 72) of MT-specific classes (e.g., *Desulfobacteria*, *Latescibacteria*, *Lentisphaeria*, *Marinisomatia*, etc) and all nine cross-habitat class-level clades (**Fig. 5A, Additional file 3: Table S2**). The similar scenario that same metabolic genes occur in various taxa was also found in degradation of aromatic compounds, alkanes, L-sugars and D-AAs (**Fig. 4A, 5A, Additional file 3: Table S2**).

Nitrogen and sulfur metabolism

The nitrate/nitrite respiratory genes are wide distributed among ME-specific, MT-specific and cross-habitat taxa (**Fig. 5A, Additional file 1: Fig. S9, Additional file 3: Table S2)**. For example, both the assimilatory gene *nasA* and the dissimilatory gene *napA* for nitrate reductase (from nitrate to nitrite) were relative abundant in both ME and MT. Gene *nasA* was found in 276 (of 1,176) MAGs (55% of total MAGs in ME and 20% of MT) while gene *napA* was found in 95 (of 1,176) MAGs (~ 11% of total MAGs in ME and 9% in MT) (**Fig. 5A, Additional file 3: Table S2)**. The *nirK* gene for nitrite reductase was found in 361 (of 1,176) MAGs (22% of total MAGs in ME and 32% in MT).

For sulfur metabolism, *sat* gene encoding sulfate adenylyltransferase in both assimilatory and dissimilatory sulfate reduction was found in 427/1,176 of MAGs (5% of total MAGs in ME and 40% in MT), distributed in 45 class-level clades. Genes for sulfite reductase, *cysI* (NADPH-dependent) and *sir* (ferredoxin-dependent) were found in 30% of total MAGs in ME and 40% in MT, distributed in 30 and 41 class-level clades, respectively.

Supplemental Figures

Fig. S1. Taxonomic annotation of mOTU of samples from ME and MT.

(A) Annotation percent of mOTU in different samples.

mOTUs are annotated by phyloFlash. Each color bar show percent of mOTUs in a sample that can be annotated to this taxon level but not to any lower taxon level. Horizontal lines show the minimum percent of mOTUs that can be annotated to the specific taxon level. For example, 83.48% mOTUs of any sample can be annotated at class level.

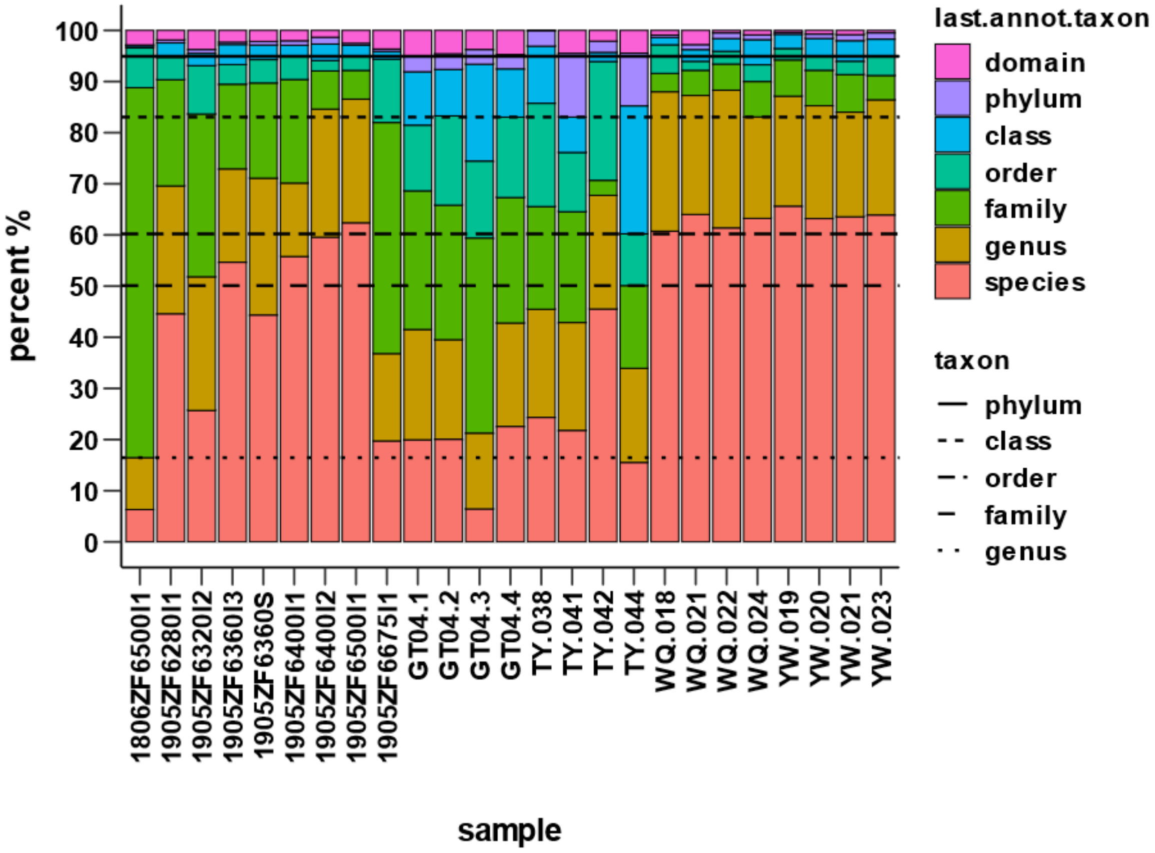

(B) Relative abundance of taxa to class level among samples from the MT and ME. mOTUs were annotated by phyloFlash. Any class with abundance not less than 5% in any sample are marked.

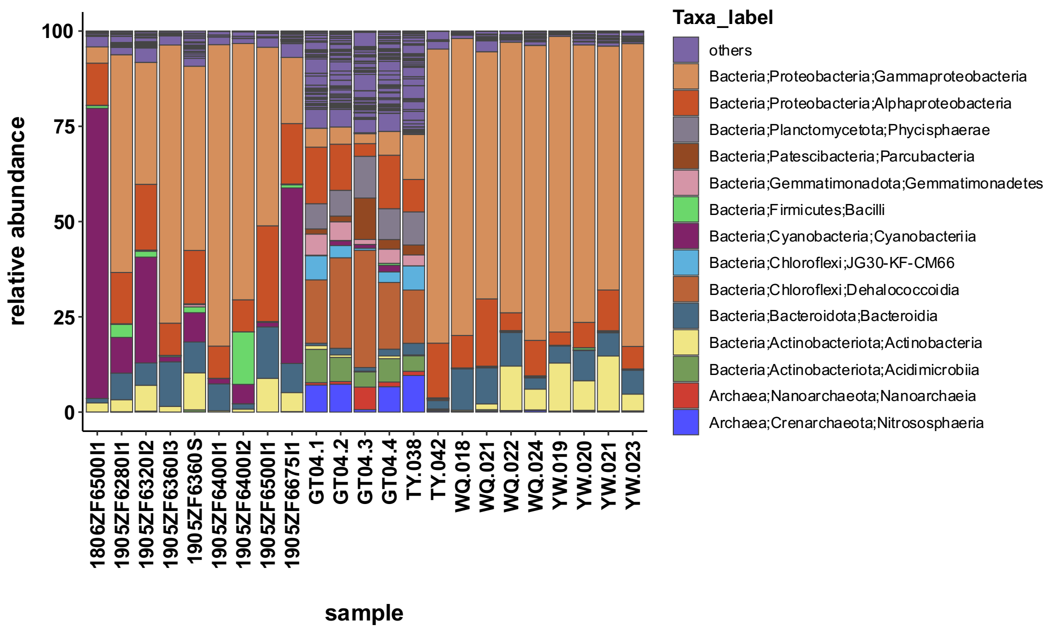

Fig. S2. Alpha-diversity indexes at different taxonomy levels. Shannon is in left and richness is in right. Indexes are based on annotation of phyloFlash at each level. Richness shows that MT and ME is significantly different at phylum, class, order and species level, but Shannon index shows no significant difference at any taxon level.

Shannon index **Richness**

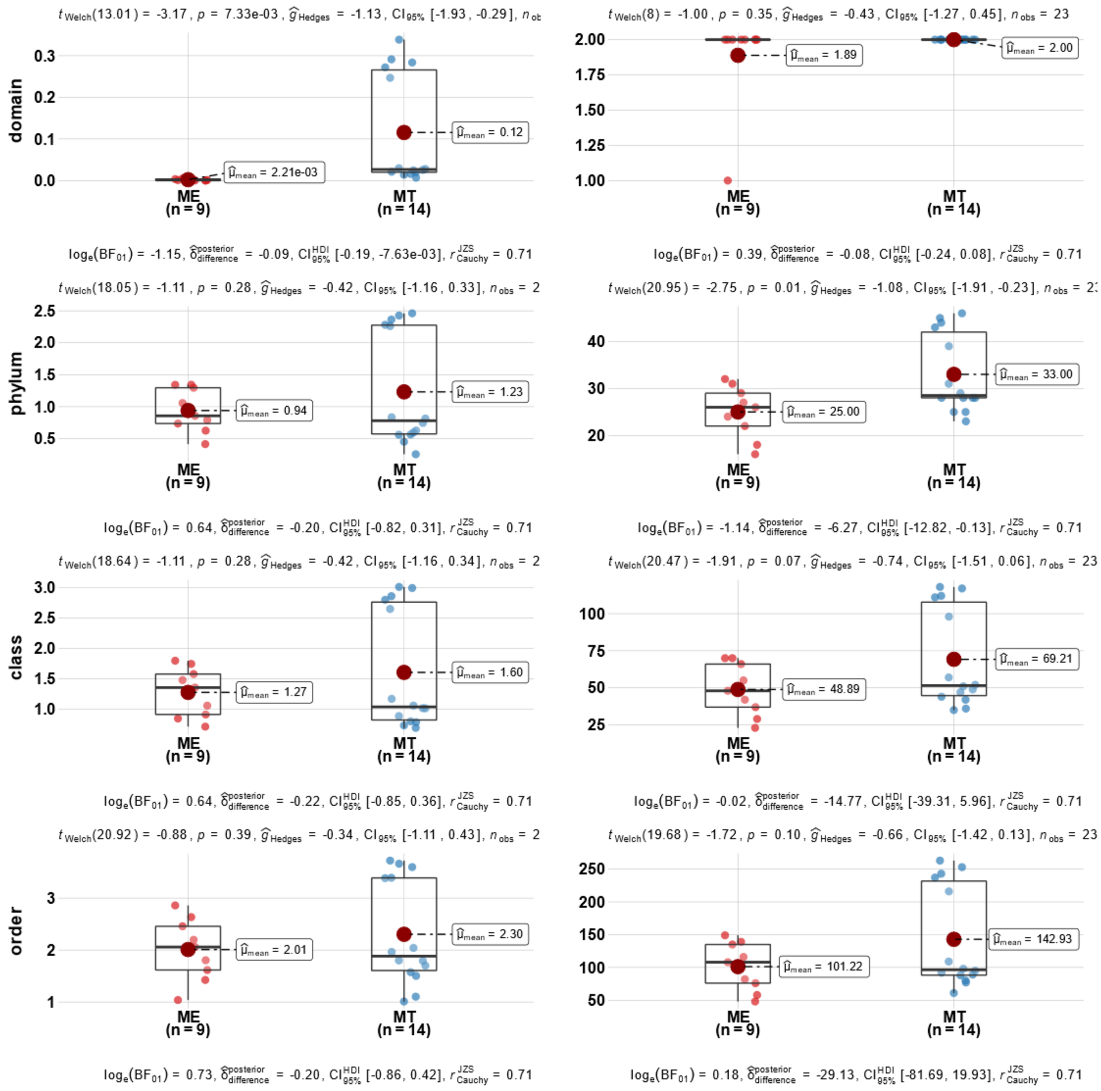

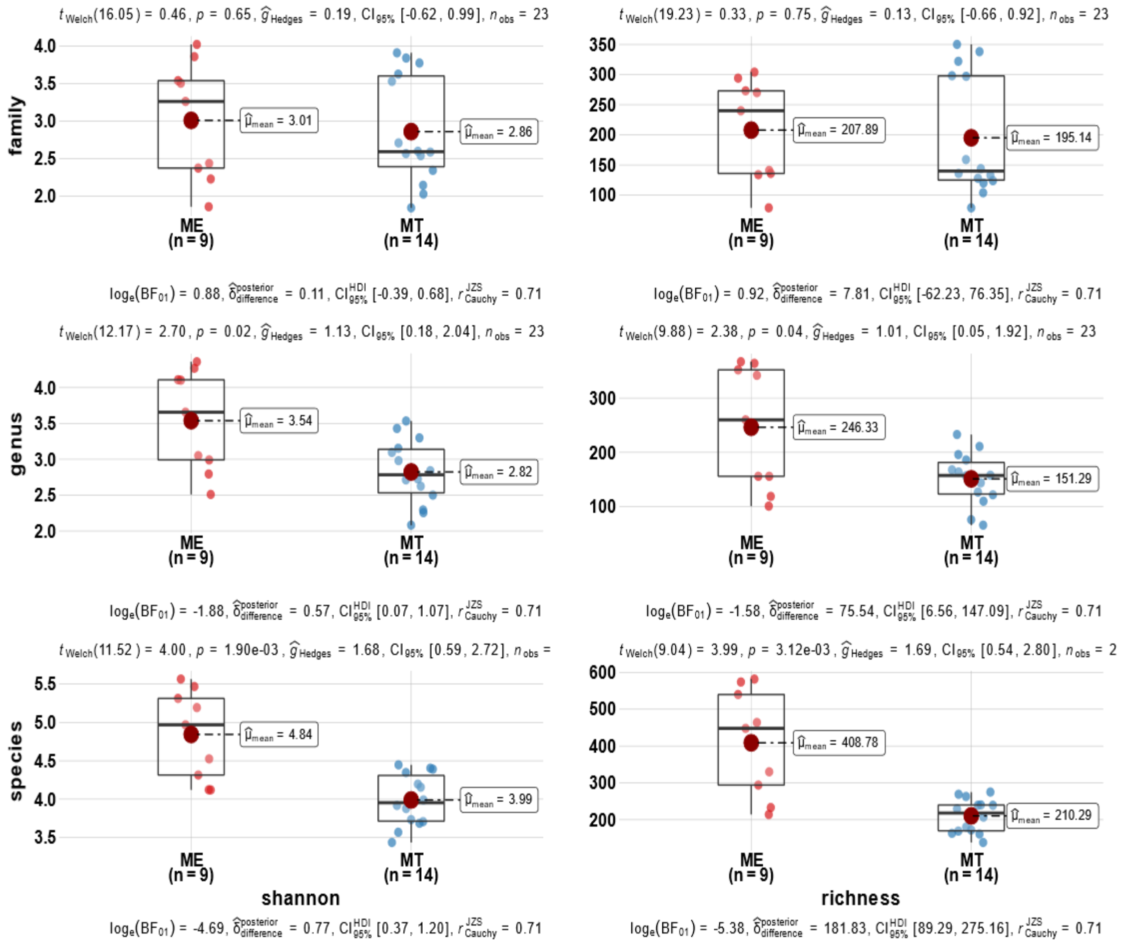
t_Welch(16.05) = 0.46, p = 0.65, g_Hedges = 0.19, CI95% [-0.62, 0.99], n_obs = 23
family
μ_mean = 3.01
μ_mean = 2.86
ME (n = 9)
MT (n = 14)
log_e(BF01) = 0.88, δ posterior difference = 0.11, CI HDI 95% [-0.39, 0.68], r JZS Cauchy = 0.71
t_Welch(19.23) = 0.33, p = 0.75, g_Hedges = 0.13, CI95% [-0.66, 0.92], n_obs = 23
μ_mean = 207.89
μ_mean = 195.14
log_e(BF01) = 0.92, δ posterior difference = 7.81, CI HDI 95% [-62.23, 76.35], r JZS Cauchy = 0.71
t_Welch(12.17) = 2.70, p = 0.02, g_Hedges = 1.13, CI95% [0.18, 2.04], n_obs = 23
genus
μ_mean = 3.54
μ_mean = 2.82
log_e(BF01) = -1.88, δ posterior difference = 0.57, CI HDI 95% [0.07, 1.07], r JZS Cauchy = 0.71
t_Welch(9.88) = 2.38, p = 0.04, g_Hedges = 1.01, CI95% [0.05, 1.92], n_obs = 23
μ_mean = 246.33
μ_mean = 151.29
log_e(BF01) = -1.58, δ posterior difference = 75.54, CI HDI 95% [6.56, 147.09], r JZS Cauchy = 0.71
t_Welch(11.52) = 4.00, p = 1.90e-03, g_Hedges = 1.68, CI95% [0.59, 2.72], n_obs =
species
μ_mean = 4.84
μ_mean = 3.99
shannon
log_e(BF01) = -4.69, δ posterior difference = 0.77, CI HDI 95% [0.37, 1.20], r JZS Cauchy = 0.71
t_Welch(9.04) = 3.99, p = 3.12e-03, g_Hedges = 1.69, CI95% [0.54, 2.80], n_obs = 2
μ_mean = 408.78
μ_mean = 210.29
richness
log_e(BF01) = -5.38, δ posterior difference = 181.83, CI HDI 95% [89.29, 275.16], r JZS Cauchy = 0.71

Fig. S3. Beta-diversity at each classification level of taxonomy and KO in MT and ME samples.

Left is used Bary-Cruit distance, while right is used binary Jaccard distance. NMDS ordination of taxonomy are based on SSU rRNA sequences at each classification level (from domain to species). SSU rRNA are assembled by phyloFlash from raw read. The ellipse shader shows the regression area of 95% confidence level. Genes are annotated by KEGG Ortholog (KO) and abundance is normalized as TPM (**Methods**). Red represents samples from ME, while blue represents samples from MT.

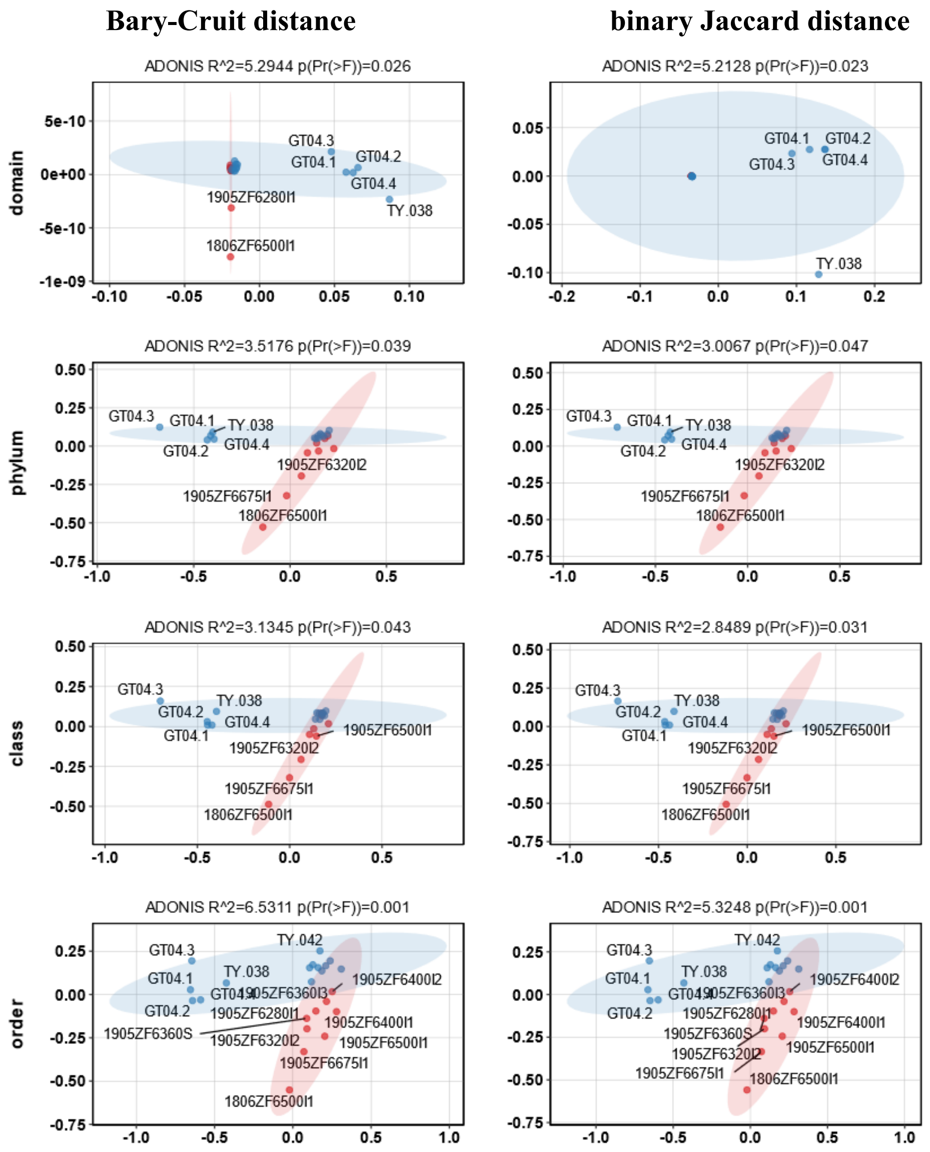

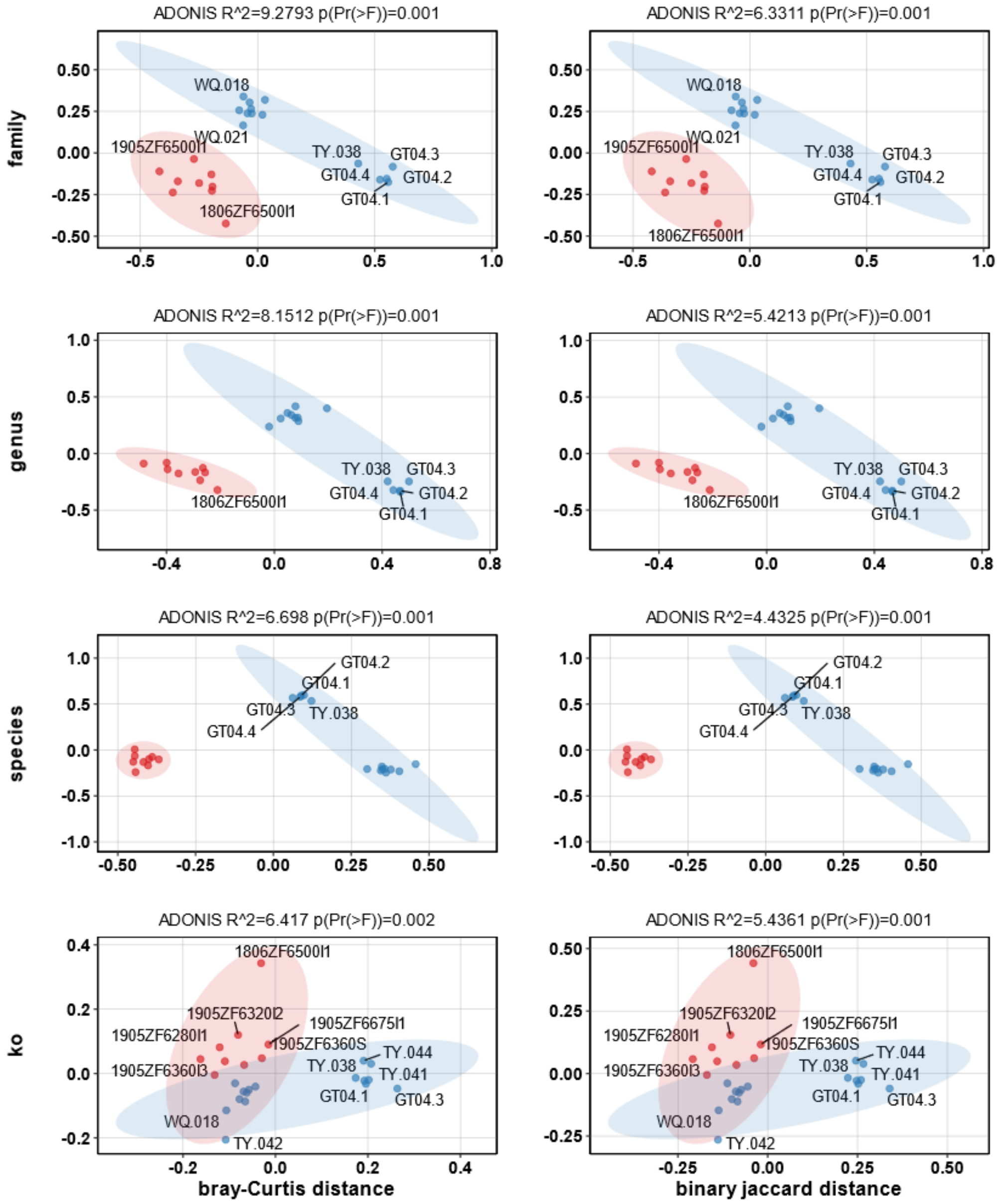
ADONIS R^2=9.2793 p(Pr(>F))=0.001
ADONIS R^2=6.3311 p(Pr(>F))=0.001
family
WQ.018
WQ.021
1905ZF6500I1
1806ZF6500I1
TY.038
GT04.3
GT04.4
GT04.2
GT04.1
ADONIS R^2=8.1512 p(Pr(>F))=0.001
ADONIS R^2=5.4213 p(Pr(>F))=0.001
genus
ADONIS R^2=6.698 p(Pr(>F))=0.001
ADONIS R^2=4.4325 p(Pr(>F))=0.001
species
ADONIS R^2=6.417 p(Pr(>F))=0.002
ADONIS R^2=5.4361 p(Pr(>F))=0.001
ko
1905ZF6320I2
1905ZF6280I1
1905ZF6675I1
1905ZF6360S
1905ZF6360I3
TY.044
TY.041
TY.042
GT04.1
bray-Curtis distance
binary jaccard distance

Fig. S4. Distribution of the best average nucleotide identity (ANI) of MAGs between ME and MT.

Each count represents a MAG with its the most similar MAG with the highest ANI against MAGs of the other location. ANI of MAGs between two habitats are calculated by fastANI. Be aware of that MAGs that share less than 80% ANI (brown dash line) and/or 30% of their gene content are too divergent to be compared based on the ANI measurement. MAGs that share more than 95% ANI (green line) are regarded as the same species.

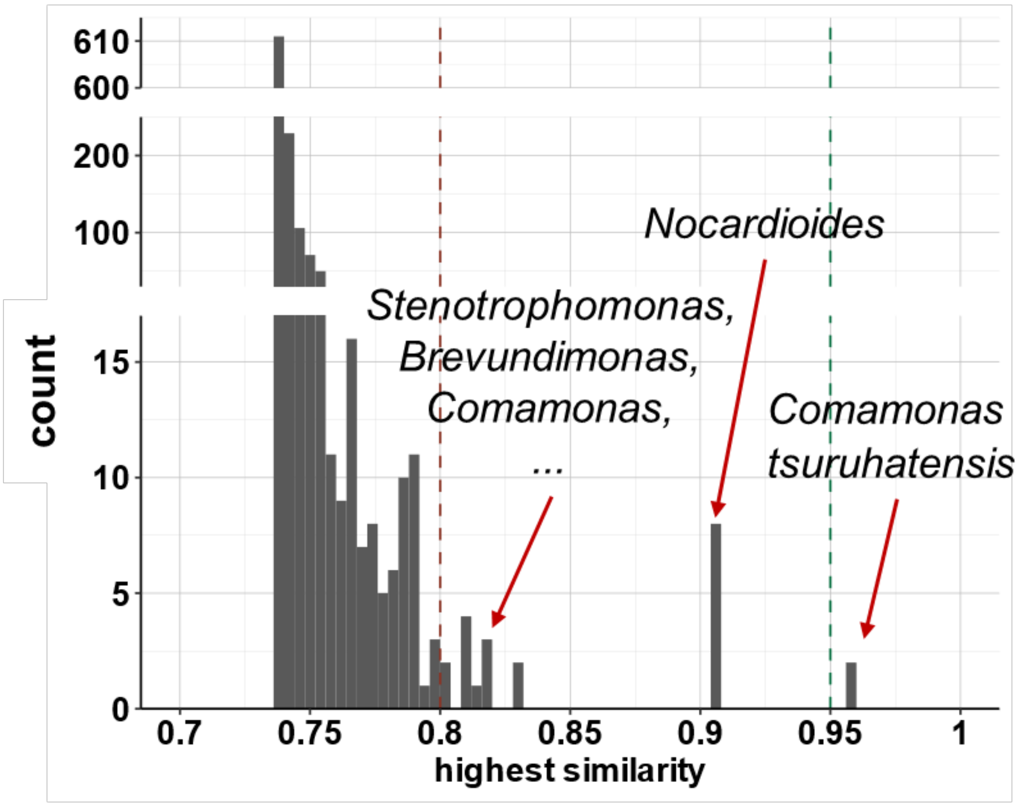

Fig. S5. Distribution of sequence similarities between ME and MT.

(A) Amino acid identity. (B) Nucleotide identity. Amino acid sequences from MT and ME are blast against each other using diamond with parameters "-e 0.00001 --id 30 --query-cover 50 --subject-cover 50 -b 4". 18674547 of total 29970697 sequences have at least one sequence of the same protein family in the other environment. Nucleotide sequence identity of gene pairs from amino acid sequence comparisons are calculated by blastn with parameters "-evalue 0.00001 -perc_identity 70 -qcov_hsp_perc 50".

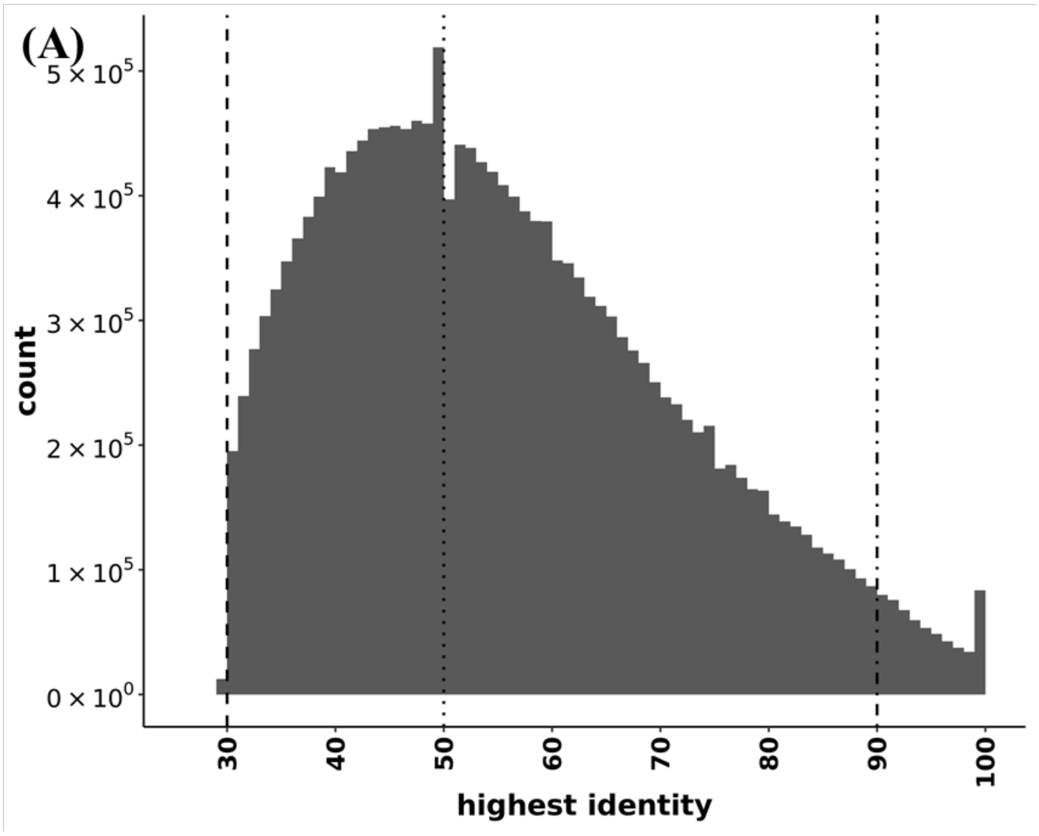

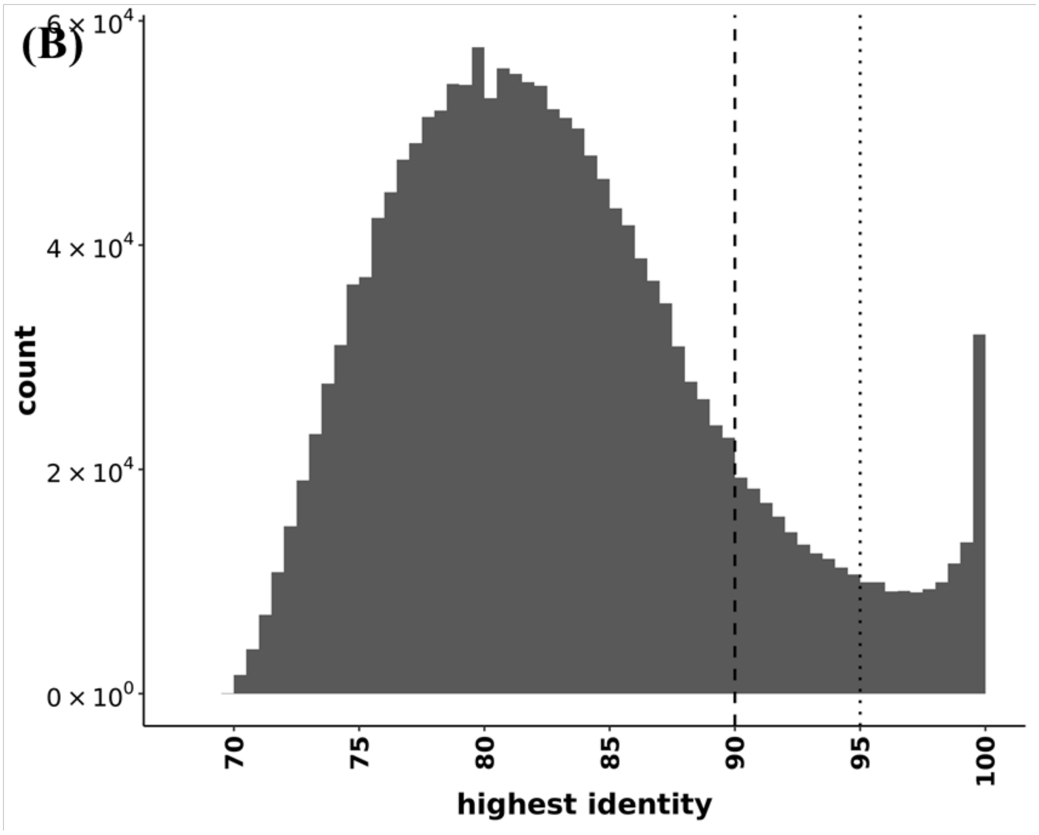
(B)
count
highest identity
6 × 10^4
4 × 10^4
2 × 10^4
0 × 10^0
70
75
80
85
90
95
100

Fig. S6. Venn plots of MAGs at each taxonomic level, KOs and metabolic modules comparation.

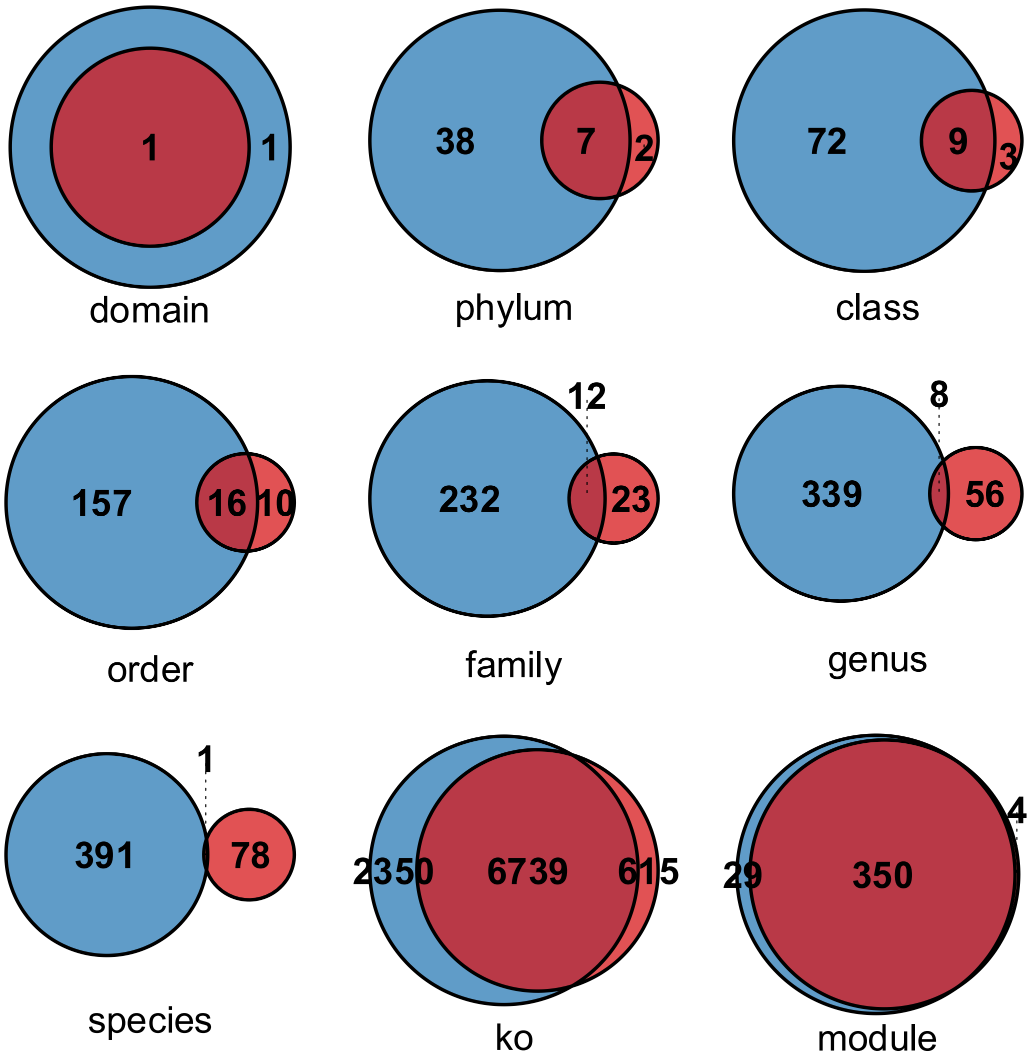

Fig. S7. Labeled Sankey network of taxonomy and function between ME and MT. This graph is corresponding to the Fig. 2D.

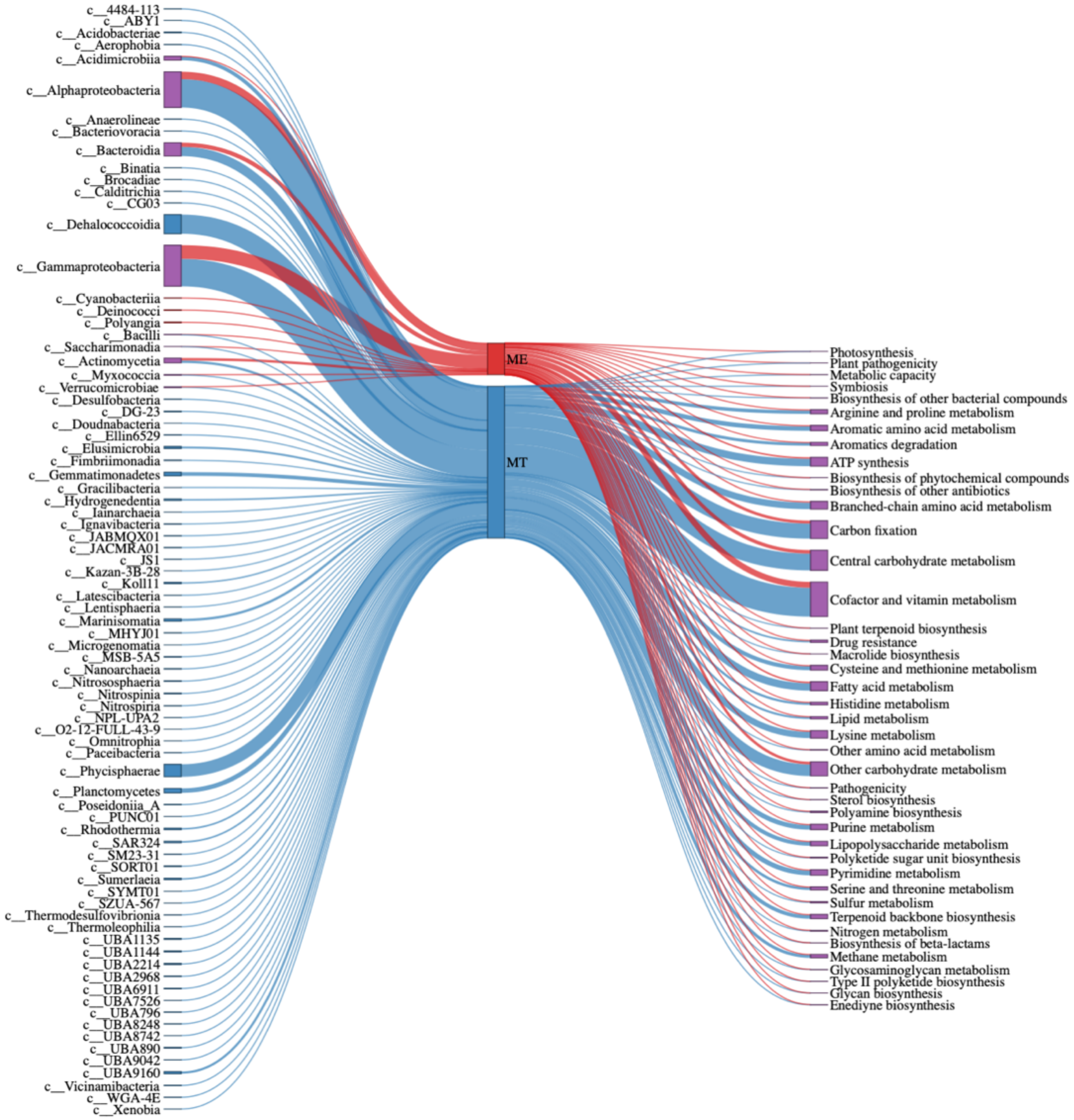

Fig. S8. Comparison among the MAGs belonging to ME-specific (red), MT-specific (blue) and cross-habitat classes. (A) GC content. (B) Genome size. (C) Predicted optimal growth temperature. Significant difference between ME and MT is using *ggsignif* package in R[5].

(A) GC content

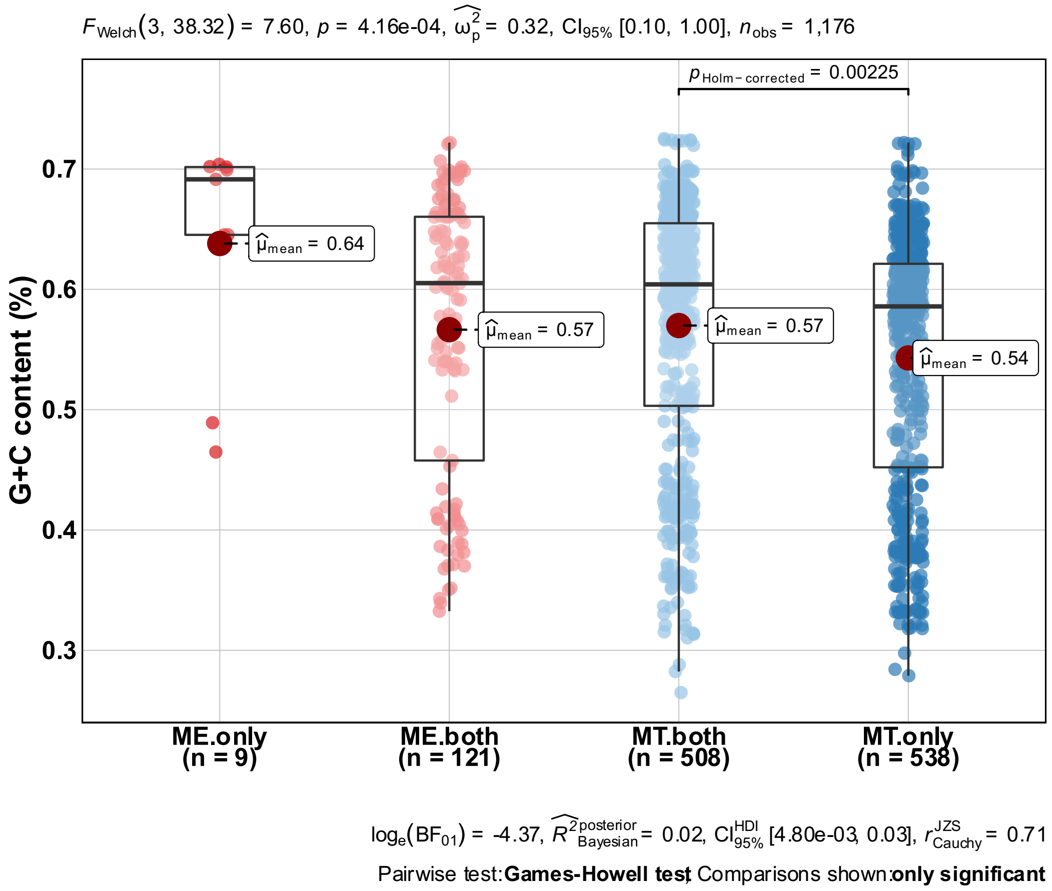

(B) Genome size

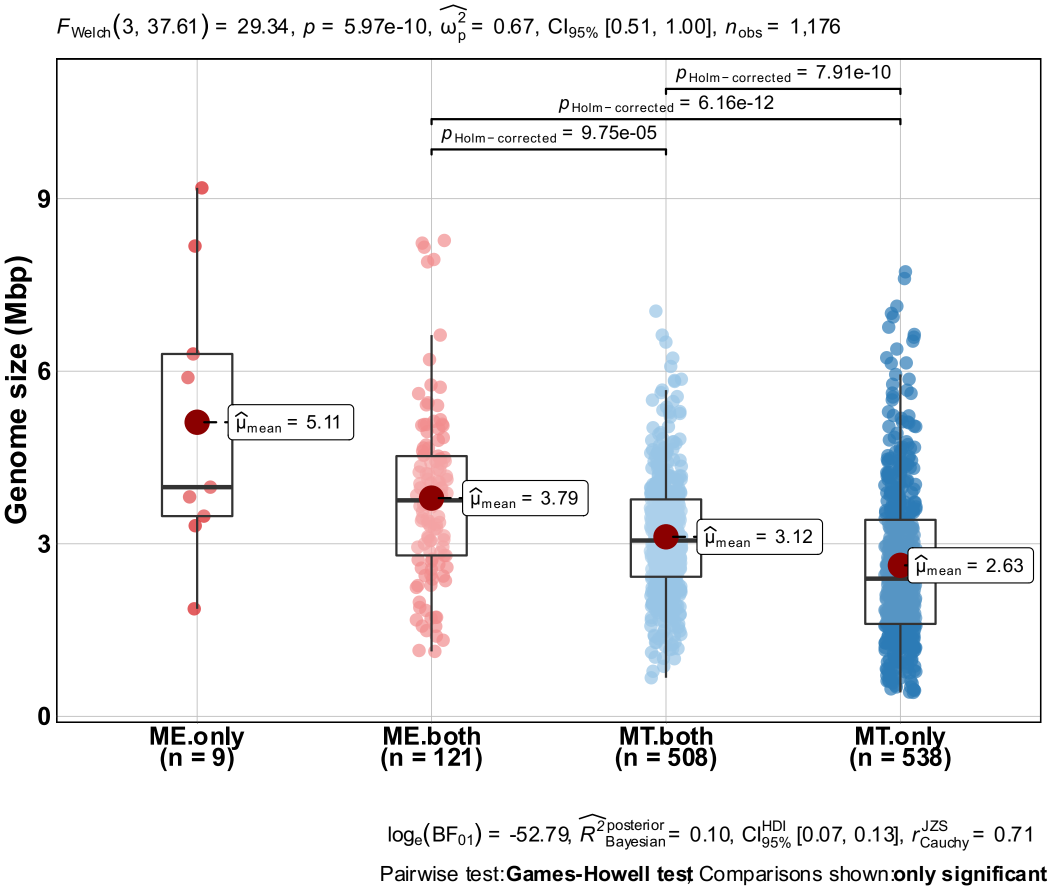

(C) Predicted optimal growth temperature

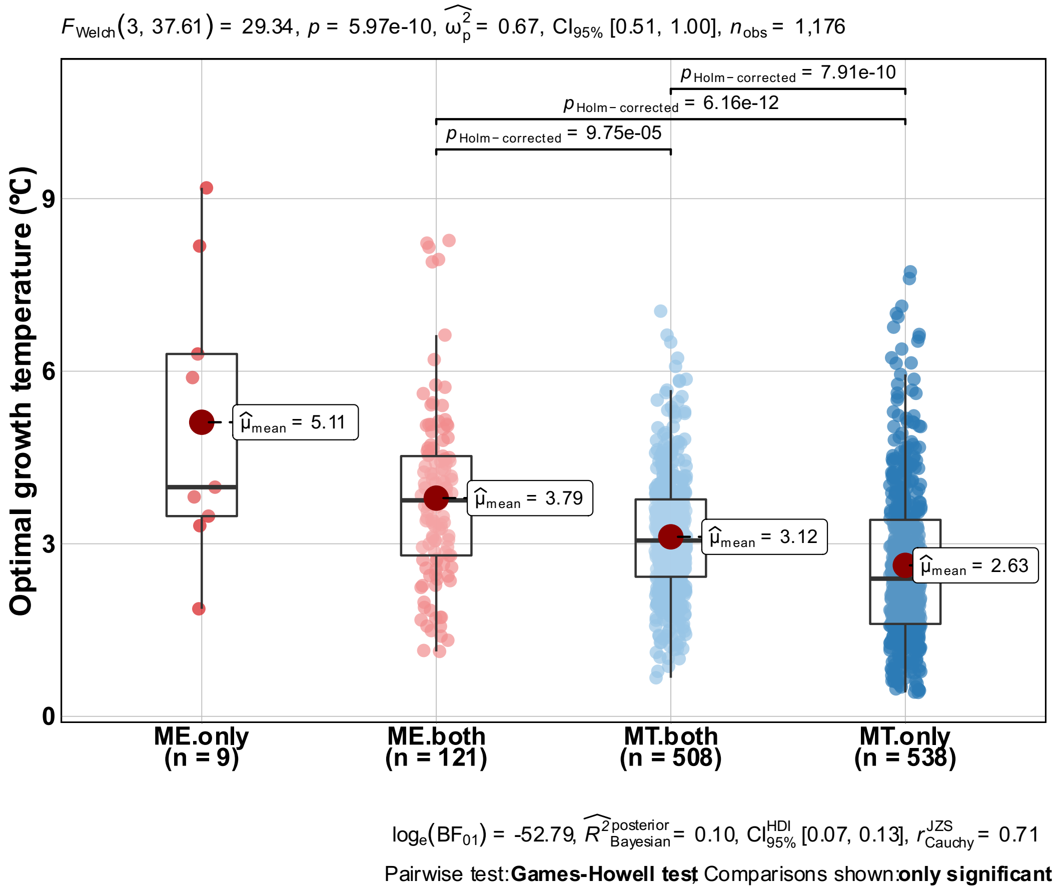

Fig. S9. Abundancy of representative metabolic genes in samples from ME (red) and MT (blue). Genes are corresponding to the gene IDs in Fig. 4, Fig. 5 and Additional file 3: Table S2.

(A) CO_2 fixation pathways and degradation of ROMs

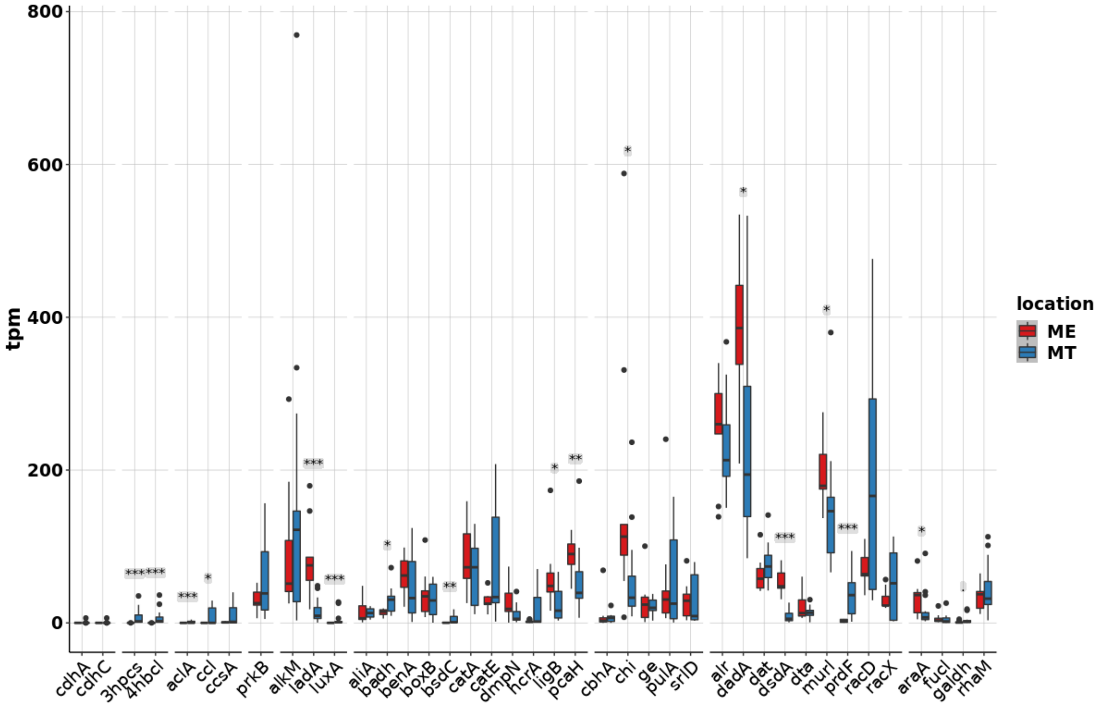

(B) Nitrogen and sulfur metabolism

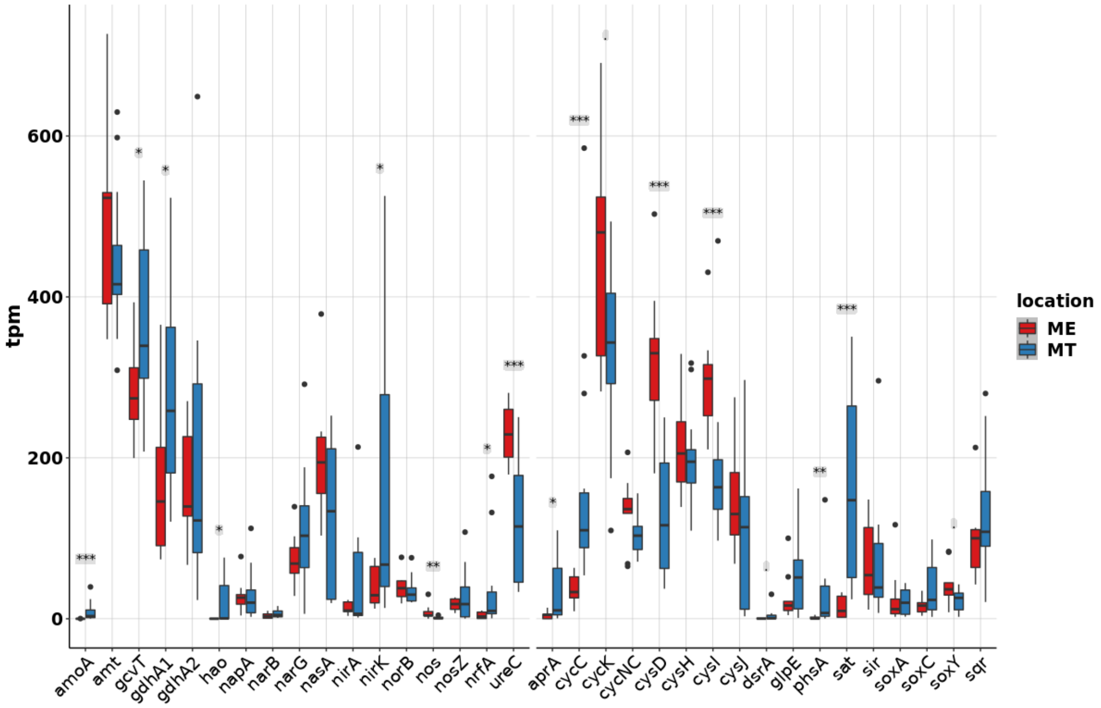

(C) As, Se, ROS, GB, TMAO and DMSP related genes

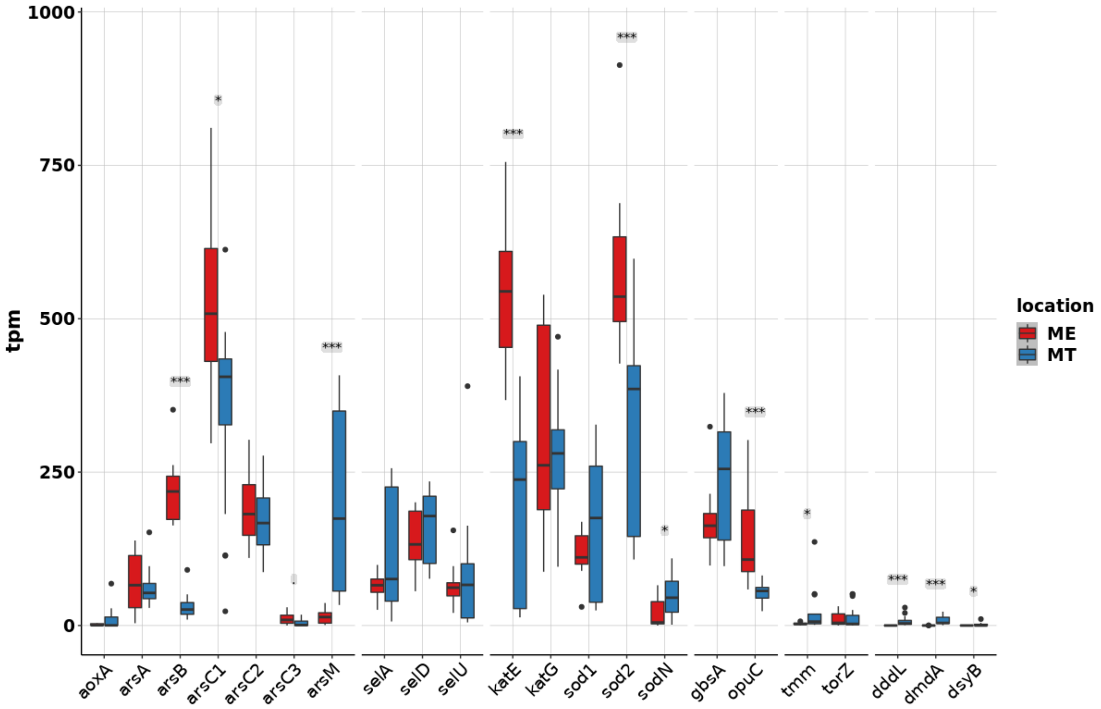

Fig. S10. Co-occurrence trees of tested metabolic genes compared between ME and MT. Colored branches represent the same correlation clusters between ME and MT; black branches represent the clusters that positions are different between ME and MT.

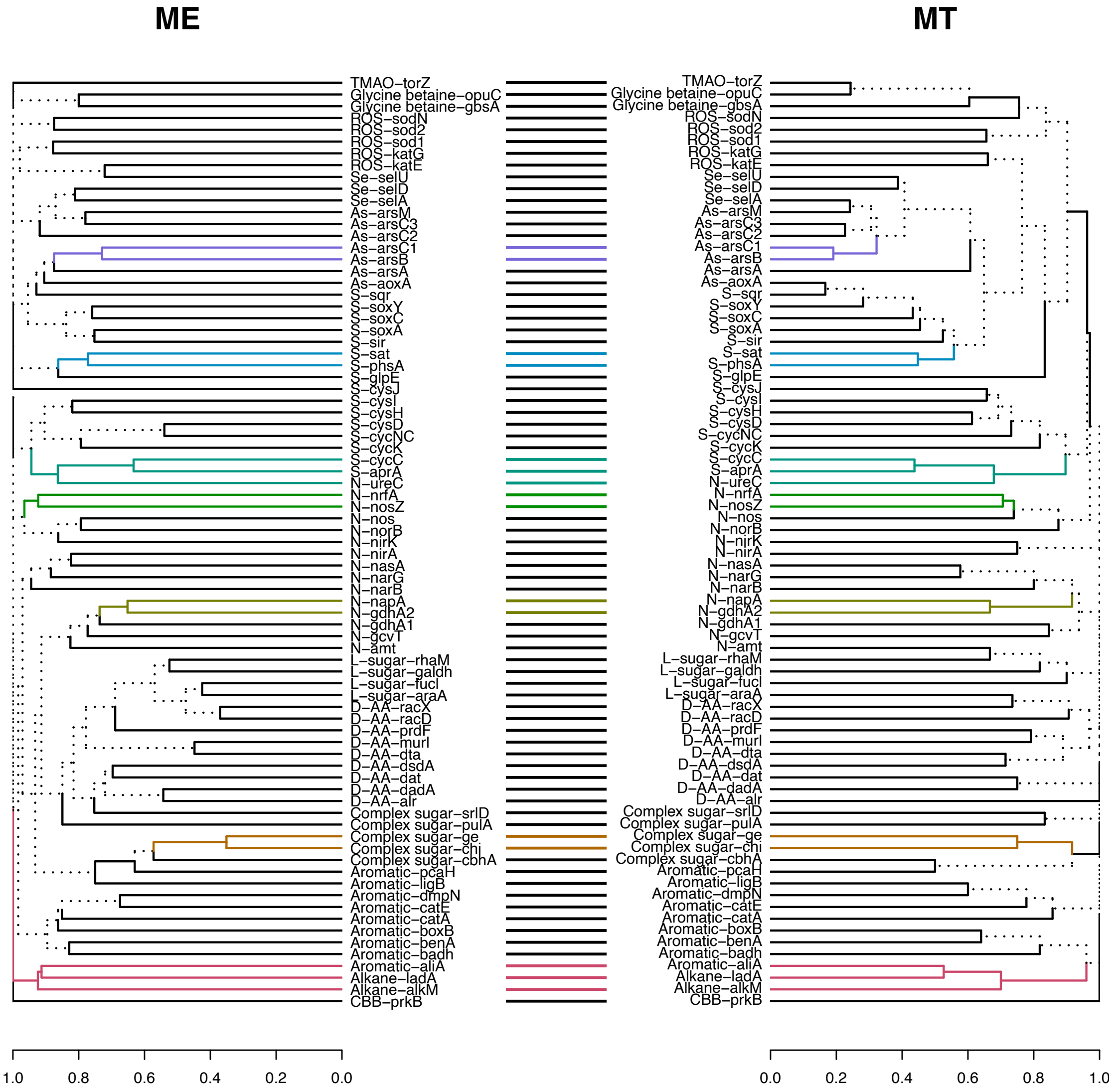

SI Reference:

1. Yu, TT, Wu WC, Liang WY, Lever MA, Hinrichs KU, et al. Growth of sedimentary bathyarchaeota on lignin as an energy source. Proc Natl Acad Sci USA. 2018; 115, 6022–6027.
2. Souza, C. P., Almeida, B. C., Colwell, R. R. & Rivera, I. N. G. The Importance of Chitin in the Marine Environment. Mar Biotechnol. 2011; 13, 823–830.
3. Hayes, M. Chitin, Chitosan and their Derivatives from Marine Rest Raw Materials: Potential Food and Pharmaceutical Applications. In: Hayes, M. editor. Marine Bioactive Compounds. Boston: Springe; 2012. p.115–128.
4. Zong ZY, Mazurkewich S, Pereira CS, Fu HH, Cai WS, et al. Mechanism and biomass association of glucuronoyl esterase: an α/β hydrolase with potential in biomass conversion. Nat Commun. 2022; 13, 1449.
5. Ahlmann-Eltze C, Patil I. ggsignif: R Package for Displaying Significance Brackets for ‘ggplot2’. 2021. PsyArXiv.
