# Supplementary material for: Comparison of prokaryotes between Mount Everest and the Mariana Trench
Source: Microbiome. 2022 Dec 7;10:215. doi: 10.1186/s40168-022-01403-y (PMC9727886; doi:10.1186/s40168-022-01403-y)

Comparison of prokaryotes between the Mount Everest and the Mariana Trench

This document contains all statistical analyses conducted for the manuscript. Note that due to the random iterative nature of some analyses (such as beta-rarefaction & NMDS) some of the figure parameters will change slightly during reanalysis, though core results will remain essentially unchanged.

All data to reproduce analysis can be found here: `https://github.com/weishuzhao/ME-MT` , and files that will be used are listed below:

- `div.phyloflash.rarefy.csv` : collected `phyloflash` output table
- `ko_reads_zone.csv` : a long table indicating gene abundance in each sample, and gene similarity across environment.
- `module_reads_zone.csv` : similar to `ko_reads_zone.csv` but summary by KEGG module.
- `Wtdb.csv` : features of representative MAGs of each cluster, including complement, containment, site, classification, etc.
- `MAG_info.csv` : features of all MAGs recovered in this study.
- `genomeko1.csv` : function annotations of MAGs, `1` represents to one gene.
- `ko_label.tsv` : key gene list.

load environment

```
## Writing NAMESPACE
## Writing NAMESPACE
```

Reads diversity

Annotation level estimated by phyloFlash

```
div.lastannot = sapply(
  as.character(taxon.levels),
  function(x)
    apply(div.phyloflash.raw[!taxon.split(rownames(div.phyloflash.raw), x) %>%
                           grepl(pattern = "^\\([^;]+\\)$", .),],  # > 0,#,
          2, sum))
label = "last.annot.taxon"
ce = bar.pct.annot(div.lastannot %>%
                     apply(., 1, function(x) x - c(x[-1], 0)),
                   factor(taxon.levels, levels = rev(taxon.levels)))
ce[label] = ce$name
div.lastannot.min = div.lastannot %>%
  (function(x) x / x[, 1] * 100)(.) %>%
  apply(., 2, min)


p =
  ggplot(data = ce, mapping = aes_string(x = "sample", y = "annot.percent",
                                          fill = label)) +
  geom_bar(position = position_stack(reverse = T),
           stat = "identity",
           col = "black", size = 0.3) +
  guides(fill = guide_legend(reverse = TRUE), alpha = "none") +
  scale_y_continuous(breaks = seq(0, 100, 10)) +
  labs(title = "", x = "sample", y = "percent %") +
  theme(
    axis.text.x = element_text(angle = 90, hjust = 1, vjust = 0.5),
    plot.title = element_text(hjust = 0.5)
  ) +
  geom_hline(
    data = data.frame(div.lastannot.min,
                      taxon = factor(names(div.lastannot.min),
                                     levels = taxon.levels))[2:6,],
    mapping = aes_string(yintercept = "div.lastannot.min",
                         linetype = "taxon"),
    size = 0.7)

p
```

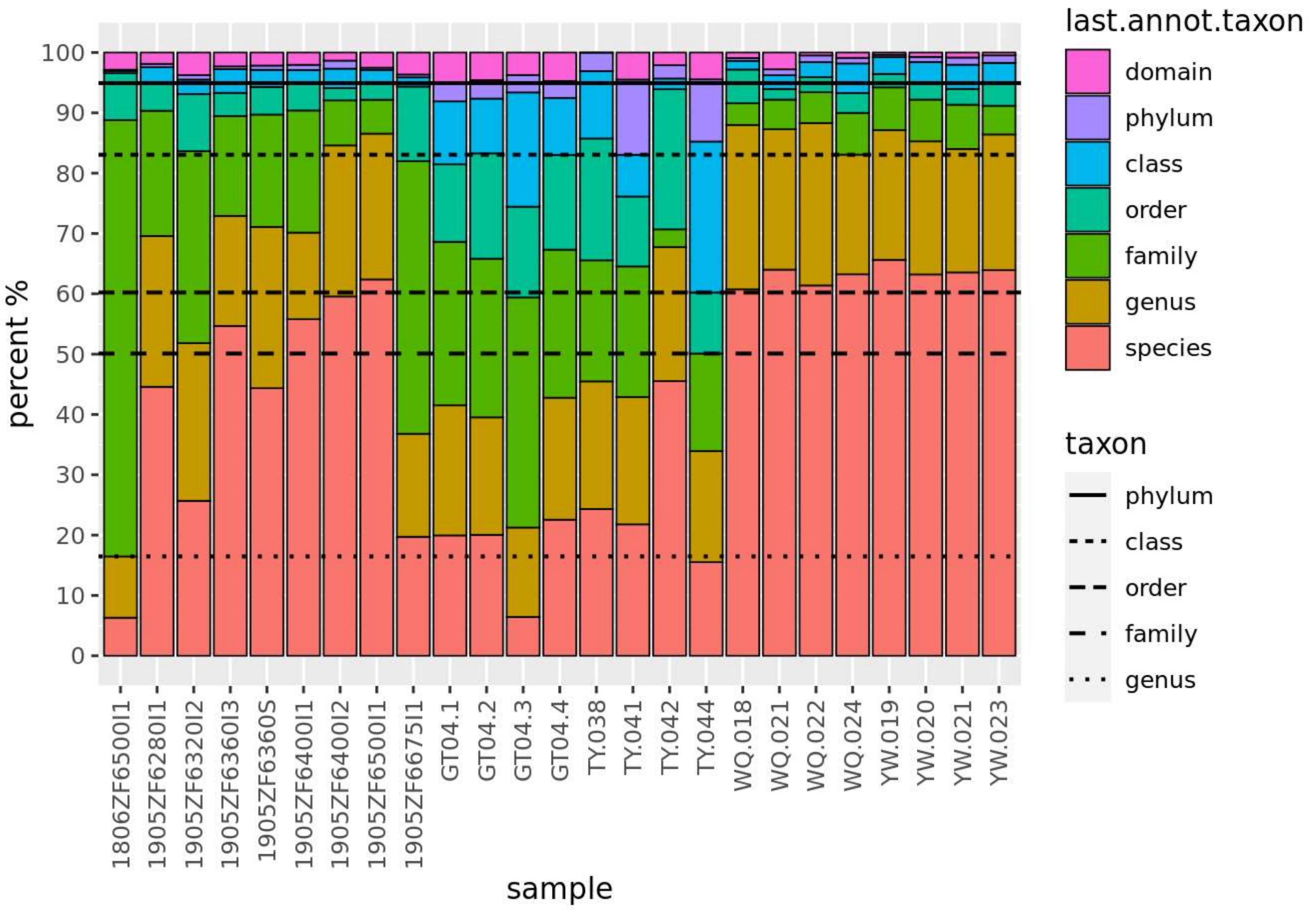

A total of 1841892 reads were mapped to 4084 mOTU sequences at the species level or higher based on the MiTAG method using phyloFlash (v3.4).

~50% of family-level and ~80% of genus-level mOTUs were unclassified according to the SILVA 138.1 database.

- rarefy raw phyloflash results

```
div.phyloflash.rarefy =
  div.phyloflash.raw %>%
  {t(.)} %>%
  {.[apply(., 1, sum) >  30000, ]} %>%
  {rrarefy(., apply(., 1, sum) %>% min)} %>%
  {t(.)}
```

Alpha diversity estimated by phyloFlash

```
p_all <- NULL
for (taxon.level.spec in c(taxon.levels)) {
  div.otu = collapse.div_to_taxon(div.phyloflash.rarefy, taxon.level.spec)

  p1 = ggbetweenstats.local1(div.otu = div.otu, method = "shannon")
  p1$labels$x = "shannon"
  p1$labels$y = taxon.level.spec

  p2 = ggbetweenstats.local1(div.otu = div.otu, method = "richness")
  p2$labels$x = "richness"
  p2$labels$y = ""
  print(p1 + p2)

  p1$labels$x = {if (taxon.level.spec == "species") {"shannon"} else {""}}
  p2$labels$x = {if (taxon.level.spec == "species") {"richness"} else {""}}

  if (is.null(p_all)) {p_all = p1 + p2} else {p_all = p_all + p1 + p2}
}
```

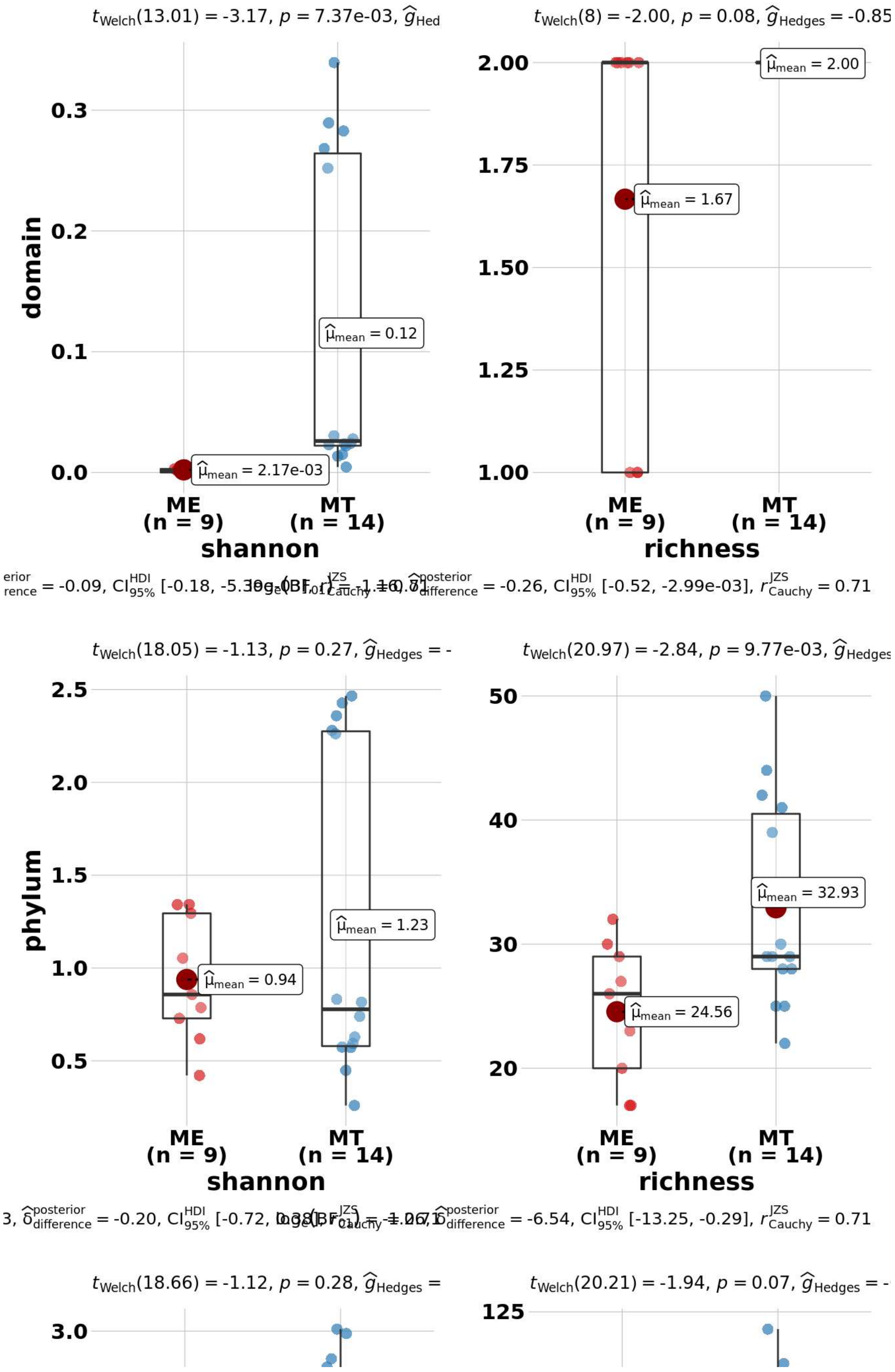

$t_{Welch}(13.01) = -3.17$, $p = 7.37e-03$, $\widehat{g}_{Hed}$
$t_{Welch}(8) = -2.00$, $p = 0.08$, $\widehat{g}_{Hedges} = -0.85$
domain
$\widehat{\mu}_{mean} = 0.12$
$\widehat{\mu}_{mean} = 2.17e-03$
$\widehat{\mu}_{mean} = 2.00$
$\widehat{\mu}_{mean} = 1.67$
ME (n = 9)
MT (n = 14)
shannon
richness
$= -0.26$, $CI_{95\%}^{HDI}$ [-0.52, -2.99e-03], $r_{Cauchy}^{JZS} = 0.71$
$t_{Welch}(18.05) = -1.13$, $p = 0.27$, $\widehat{g}_{Hedges} = -$
$t_{Welch}(20.97) = -2.84$, $p = 9.77e-03$, $\widehat{g}_{Hedges}$
phylum
$\widehat{\mu}_{mean} = 1.23$
$\widehat{\mu}_{mean} = 0.94$
$\widehat{\mu}_{mean} = 32.93$
$\widehat{\mu}_{mean} = 24.56$
$\widehat{\delta}_{difference}^{posterior} = -6.54$, $CI_{95\%}^{HDI}$ [-13.25, -0.29], $r_{Cauchy}^{JZS} = 0.71$
$t_{Welch}(18.66) = -1.12$, $p = 0.28$, $\widehat{g}_{Hedges} =$
$t_{Welch}(20.21) = -1.94$, $p = 0.07$, $\widehat{g}_{Hedges} = -$

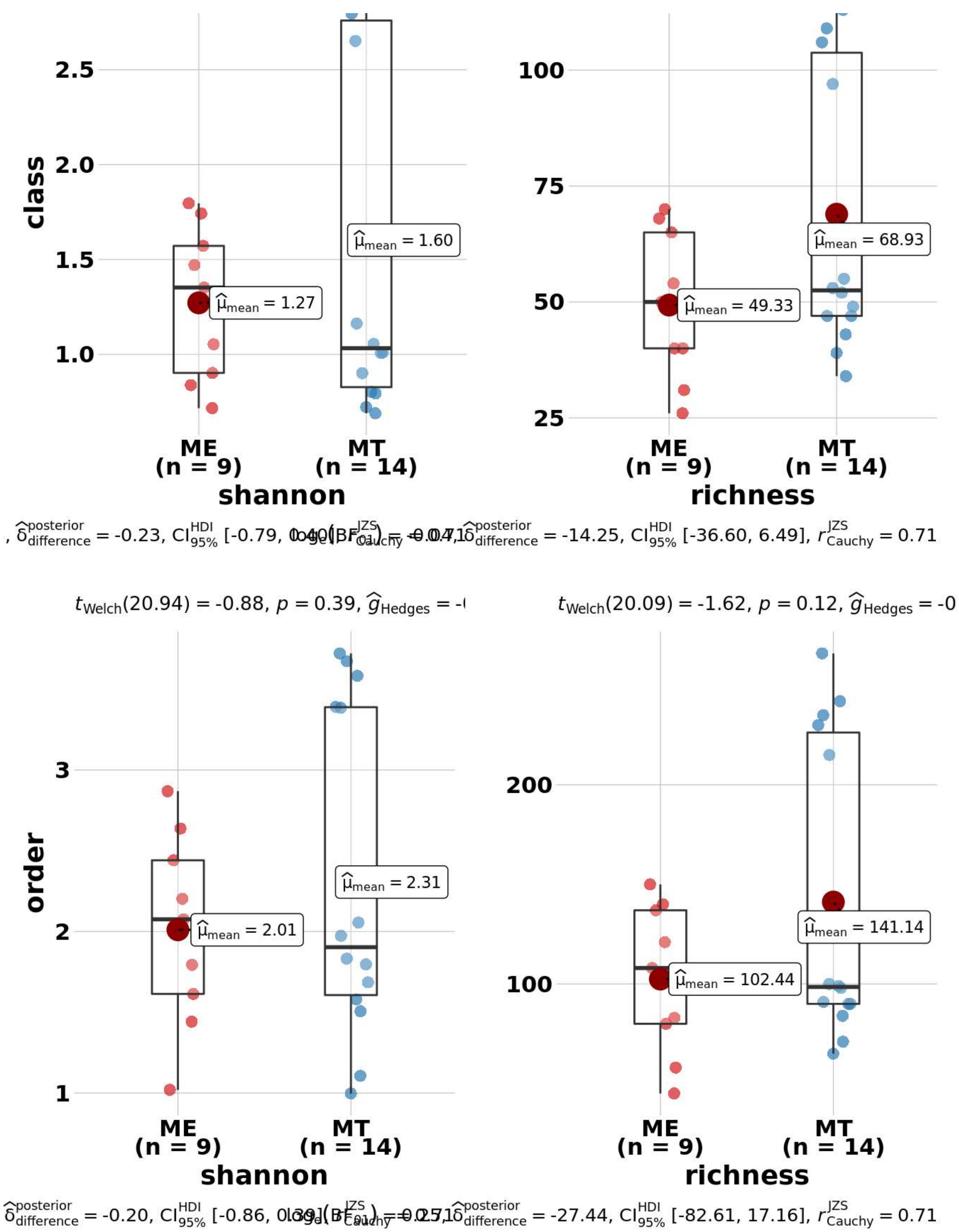

class
2.5
2.0
1.5
1.0
$\hat{\mu}_{mean}$ = 1.27
$\hat{\mu}_{mean}$ = 1.60
ME (n = 9)
MT (n = 14)
shannon
100
75
50
25
$\hat{\mu}_{mean}$ = 49.33
$\hat{\mu}_{mean}$ = 68.93
ME (n = 9)
MT (n = 14)
richness
$\hat{\delta}_{difference}^{posterior}$ = -0.23, $CI_{95\%}^{HDI}$ [-0.79, 0.40]
$\hat{\delta}_{difference}^{posterior}$ = -14.25, $CI_{95\%}^{HDI}$ [-36.60, 6.49], r_{Cauchy}^{JZS} = 0.71
t_{Welch}(20.94) = -0.88, p = 0.39, $\hat{g}_{Hedges}$ = -
t_{Welch}(20.09) = -1.62, p = 0.12, $\hat{g}_{Hedges}$ = -0
order
3
2
1
$\hat{\mu}_{mean}$ = 2.01
$\hat{\mu}_{mean}$ = 2.31
ME (n = 9)
MT (n = 14)
shannon
200
100
$\hat{\mu}_{mean}$ = 102.44
$\hat{\mu}_{mean}$ = 141.14
ME (n = 9)
MT (n = 14)
richness
$\hat{\delta}_{difference}^{posterior}$ = -0.20, $CI_{95\%}^{HDI}$ [-0.86, 0.39]
$\hat{\delta}_{difference}^{posterior}$ = -27.44, $CI_{95\%}^{HDI}$ [-82.61, 17.16], r_{Cauchy}^{JZS} = 0.71

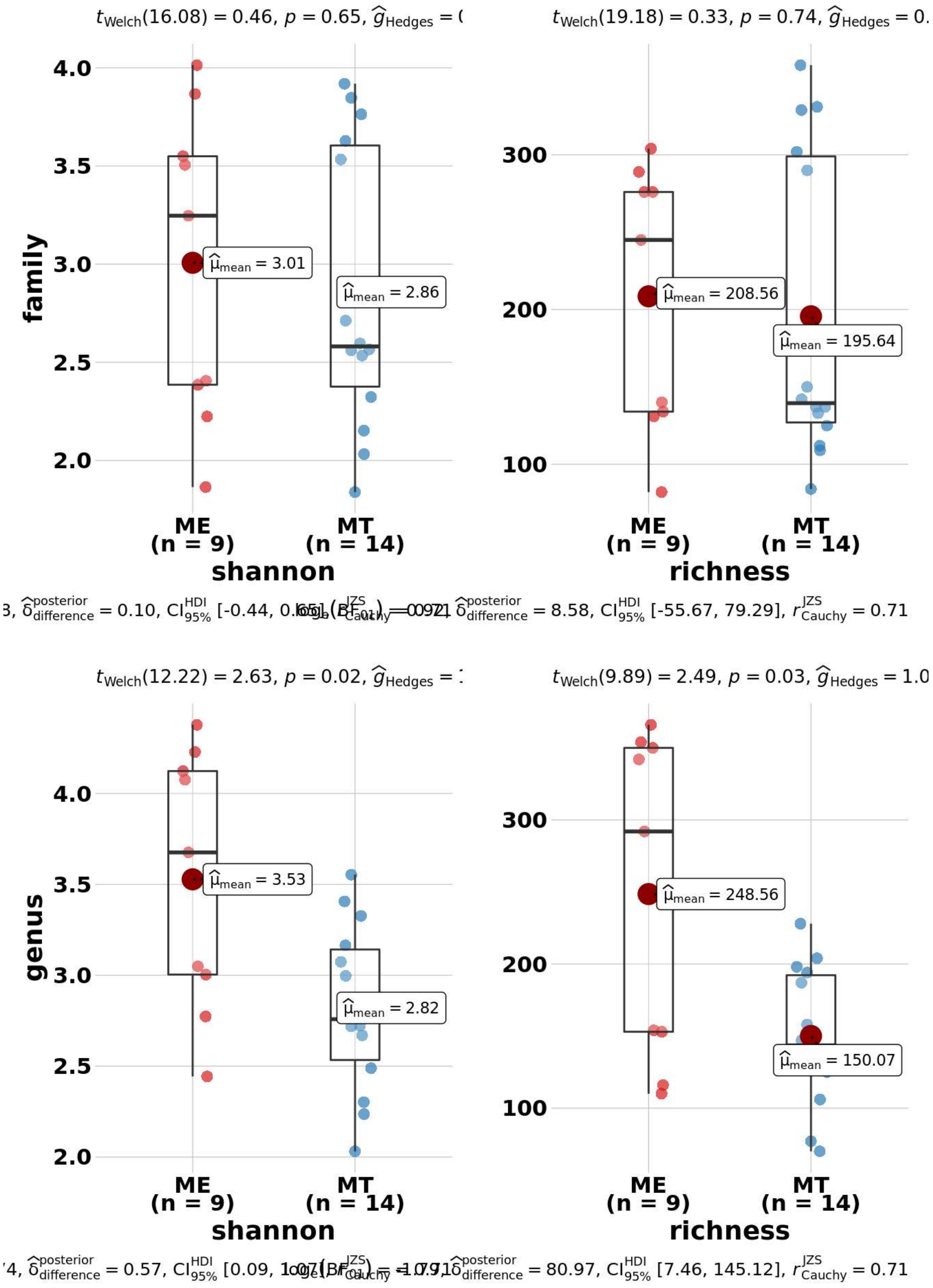

t_Welch(16.08) = 0.46, p = 0.65, ĝ_Hedges = (
t_Welch(19.18) = 0.33, p = 0.74, ĝ_Hedges = 0.
family
4.0
3.5
3.0
2.5
2.0
μ̂_mean = 3.01
μ̂_mean = 2.86
300
200
100
μ̂_mean = 208.56
μ̂_mean = 195.64
ME
(n = 9)
MT
(n = 14)
shannon
richness
δ̂_difference^posterior = 0.10, CI_95%^HDI [-0.44, 0.
δ̂_difference^posterior = 8.58, CI_95%^HDI [-55.67, 79.29], r_Cauchy^JZS = 0.71
t_Welch(12.22) = 2.63, p = 0.02, ĝ_Hedges =
t_Welch(9.89) = 2.49, p = 0.03, ĝ_Hedges = 1.0
genus
4.0
3.5
3.0
2.5
2.0
μ̂_mean = 3.53
μ̂_mean = 2.82
300
200
100
μ̂_mean = 248.56
μ̂_mean = 150.07
ME
(n = 9)
MT
(n = 14)
shannon
richness
δ̂_difference^posterior = 0.57, CI_95%^HDI [0.09, 1.
δ̂_difference^posterior = 80.97, CI_95%^HDI [7.46, 145.12], r_Cauchy^JZS = 0.71

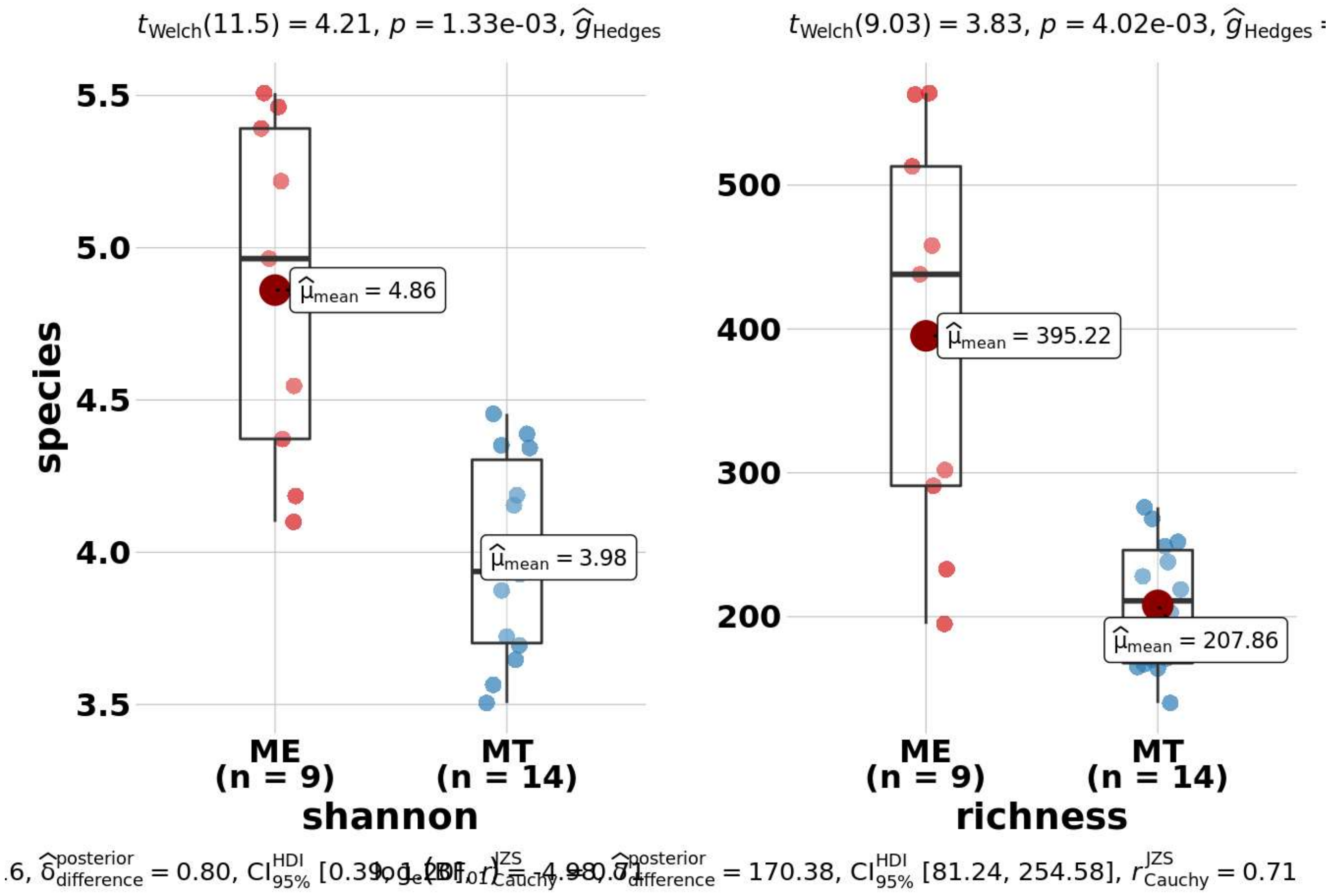


```
# p_all + plot_layout(ncol = 1)
```

The richness index of the prokaryotic community of ME was significantly lower than that in MT samples at the phylum level, while the opposite pattern was observed at the species level (Welch's t test; p value < 0.05).

Dissimilarity between the two extreme environments

```
p_all <- NULL
for (taxon.level.spec in taxon.levels[1:7]) {
  div.otu = collapse.div_to_taxon(div.phyloflash.rarefy, taxon.level.spec)

  p1 = plot.beta.div(div.otu, pname = taxon.level.spec,
                     method = "nmds", dist = "bray",
                     area = "ellipse") %>%
    p_theme %>%
    {. + guides(fill = "none", color = "none")}
  p1$labels$title =
    p1$labels$title %>% strsplit(., "\n") %>% unlist %>% {.[2]}
  p1$labels$x = "Bray-Curtis Distance"
  p1$labels$y = taxon.level.spec

  p2 = plot.beta.div(div.otu, pname = taxon.level.spec,
                    method = "nmds", dist = "jaccard",
                    area = "ellipse") %>%
    p_theme %>%
    {. + guides(fill = "none", color = "none")}
  p2$labels$title =
    p2$labels$title %>% strsplit(., "\n") %>% unlist %>% {.[2]}
  p2$labels$x = "Binary Jaccard Distance"
  p2$labels$y = ""
  print(p1 + p2)

  p1$labels$x = ""
  p2$labels$x = ""
  if (is.null(p_all)) {p_all = p1 + p2} else {p_all = p_all + p1 + p2}
}
```

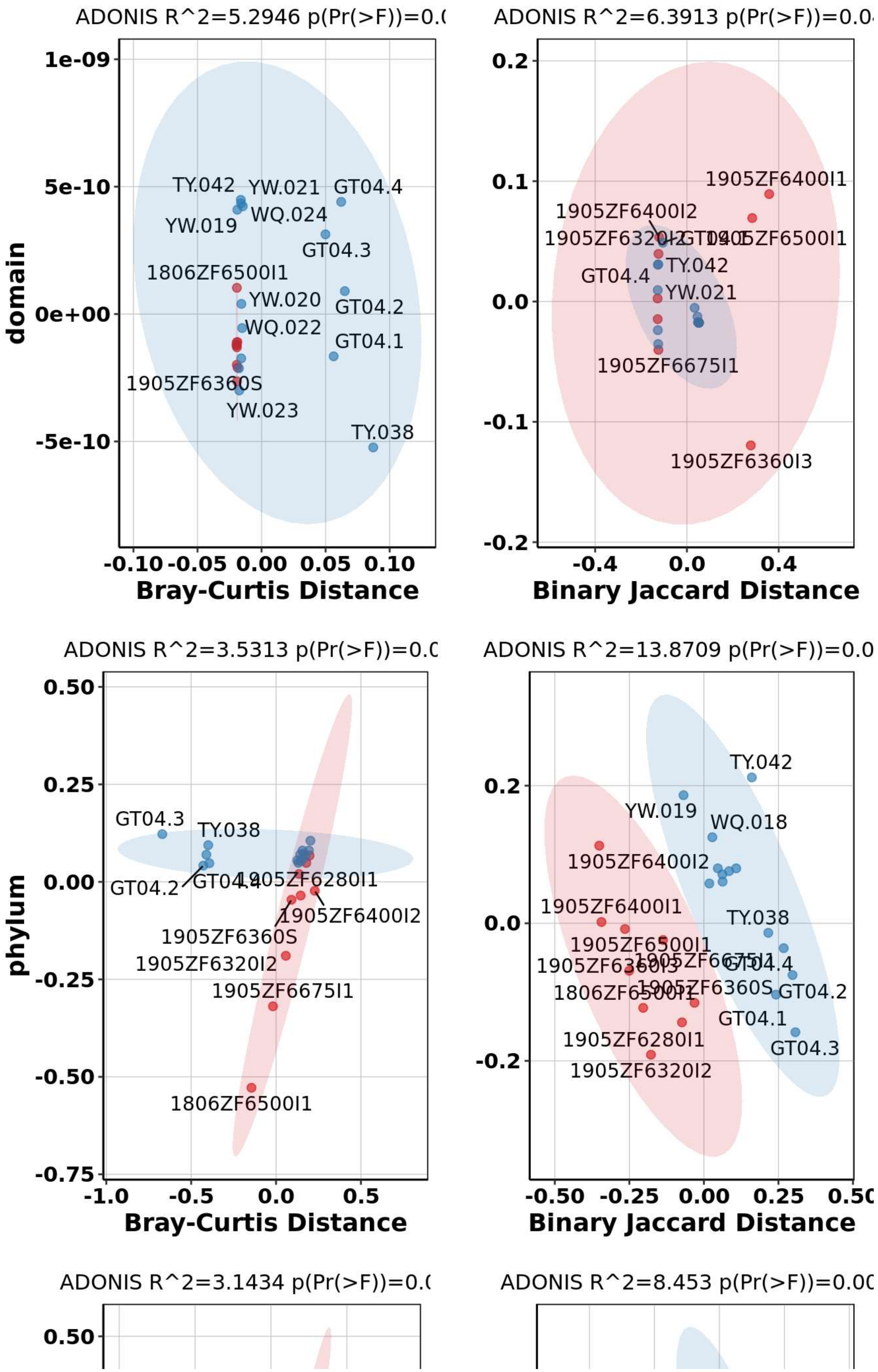
ADONIS R^2=5.2946 p(Pr(>F))=0.(
ADONIS R^2=6.3913 p(Pr(>F))=0.0
domain
1e-09
5e-10
0e+00
-5e-10
TY.042
YW.021
GT04.4
WQ.024
YW.019
GT04.3
1806ZF6500I1
YW.020
GT04.2
WQ.022
GT04.1
1905ZF6360S
YW.023
TY.038
-0.10
-0.05
0.00
0.05
0.10
Bray-Curtis Distance
0.2
0.1
0.0
-0.1
-0.2
1905ZF6400I1
1905ZF6400I2
1905ZF6500I1
TY.042
GT04.4
YW.021
1905ZF6675I1
1905ZF6360I3
-0.4
0.0
0.4
Binary Jaccard Distance
ADONIS R^2=3.5313 p(Pr(>F))=0.0
ADONIS R^2=13.8709 p(Pr(>F))=0.0
phylum
0.50
0.25
0.00
-0.25
-0.50
-0.75
GT04.3
TY.038
GT04.2
GT04.4
1905ZF6280I1
1905ZF6400I2
1905ZF6360S
1905ZF6320I2
1905ZF6675I1
1806ZF6500I1
-1.0
-0.5
0.0
0.5
Bray-Curtis Distance
0.2
0.0
-0.2
TY.042
YW.019
WQ.018
1905ZF6400I2
1905ZF6400I1
TY.038
1905ZF6500I1
GT04.4
1905ZF6360S
GT04.2
1806ZF6500I1
GT04.1
1905ZF6280I1
GT04.3
1905ZF6320I2
-0.50
-0.25
0.00
0.25
Binary Jaccard Distance
ADONIS R^2=3.1434 p(Pr(>F))=0.(
ADONIS R^2=8.453 p(Pr(>F))=0.00
0.50

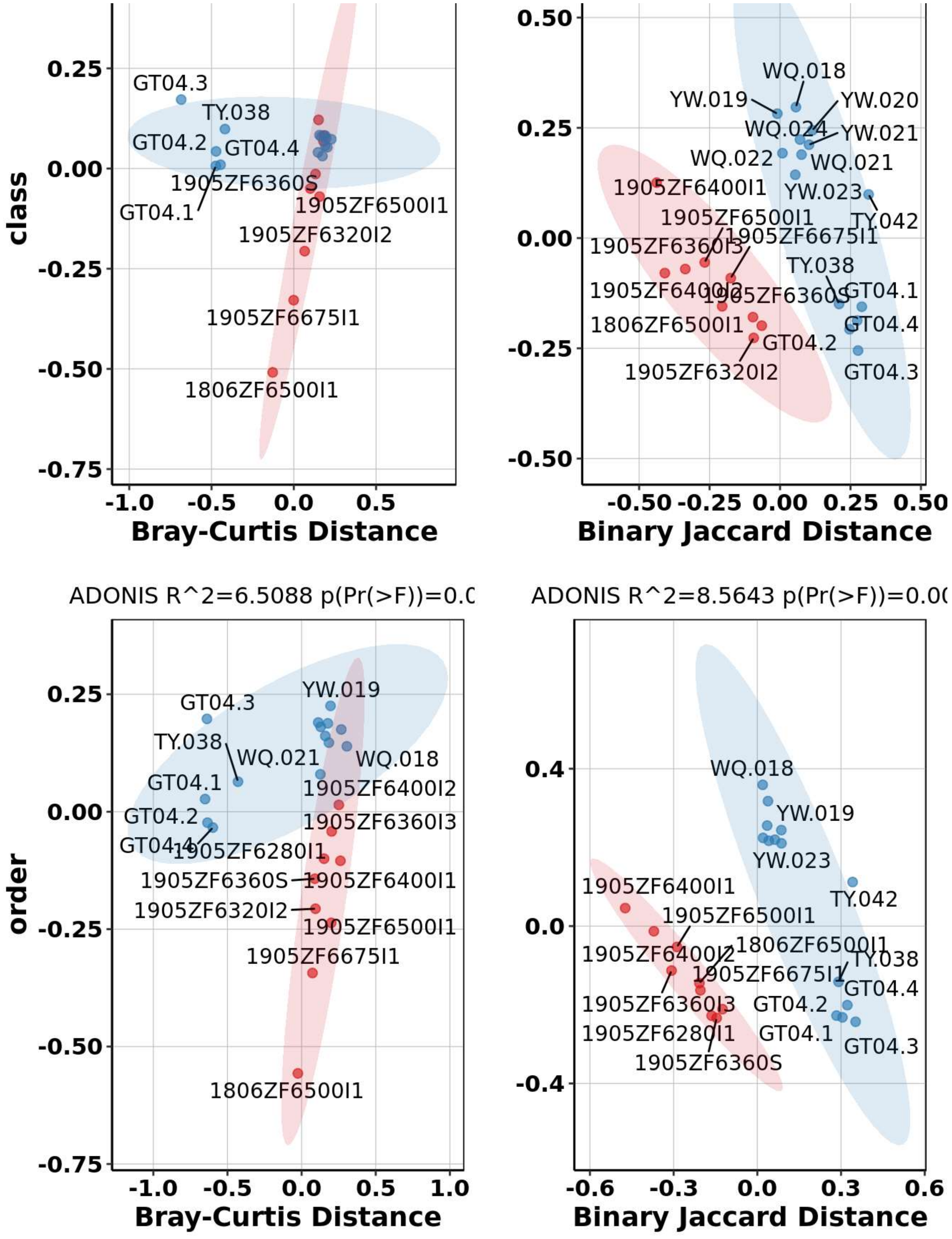

class
Bray-Curtis Distance
Binary Jaccard Distance
ADONIS R^2=6.5088 p(Pr(>F))=0.0
ADONIS R^2=8.5643 p(Pr(>F))=0.00
order
Bray-Curtis Distance
Binary Jaccard Distance
GT04.3
TY.038
GT04.2
GT04.4
GT04.1
1905ZF6360S
1905ZF6500I1
1905ZF6320I2
1905ZF6675I1
1806ZF6500I1
WQ.018
YW.019
YW.020
WQ.024
YW.021
WQ.022
WQ.021
YW.023
TY.042
1905ZF6400I1
1905ZF6360I3
1905ZF6400I2
1905ZF6280I1

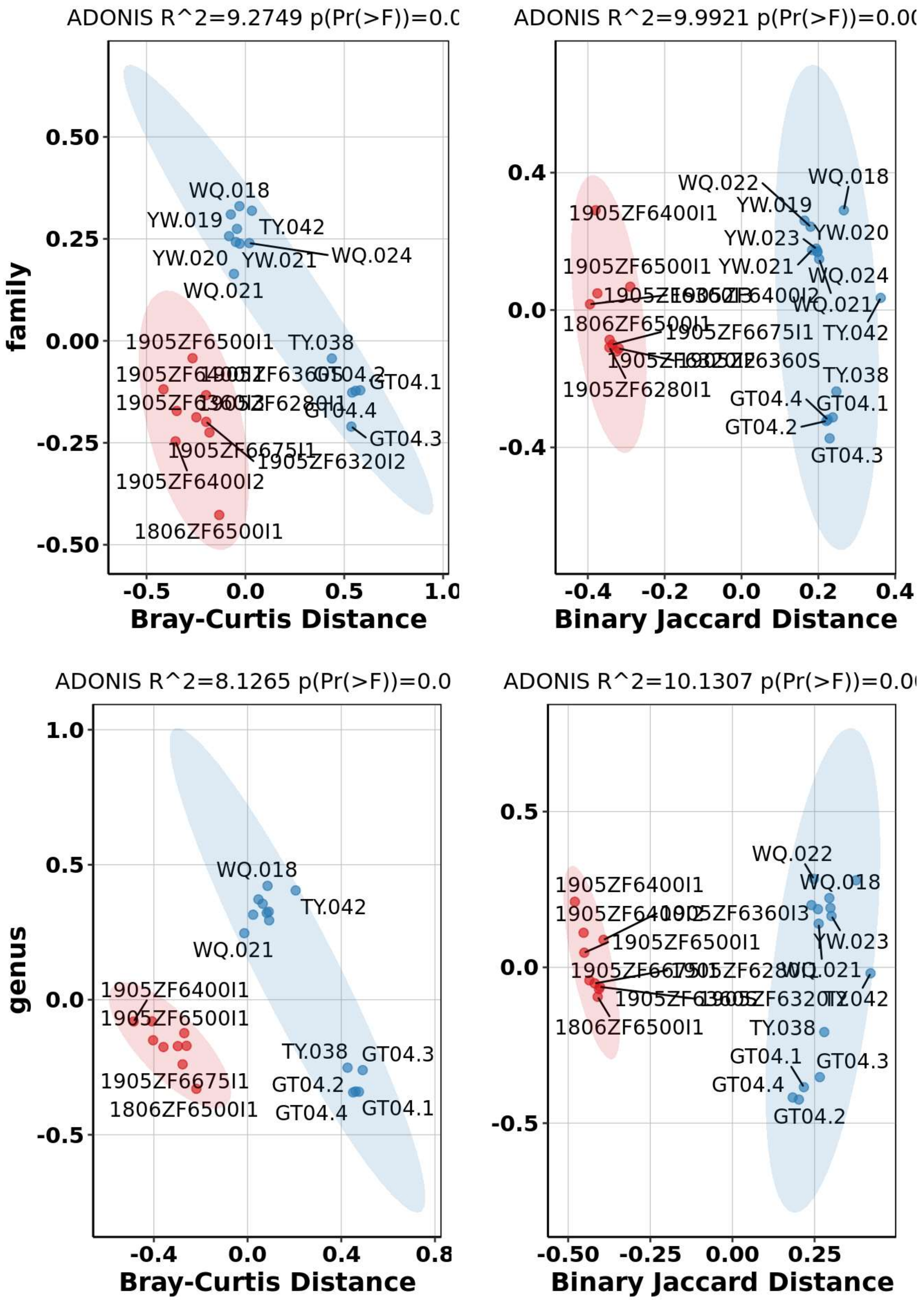

ADONIS R^2=9.2749 p(Pr(>F))=0.0
ADONIS R^2=9.9921 p(Pr(>F))=0.00
ADONIS R^2=8.1265 p(Pr(>F))=0.0
ADONIS R^2=10.1307 p(Pr(>F))=0.00
family
genus
Bray-Curtis Distance
Binary Jaccard Distance
0.50
0.25
0.00
-0.25
-0.50
-0.5
0.0
0.5
1.0
0.4
0.0
-0.4
-0.2
0.2
0.5
0.8
-0.50
-0.25
0.25
WQ.018
YW.019
TY.042
YW.020
YW.021
WQ.024
WQ.021
WQ.022
YW.023
TY.038
GT04.1
GT04.2
GT04.3
GT04.4
1905ZF6500I1
1905ZF6400I1
1905ZF6400I2
1905ZF6675I1
1905ZF6320I2
1905ZF6280I1
1905ZF6360I3
1806ZF6500I1

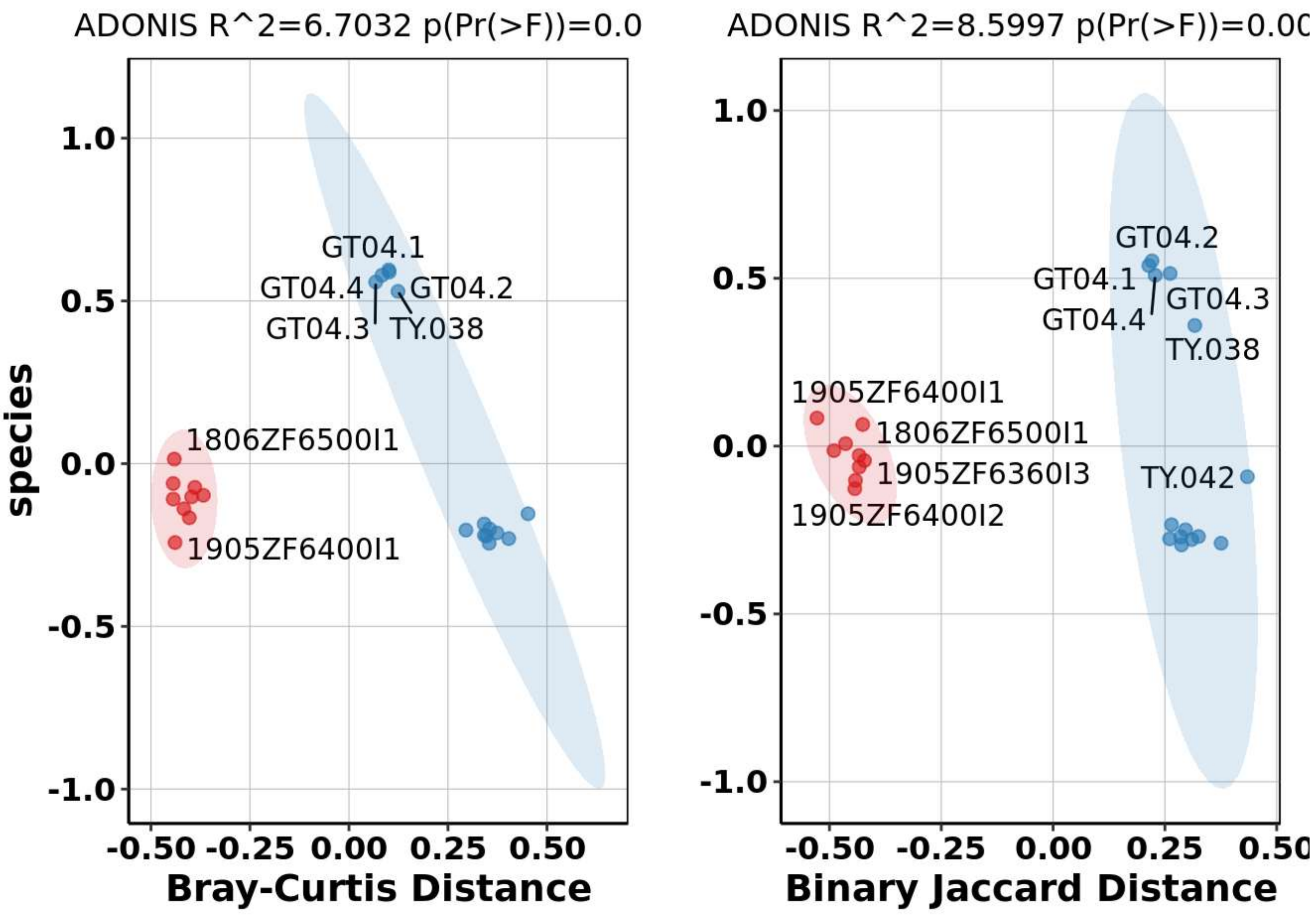


```
div.otu = ko_tpm %>% t %>% as.data.frame
p1 = plot.beta.div(div.otu, pname = "ko",
                   method = "nmds", dist = "bray",
                   area = "ellipse") %>%
  p_theme %>%
  {. + guides(fill = "none", color = "none")}
p1$labels$title = p1$labels$title %>% strsplit(., "\n") %>% unlist %>% {.[2]}
p1$labels$x = "Bray-Curtis Distance"
p1$labels$y = "ko"

p2 = plot.beta.div(div.otu, pname = taxon.level.spec,
                   method = "nmds", dist = "jaccard",
                   area = "ellipse") %>%
  p_theme %>%
  {. + guides(fill = "none", color = "none")}
p2$labels$title = p$labels$title %>% strsplit(., "\n") %>% unlist %>% {.[2]}
p2$labels$x = "Binary Jaccard Distance"
p2$labels$y = ""

print(p1 + p2)
```

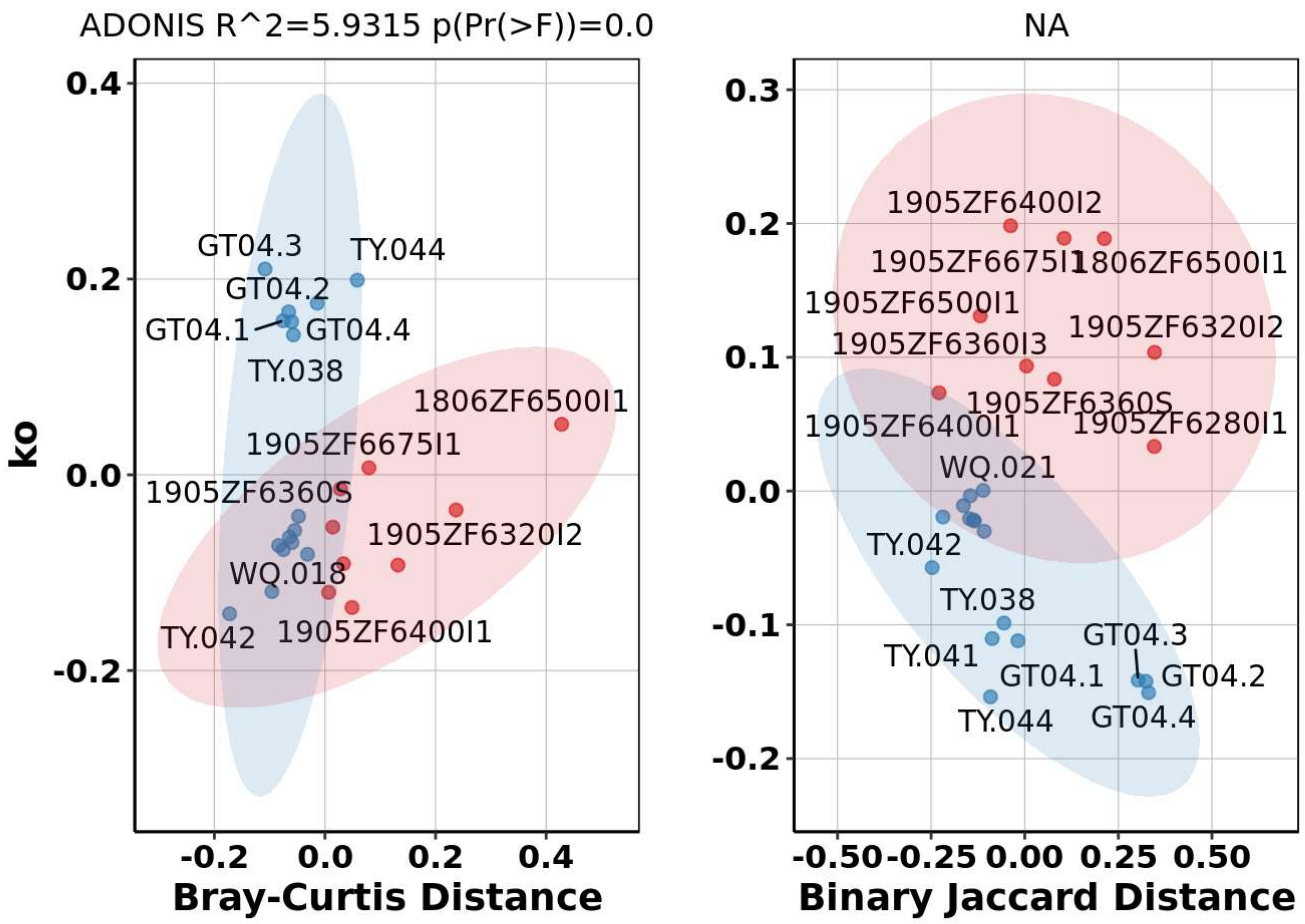


```
p_all = p_all + p1 + p2

#p_all + plot_layout(ncol = 2)
```

This dissimilarity between the two extreme environments significantly differed at levels below class (Adonis test; p value < 0.05), regardless of the presence (binary Jaccard distance) or abundance (Bary-Cruit distance) of taxa.

```
dist.gene =
  lapply(list(ko = "ko_tpm", module = "module_tpm"),
         function(x) {
           value.name = gsub("(\\w+)_tpm", "\\1", x)
           get(x) %>%
             {vegdist(., method = 'bray')} %>%
             {reshape2::melt(as.matrix(.), value.name = value.name)}
         }) %>%
  {merge(.$ko, .$module)}

dist.div.collapse <- function(x) {
  div.phyloflash.rarefy %>%
    {collapse.div_to_taxon(div.raw = ., taxon.level.spec = x)} %>%
    {t(.)} %>%
    {vegdist(., method = 'bray')} %>%
    {reshape2::melt(as.matrix(.), value.name = x)}
}

dist.genetaxons =
  taxon.levels %>%
  {as.character(.)} %>%
  {lapply(., dist.div.collapse)} %>%
  {Reduce(f = merge, x = ., init = dist.gene)} %>%
  {.[as.integer(factor(.$Var1)) < as.integer(factor(.$Var2)),]} %>%
  {
    assign_env = function(x) {
      x %>%
        {as.character(.)} %>%
        {ifelse(grepl("^ME", .), "ME", "MT")}
    }
    .$Var1.env = .$Var1 %>% assign_env
    .$Var2.env = .$Var2 %>% assign_env
    .
  } %>% {
    .$comparisons =
      ifelse(.$Var1.env == .$Var2.env, .$Var1.env, "ME vs MT") %>%
      {factor(., levels = c("ME", "MT", "ME vs MT"))}
    .
  }
dist.kotaxons.long =
  melt(dist.genetaxons,
       id.vars = c("comparisons"),
       measure.vars = c(taxon.levels %>% as.character,
                        c("module", "ko")),
       variable.name = "method", value.name = "distance") %>%
  {levels(.$method) = c(taxon.levels %>% {stringr::str_to_title(.)},
                        "module", "KO"); .}


p =
  ggplot(data = dist.kotaxons.long) +
  geom_boxplot(mapping = aes_string(x = "comparisons", y = "distance",
                                    fill = "comparisons")) +
  scale_x_discrete(label = NULL) +
  theme(axis.ticks.length = unit(0, 'cm')) +
  scale_fill_manual(values = c(c("ME" = "#d7191c",
```

```
                                    "MT" = "#2c7bb6",
                                    "ME vs MT" = "#984ea3"))) +
  geom_signif(mapping = aes_string(x = "comparisons", y = "distance"),
              comparisons = combn(c("ME", "MT", "ME vs MT"), 2, FUN = list),
              map_signif_level = TRUE,
              step_increase = 0.07, tip_length = 0.01) +
  facet_grid(rows = formula("~ method"))

p
```

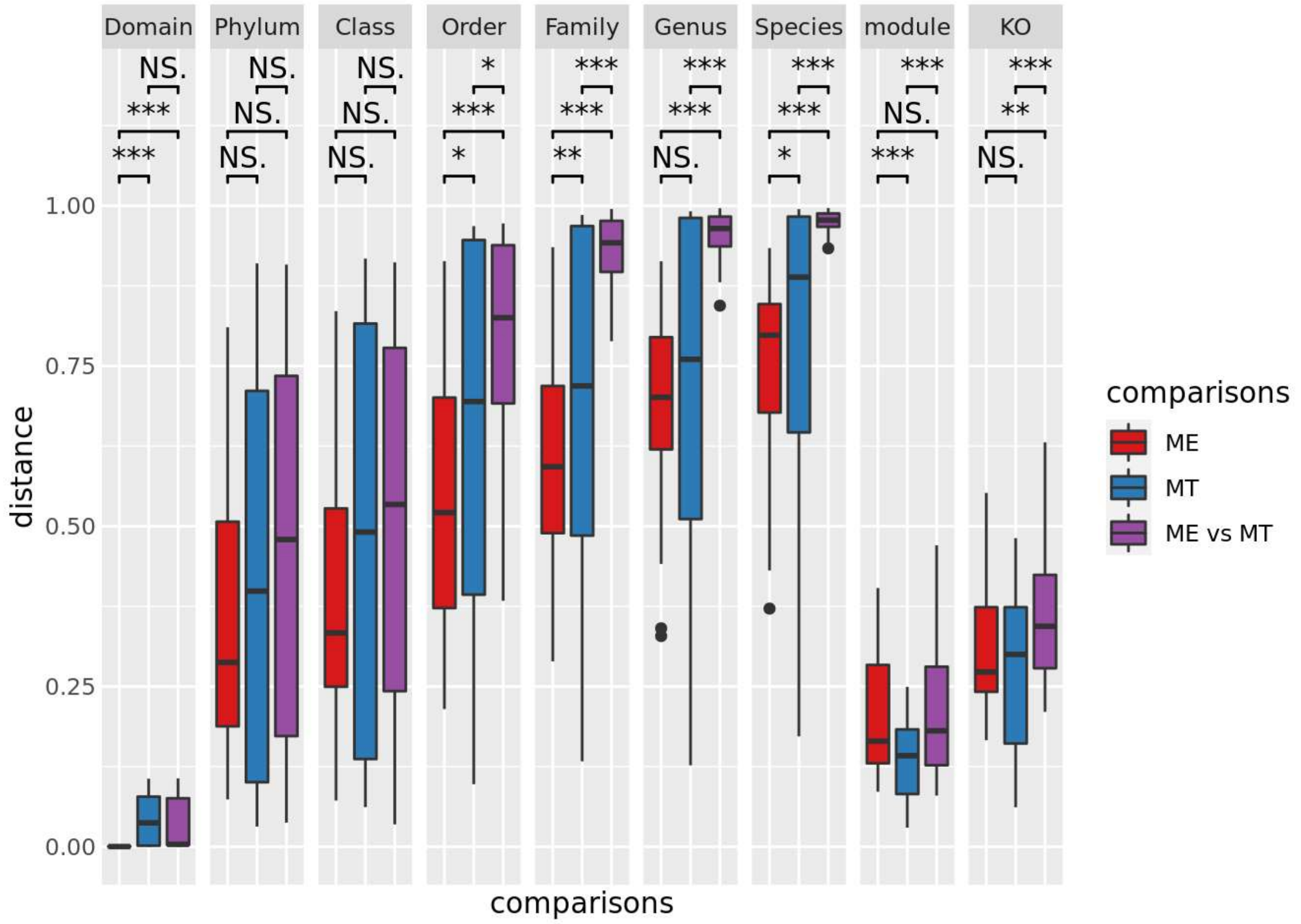

This dissimilarity between the two extreme environments increased from the domain to species level.

Metagenomic assemblied genomes

Taxonomy and function prevalence across environments

```
genomeko_long =
  genomeko %>%
  .[Wtdb$genome, ] %>%
  data.frame(., genome = Wtdb$genome, taxa = Wtdb$classification) %>%
  pivot_longer(!c("genome", "taxa"),
               names_to = "ko",
               values_to = "count") %>%
  filter(count > 0) %>%
  {.$location = grepl("^1", .$genome) %>% ifelse("ME", "MT"); .} %>%
  {.$taxa = .$taxa %>% str_split_fixed(";", 7) %>% .[, 3]; .}

ko2module =
  merge(read.csv("workflow/gene_annot/module_name.tsv", sep = "\t"),
        read.csv("workflow/gene_annot/entry2ko.csv")) %>%
  {data.frame(ko = .$kos, pathway = .$C)}

df =
  genomeko_long %>%
  left_join(ko2module, by = "ko") %>%
  group_by(location, taxa, pathway) %>%
  dplyr::summarise(count = sum(count)) %>%
  filter(!is.na(pathway)) %>%
  filter(taxa != "c__") %>%
  arrange(desc(count))

library(igraph)
library(networkD3)
library(htmlwidgets)

d3 =
  df %>%
  {
    rbind(
      data.frame(count = .$count, type = .$location,
                 source = .$taxa, target = .$location),
      data.frame(count = .$count, type = .$location,
                 source = .$location, target = .$pathway)
    )
  } %>%
  group_by(source, target) %>%
  summarise(count = sum(count)) %>%

  graph_from_data_frame %>%
  igraph_to_networkD3

d3$links$type <-
  ifelse(
    d3$nodes[d3$links$source + 1, "name"] %in% c("ME", "MT"),
    d3$nodes[d3$links$source + 1, "name"],
    ifelse(d3$nodes[d3$links$target + 1, "name"] %in% c("ME", "MT"),
           d3$nodes[d3$links$target + 1, "name"],
           NA)
  )
d3$nodes$type <- ifelse(
  d3$nodes$name %in% c("ME", "MT"),
```

```
  d3$nodes$name,
  NA)
# color the ME or MT specific taxa and function as well
source_count <- table(d3$links$source)
target_count <- table(d3$links$target)
index <- which(d3$links$source %in% as.numeric(names(source_count)[source_count == 1]))
d3$nodes$type[d3$links$source[index] + 1] <- d3$links$type[index]
index <- which(d3$links$source %in% as.numeric(names(source_count)[source_count == 2]))
d3$nodes$type[d3$links$source[index] + 1] <- "cross"
index <- which(d3$links$target %in% as.numeric(names(target_count)[target_count == 1]))
d3$nodes$type[d3$links$target[index] + 1] <- d3$links$type[index]
index <- which(d3$links$target %in% as.numeric(names(target_count)[target_count == 2]))
d3$nodes$type[d3$links$target[index] + 1] <- "cross"


my_color <- 'd3.scaleOrdinal() .domain(["ME", "MT", "cross"]) .range(["#d7191c", "#2c7bb6", "#9
84ea3"])'
```

```
sn <- sankeyNetwork(d3$links, d3$nodes, Source = "source", Value = "value",
              Target = "target", LinkGroup = "type", fontSize = 0,
              nodePadding = 2,
              nodeWidth = 100,
              NodeGroup = "type",
              colourScale = my_color)

sn
```

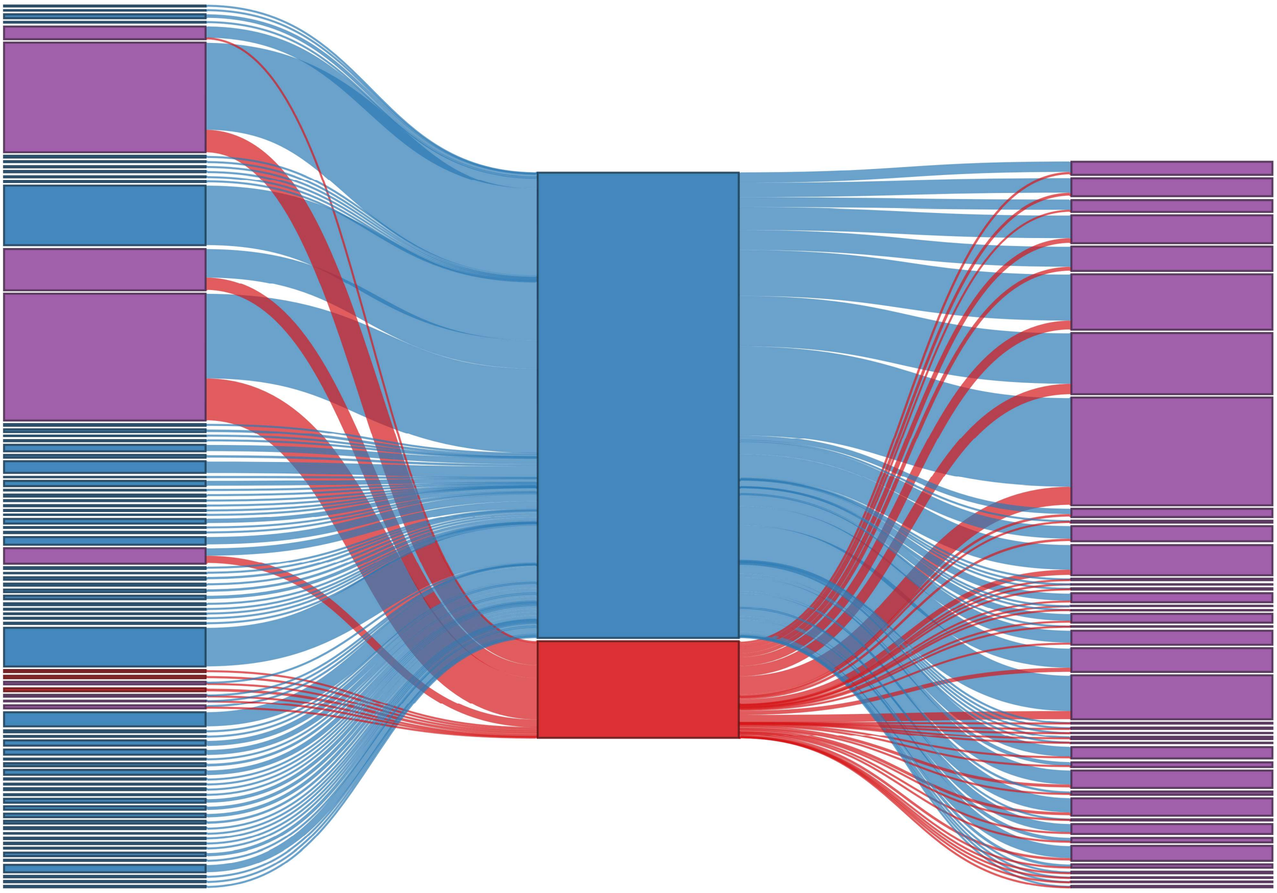

The MAG-based Sankey network also revealed the distinct prokaryotic taxa between ME and MT.

The 648 representative MAGs were distributed into 47 phyla.

Regardless of ME or MT, most MAGs belonged to:

```
MAG_info$classification %>% taxon.split(2) %>% table %>% sort(decreasing = TRUE) %>% head(7)
```

```
## .
##   Proteobacteria    Chloroflexota  Planctomycetota     Bacteroidota
##              449              119              113              112
## Actinobacteriota   Marinisomatota  Gemmatimonadota
##               71               37               27
```

Shared taxa between ME and MT

```
gmodule =
  genomeko %>%
  {reshape2::melt(., varnames = c("sample", "ko"), value.name = "tpm")} %>%
  {
    ko2entry =
      read.csv("data/entry2ko.csv") %>%
      unique(.) %>%
      {.[.$kos %in% colnames(genomeko),]}
    dplyr::full_join(x = ., y = ko2entry,
                     by = c("ko" = "kos"))
  } %>%
  (function(x) {x["entry"][is.na(x["entry"])] = ""; x})(.) %>%
  {reshape2::acast(., formula = formula("sample ~ entry"),
                   fun.aggregate = sum, value.var = "tpm")} %>%
  {subset(., select = c(-1))}

taxon.split.filtannot <- function(taxon.level.spec, env.spec) {
  MAG_info %>%
    {.[c("classification", "location")]} %>%
    {unique(.)} %>%
    {.$classification[.$location == env.spec]} %>%
    {taxon.split(., 1, taxon.level.spec)} %>%
    {gsub("(;)$", str_glue(";{env.spec}"), .)}
}
```

```
library(VennDiagram)
p_all <- NULL
for (taxon.level.spec in taxon.levels) {
  venn.grid =
    list(ME = taxon.split.filtannot(taxon.level.spec, "ME"),
         MT = taxon.split.filtannot(taxon.level.spec, "MT")) %>%
    {
      venn.diagram(
        x = .,
        filename = NULL, imagetype = "png",
        fill = c(ME = "#d7191c", MT = "#2c7bb6"), alpha = 0.75,
        label.col = "black",
        fontfamily = "Arial",
        main = taxon.level.spec, main.fontfamily = "Arial",
        main.pos = c(0.5, 0), main.just = c(0.5, 1),
        cat.col = c(ME = NA, MT = NA),
        ext.line.lty = "dotted", ext.dist = -0.1,
        disable.logging = FALSE
      )
    } %>%
    as_ggplot %>%
    {. + theme(plot.margin = unit(rep(0.3, 4), "in"))}
  if (is.null(p_all)) {
    p_all = venn.grid
  } else {
    p_all = p_all + venn.grid
  }
}

venn.grid.gene = function(x, pname) {
  MAG_info %>%
    {.[c("genome", "location")]} %>%
    {
      split(x[.$genome, ] %>% data.frame, .$location)
    } %>%
    {lapply(., function(x) apply(x, 2, max))} %>%
    {lapply(., function(x) names(x)[x > 0])} %>%

    {list(ME = .$ME, MT = .$MT)} %>%
    {
      venn.diagram(
        x = .,
        filename = NULL, imagetype = "png",
        fill = c(ME = "#d7191c", MT = "#2c7bb6"), alpha = 0.75,
        label.col = "black",
        fontfamily = "Arial",
        main = pname, main.fontfamily = "Arial",
        main.pos = c(0.5, 0), main.just = c(0.5, 1),
        cat.col = c(ME = NA, MT = NA),
        ext.line.lty = "dotted", ext.dist = -0.1,
        disable.logging = FALSE
      )
    } %>%
    as_ggplot %>%
    {. + theme(plot.margin = unit(rep(0.3, 4), "in"))}
```

```
}

p =
  p_all +
  venn.grid.gene(genomeko, "ko") +
  venn.grid.gene(gmodule, "module") +
  plot_layout(ncol = 3)

p
```

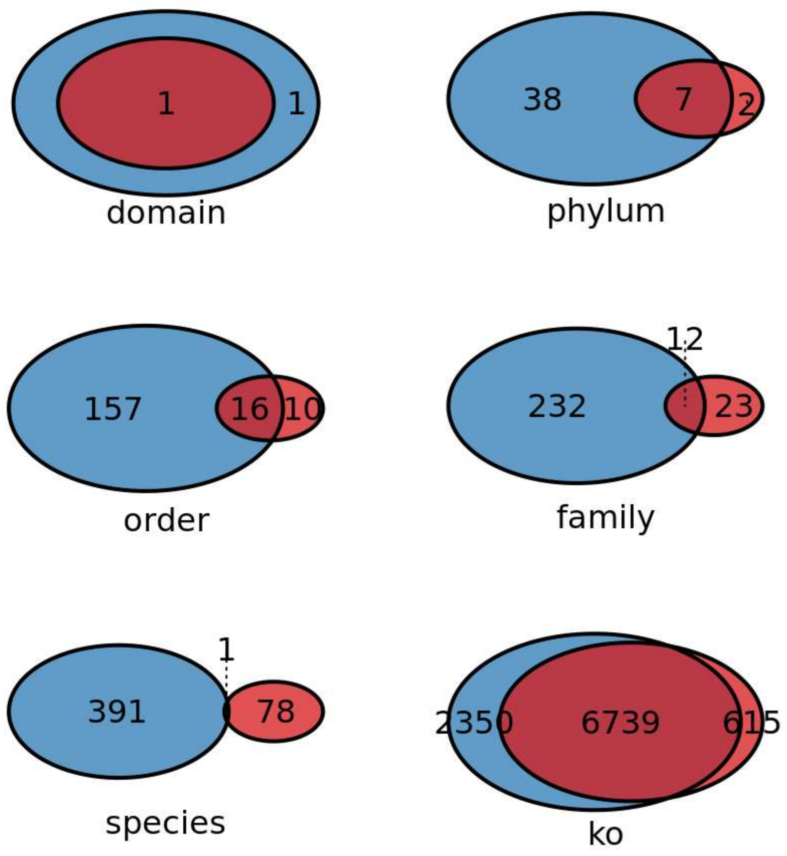

ME and MT shared a very small number of taxa, especially at a finer level (at or below the family)

Genera shared across ME and MT are listed here:

```
list(ME = taxon.split.filtannot("genus", "ME"),
     MT = taxon.split.filtannot("genus", "MT")) %>%
  {.$ME[.$ME %in% .$MT]} %>% sort %>% table
```

```
## .
##      Bacteria;Actinobacteriota;Actinomycetia;Propionibacteriales;Nocardioidaceae;Nocardioide
s
##
1
##           Bacteria;Bacteroidota;Bacteroidia;Sphingobacteriales;Sphingobacteriaceae;Pedobacte
r
##
1
##    Bacteria;Proteobacteria;Alphaproteobacteria;Caulobacterales;Caulobacteraceae;Brevundimona
s
##
2
## Bacteria;Proteobacteria;Alphaproteobacteria;Caulobacterales;Caulobacteraceae;Phenylobacteriu
m
##
1
##   Bacteria;Proteobacteria;Alphaproteobacteria;Sphingomonadales;Sphingomonadaceae;Sphingomona
s
##
3
##        Bacteria;Proteobacteria;Gammaproteobacteria;Burkholderiales;Burkholderiaceae;Comamona
s
##
2
##      Bacteria;Proteobacteria;Gammaproteobacteria;Pseudomonadales;Moraxellaceae;Acinetobacte
r
##
4
## Bacteria;Proteobacteria;Gammaproteobacteria;Xanthomonadales;Xanthomonadaceae;Stenotrophomona
s
##
1
```

Distribution of the best average nucleotide identity (ANI) of MAGs between ME and MT

```
closest_Mdb =
  read.csv("data/closest_Mdb.csv", header = FALSE) %>%
  {closest_Mdb = .$V2; names(closest_Mdb) = .$V1; closest_Mdb}

p = ggplot(data = data.frame(similarity = closest_Mdb / 100)) +
  geom_histogram(mapping = aes_string(x = "similarity"),
                 breaks = seq(0.72, 1, 0.004)) +
  labs(x = "highest similarity") +
  scale_x_continuous(breaks = seq(0.7, 1.0, 0.05),
                     labels = seq(0.7, 1.0, 0.05),
                     limits = c(0.7, 1.0)) +
  geom_vline(xintercept = 0.95, color = "#006b3c", linetype = "dashed") +
  geom_vline(xintercept = 0.80, color = "#8a3324", linetype = "dashed")

library(gg.gap)
pg = gg.gap(plot = p,
            segments = list(c(17, 30), c(250, 600)),
            tick_width = c(5, 100, 10),
            rel_heights = c(1, 0, 0.4, 0, 0.2),
            ylim = c(0, 615))
pg
```

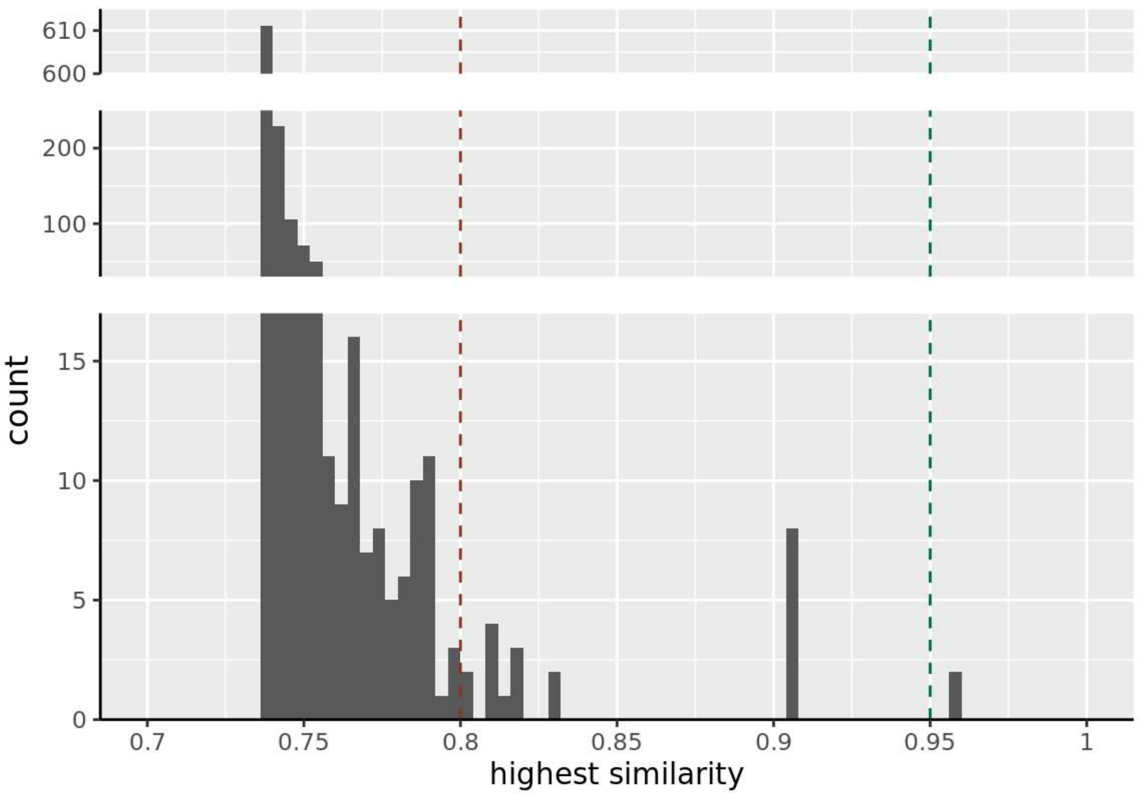

The ANIs between genomes from ME and MT were all less than 80%, lower than the commonly accepted within-family cutoff of genome similarity.

```
closest_Mdb %>%
  {merge(., MAG_info, by.x = 0, by.y = "genome")} %>%
  #{colnames(.)}
  {.[c("Row.names", "classification", "x")]} %>%
  {.[.$x %>% order %>% rev, ]} %>%
  {.[.$x >= 80, ]} %>%
  {
    .$classification = .$classification %>% taxon.split.last
    .
  } %>%
  {
    colnames(.) = c("genome", "classification.last", "ANI")
    .
  }
```

```
##                                genome           classification.last     ANI
## 838            WQ.021-megahit.128.fna        Comamonas tsuruhatensis 95.6823
## 85     1905ZF6400I1_P95S60_mtb2.31.fa        Comamonas tsuruhatensis 95.6823
## 960             WQ.024-megahit.20.fna      Nocardioides sp001627335 90.5374
## 45     1905ZF6360I3_P95S60_mtb2.28.fa          Nocardioides marinus 90.5374
## 841            WQ.021-megahit.130.fna      Nocardioides sp001627335 90.5202
## 1132           YW.023-megahit.120.fna      Nocardioides sp001627335 90.4816
## 980            YW.019-megahit.102.fna      Nocardioides sp001627335 90.4805
## 1126            YW.021-megahit.68.fna      Nocardioides sp001627335 90.4748
## 1013           YW.020-megahit.138.fna      Nocardioides sp001627335 90.4624
## 913             WQ.022-megahit.96.fna      Nocardioides sp001627335 90.4355
## 961             WQ.024-megahit.24.fna Stenotrophomonas maltophilia_P 82.8106
## 35     1905ZF6360I3_concoct_87_sub.fa Stenotrophomonas maltophilia_Z 82.8106
## 1084           YW.021-megahit.146.fna         Brevundimonas diminuta 81.7315
## 67      1905ZF6360S_P95S75_mtb2.22.fa   Brevundimonas mediterranea_A 81.7315
## 1022            YW.020-megahit.15.fna         Brevundimonas diminuta 81.7078
## 1173            YW.023-megahit.68.fna         Brevundimonas diminuta 81.4241
## 16     1905ZF6280I1_P75S60_mtb2.13.fa                    g__Comamonas 81.1387
## 27     1905ZF6320I2_P75S75_mtb2.10.fa                    g__Comamonas 81.1380
## 65   1905ZF6360S_P95S60_mtb2.2_sub.fa                    g__Comamonas 81.0419
## 96     1905ZF6400I2_concoct_27_sub.fa                    g__Comamonas 80.9819
## 117    1905ZF6500I1_P95S60_mtb2.40.fa                g__Brevundimonas 80.1510
## 95         1905ZF6400I2_concoct_17.fa             g__Pseudorhodoferax 80.1058
```

Comparison among the MAGs belonging to ME-specific, MT-specific and cross-habitat classes

```
MAG_info %>% {split(.$GenomeSize / 1e6, .$location)} %>% lapply(summary) %>% bind_rows(.id = "l
ocation") %>% print
```

```
## # A tibble: 2 × 7
##   location Min.     `1st Qu.` Median   Mean     `3rd Qu.` Max.
##   <chr>    <table>  <table>   <table>  <table>  <table>   <table>
## 1 ME       1.129140 2.856486  3.782225 3.885512 4.545737  9.186947
## 2 MT       0.416342 1.979515  2.785743 2.865953 3.605532  7.728039
```

```
MAG_info %>% {split(.$GC, .$location)} %>% lapply(summary) %>% bind_rows(.id = "location") %>%
  print
```

```
## # A tibble: 2 × 7
##   location Min.      `1st Qu.` Median    Mean      `3rd Qu.` Max.
##   <chr>    <table>   <table>   <table>   <table>   <table>   <table>
## 1 ME       0.3325085 0.4708852 0.6095361 0.5714295 0.6626890 0.7219540
## 2 MT       0.2648932 0.4736469 0.5927389 0.5560643 0.6406045 0.7253278
```

```
MAG_info %>% {split(.$OGT, .$location)} %>% lapply(summary) %>% bind_rows(.id = "location") %>%
print
```

```
## # A tibble: 2 × 7
##   location Min.    `1st Qu.` Median  Mean     `3rd Qu.` Max.
##   <chr>    <table> <table>   <table> <table>  <table>   <table>
## 1 ME       11      20        25      25.77692 29        45
## 2 MT       16      33        40      40.04876 46        77
```

```
pb = MAG_info %>% {.[, "G+C content (%)"] = .$GC; .} %>%
  ggsignif.local("location", "`G+C content (%)`", sample_meta_col)
pc = MAG_info %>% {.[, "Genome size (Mbp)"] = .$GenomeSize / 1e6; .} %>%
  ggsignif.local("location", "`Genome size (Mbp)`", sample_meta_col)
pd = MAG_info %>% {.[, "Optimal growth temperature (℃)"] = .$OGT; .} %>%
  ggsignif.local("location", "`Optimal growth temperature (℃)`", sample_meta_col)
print(pb + pc + pd)
```

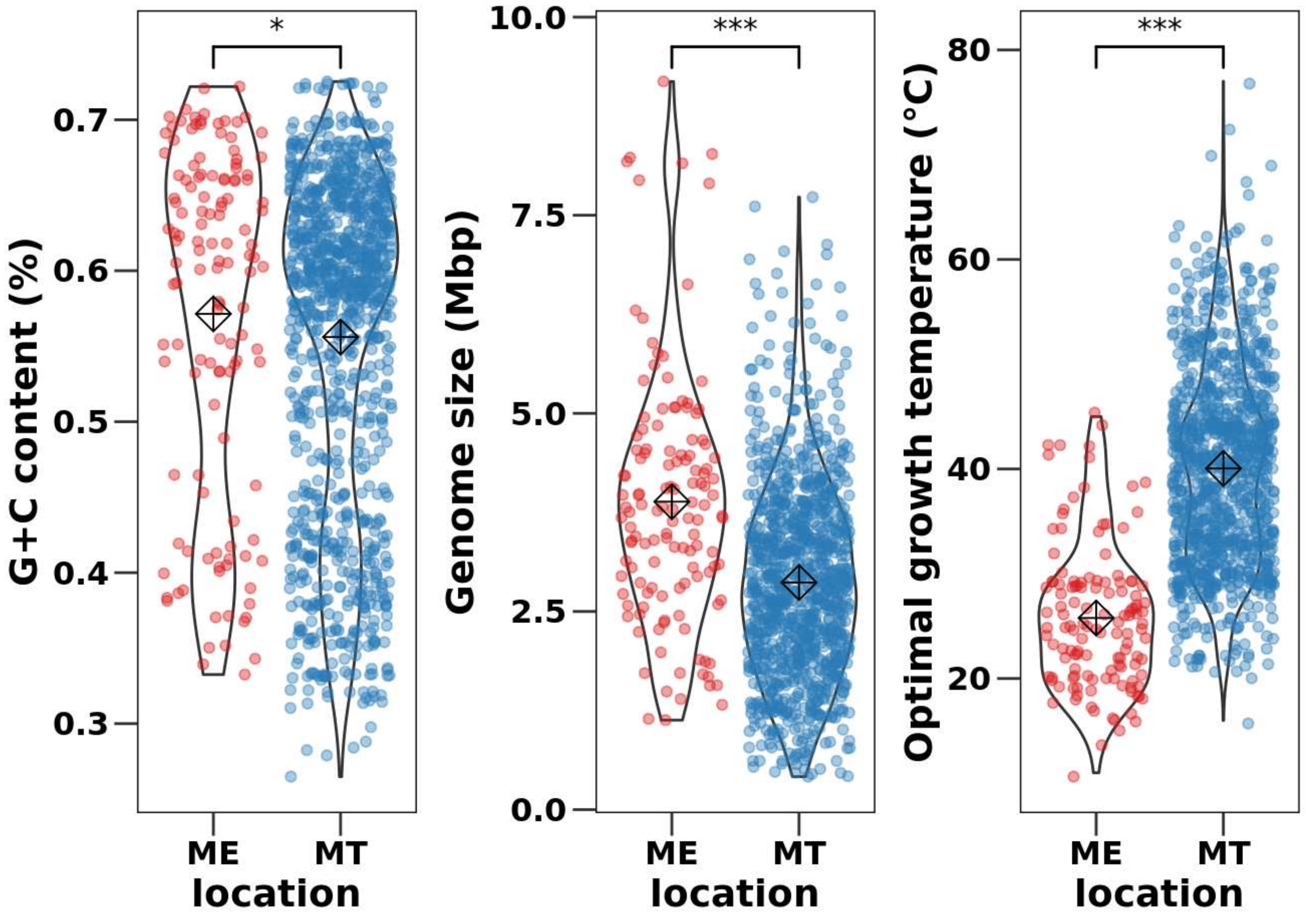

```
venn.class.color = c(
  "ME.only" = "#d7191c",
  "ME.both" = "#f28d8e",
  "MT.both" = "#98c5e6",
  "MT.only" = "#2c7bb6"
)

p = MAG_info %>% {.[, "G+C content (%)"] = .$GC; .} %>%
  ggbetweenstats.local2("G+C content (%)")
print(p)
```

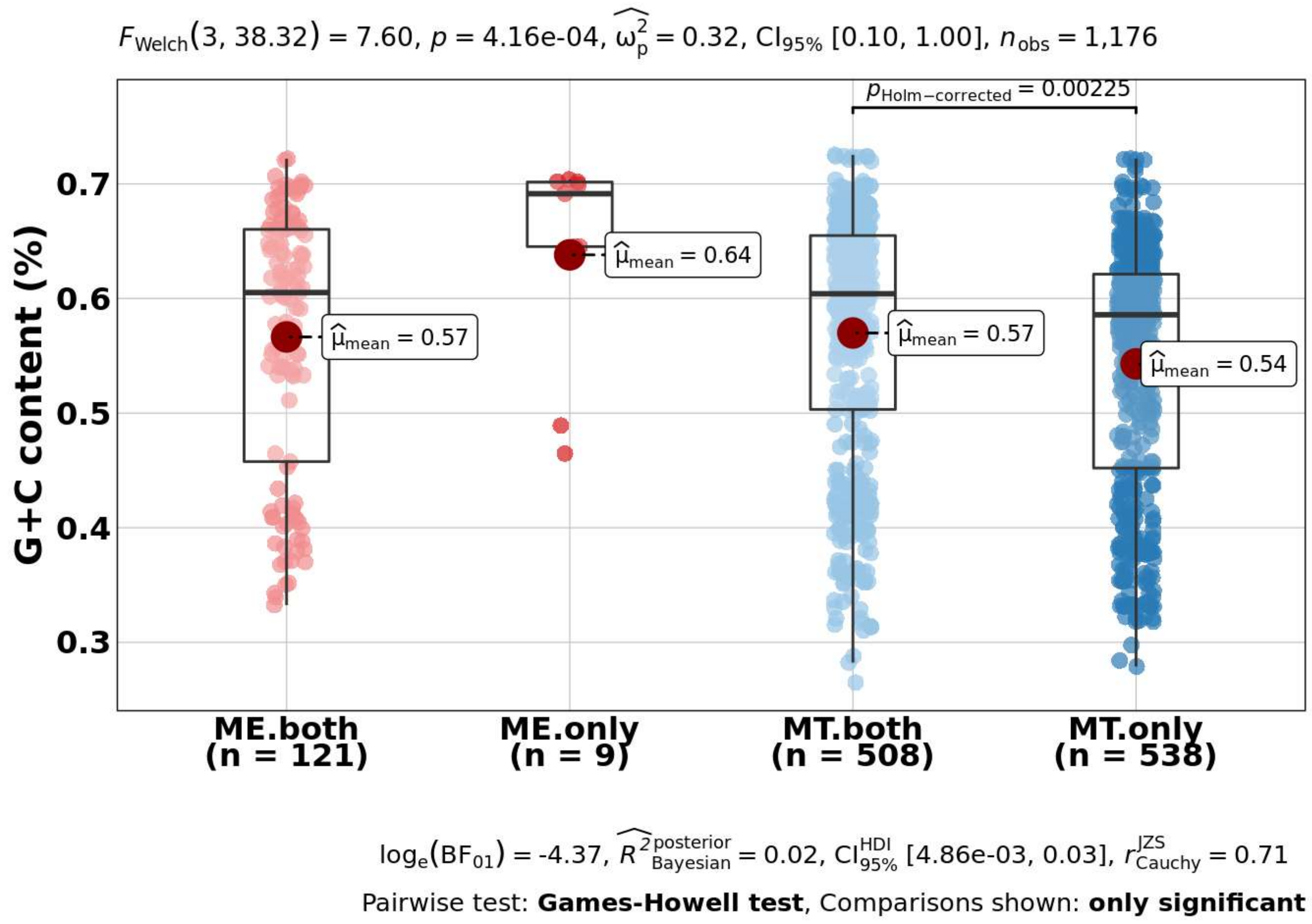


```
p = MAG_info %>% {.[, "Genome size (Mbp)"] = .$GenomeSize / 1e6; .} %>%
  ggbetweenstats.local2("Genome size (Mbp)")
print(p)
```

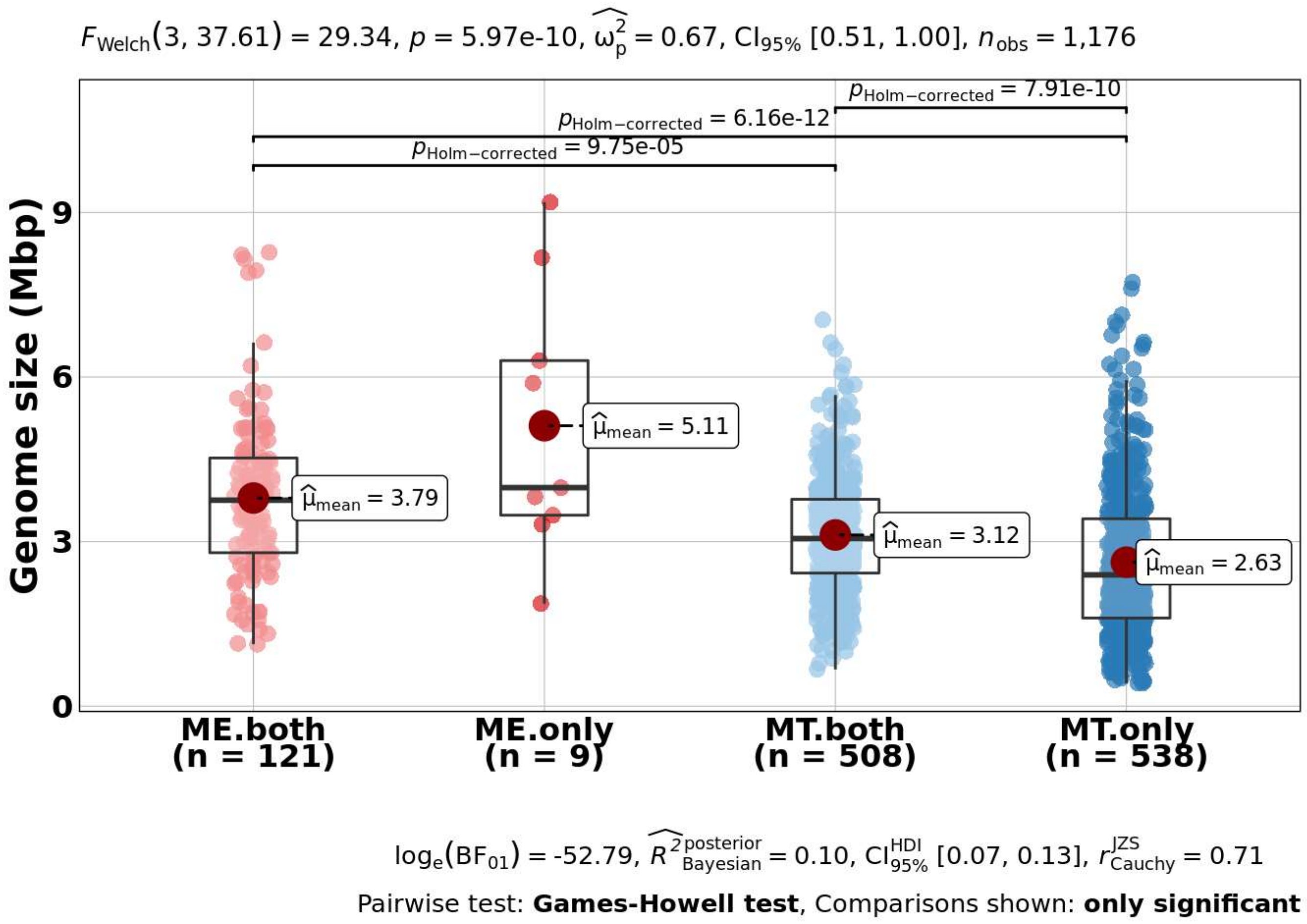


```
p = MAG_info %>% {.[, "Optimal growth temperature (℃)"] = .$GenomeSize / 1e6; .} %>%
  ggbetweenstats.local2("Optimal growth temperature (℃)")
print(p)
```

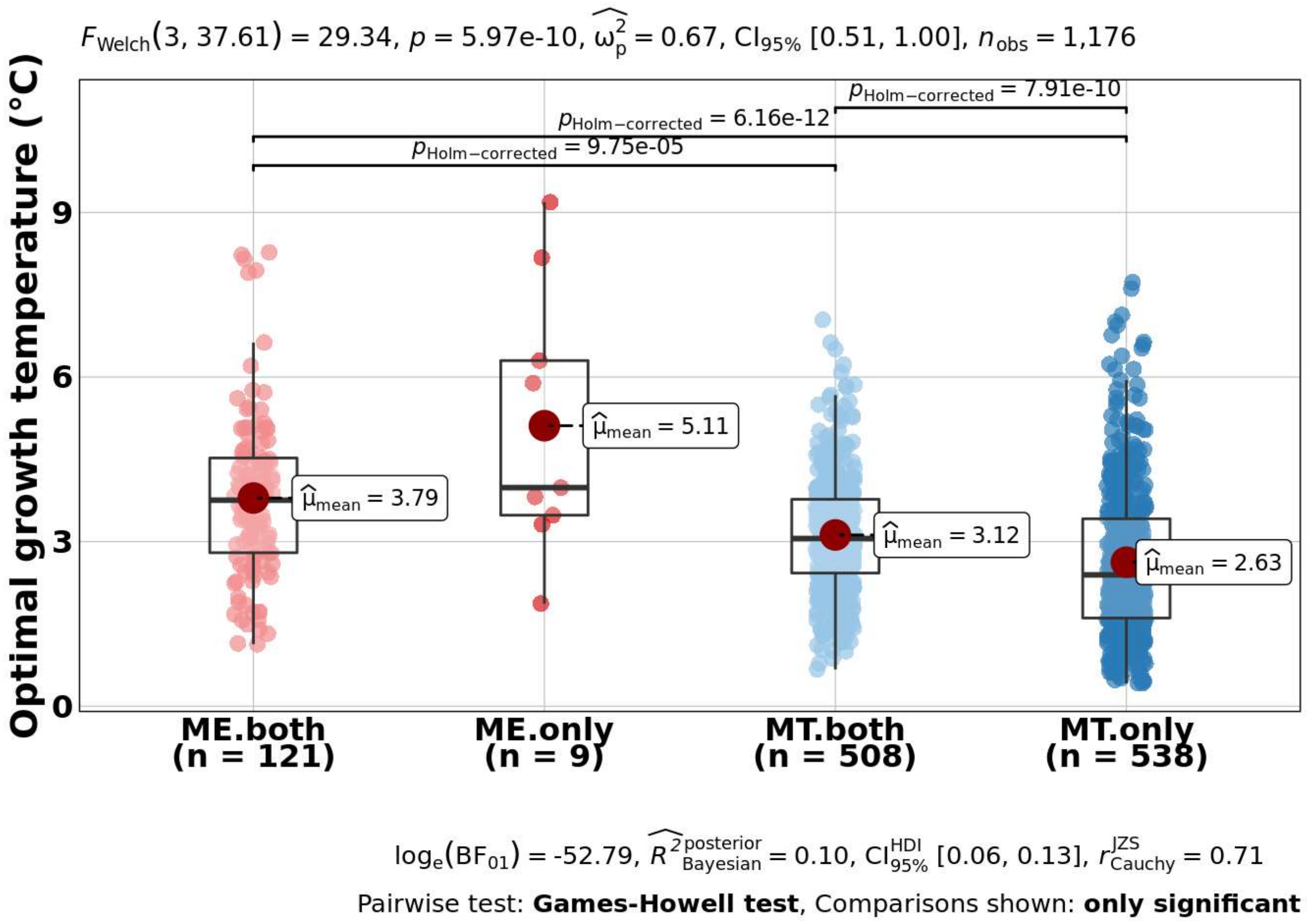

Gene abundance

Percentages of selected metabolic genes across class level in MAGs found in ME only, MT only and both

```
pheatmap(t(ko_class), cluster_cols = F, cluster_rows = F,
         color = c("white", RColorBrewer::brewer.pal(n = 9, name = "OrRd")[c(3, 5, 8)]),
         breaks = c(0, 1e-5, 0.2, 0.5, 1),
         annotation_row = class_env %>% column_to_rownames("class"),
         annotation_colors = ann_colors,
         annotation_col =
           ko_label %>%
           column_to_rownames("label") %>%
           dplyr::select(pathway),
         gaps_row =
           class_env %>% group_by(env) %>% dplyr::summarise(n = n()) %>%
           mutate(n = cumsum(n)) %>% pull(n),
         gaps_col =
           ko_label %>% group_by(pathway) %>% dplyr::summarise(n = n()) %>%
           mutate(n = cumsum(n)) %>% pull(n),
         width = 15, height = 11)
```

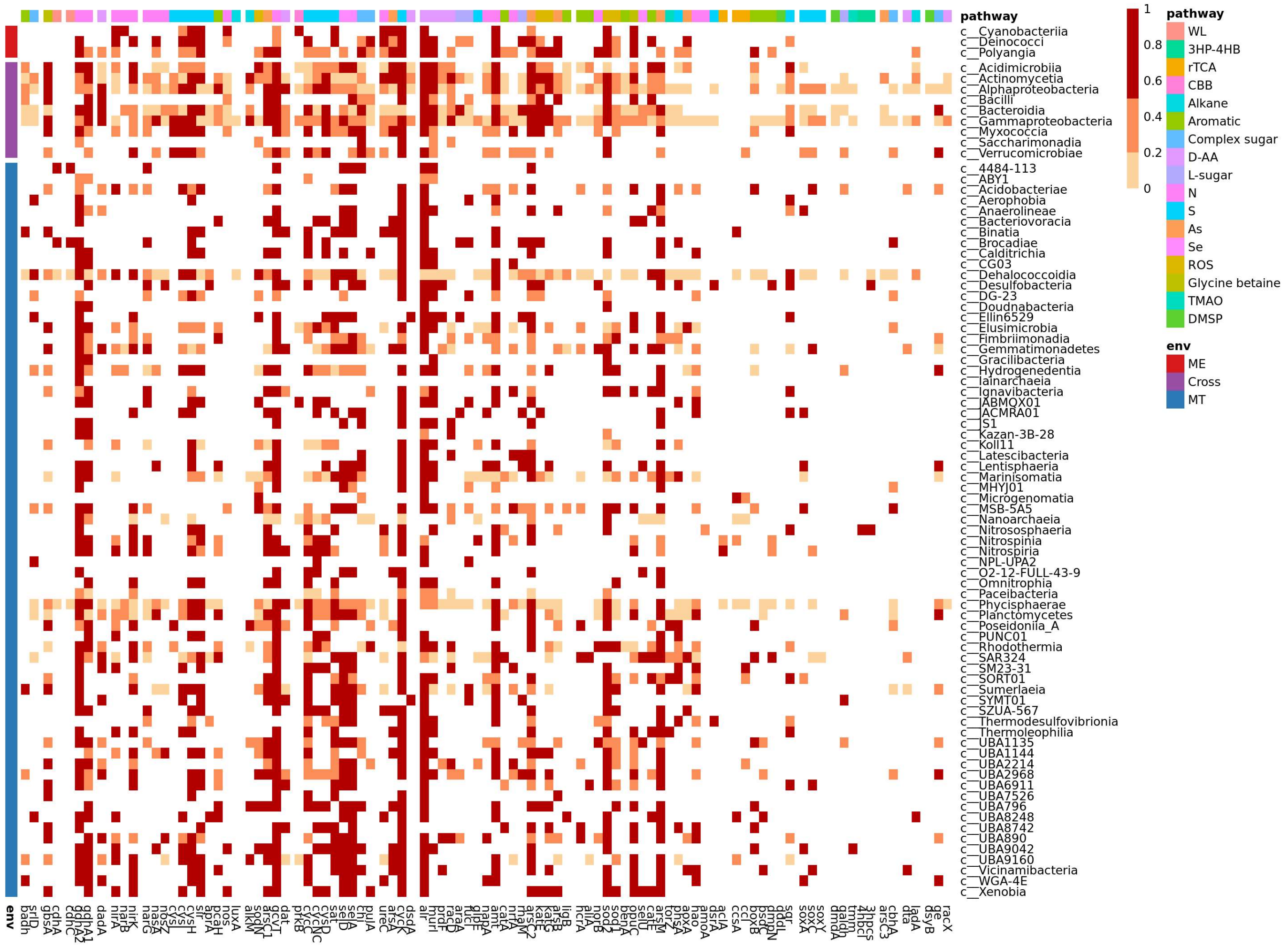

Abundancy of representative metabolic genes in samples from ME and MT

```
tmp.key.signif =
  tpm.key %>%
  split(.$label) %>%
  lapply(function(x) c(
    "p.value" = wilcox.test(formula("tpm ~ location"), data = x,
                            paired = FALSE)$p.value,
    "tpm.max" = max(x$tpm)
  )) %>%
  bind_rows(.id = "label") %>%
  {
    .$p.value.char =
      as.character(symnum(.$p.value,
                          cutpoints = c(0, 0.001, 0.01, 0.05, 0.1, 1),
                          symbols = c("***", "**", "*", ".", "")))
    .
  } %>%
  merge(ko_label, by = "label")

report__tpm.key <- function(labels) {
  #### add paint                                                          ####
  font_size_1 = 16
  font_size_2 = 14
  axis.ticks.length = 0.1

  p =
    ggplot(data = tpm.key %>% .[.$label %in% labels, ]) +
    #geom_signif(mapping = aes_string(x = "location", y = "tpm"),
    #            map_signif_level = TRUE,
    #            comparisons = list(c("ME", "MT"))) +
    geom_boxplot(mapping = aes_string(x = "label", y = "tpm",
                                      fill = "location")) +
    scale_fill_manual(values = sample_meta_col) +

    facet_grid(formula("~ pathway"),
               scales = "free_x", space = "free_x") +

    geom_label(data =
                 tmp.key.signif %>%
                 .[.$label %in% labels, ] %>%
                 .[.$p.value.char != "", ],
               mapping = aes_string(x = "label", y = "tpm.max + max(tpm.max) * 0.05",
                                    label = "p.value.char"),
               label.padding = unit(0.05, "lines"), label.size = 0,
               fill = "#7f7f7f3f") +
    labs(x = "")

  p1 =
    p +
    theme(
      panel.grid.major = element_line(color = 'gray', size = 0.2),
      panel.grid.minor = element_blank(),
      panel.background = element_blank(),
      panel.border = element_blank(),
    ) +
    theme(
```

```
      axis.line = element_line(colour = "black"),
      axis.text = element_text(size = font_size_2, colour = "black", face = "bold"),
      axis.title = element_text(size = font_size_1, face = "bold", colour = "black"),
      axis.ticks.length = unit(axis.ticks.length, 'cm'),

      axis.text.x = element_text(angle = 45, vjust = 1, hjust = 1, face = "plain")
    ) +
    theme(
      legend.title = element_text(size = font_size_2, face = "bold"),
      legend.text = element_text(size = font_size_2, face = "bold")
      #legend.position = "bottom"
    ) +
    theme(text = element_text(family = "Arial",
                              size = font_size_1,
                              hjust = 0.5,
                              lineheight = 0.5)) +
    theme(plot.title = element_text(hjust = 0.5)) +
    theme(legend.key = element_rect(fill = "gray")) +
    theme(strip.background = element_blank(),
          strip.placement = "outside",
          strip.text.x = element_blank())

  p1
}

print(report__tpm.key(ko_label$label[1:40]))
```

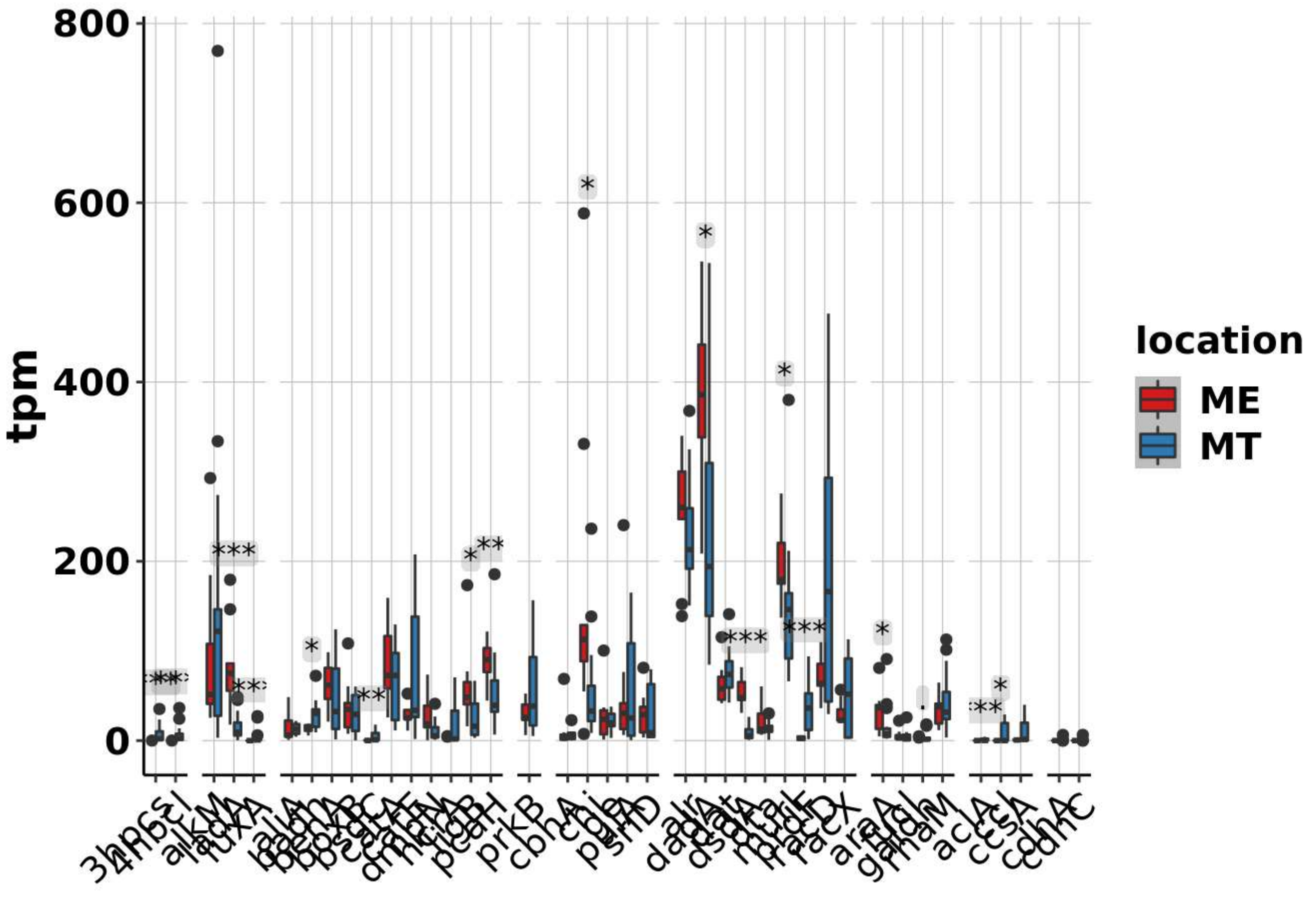


```
print(report__tpm.key(ko_label$label[41:74]))
```

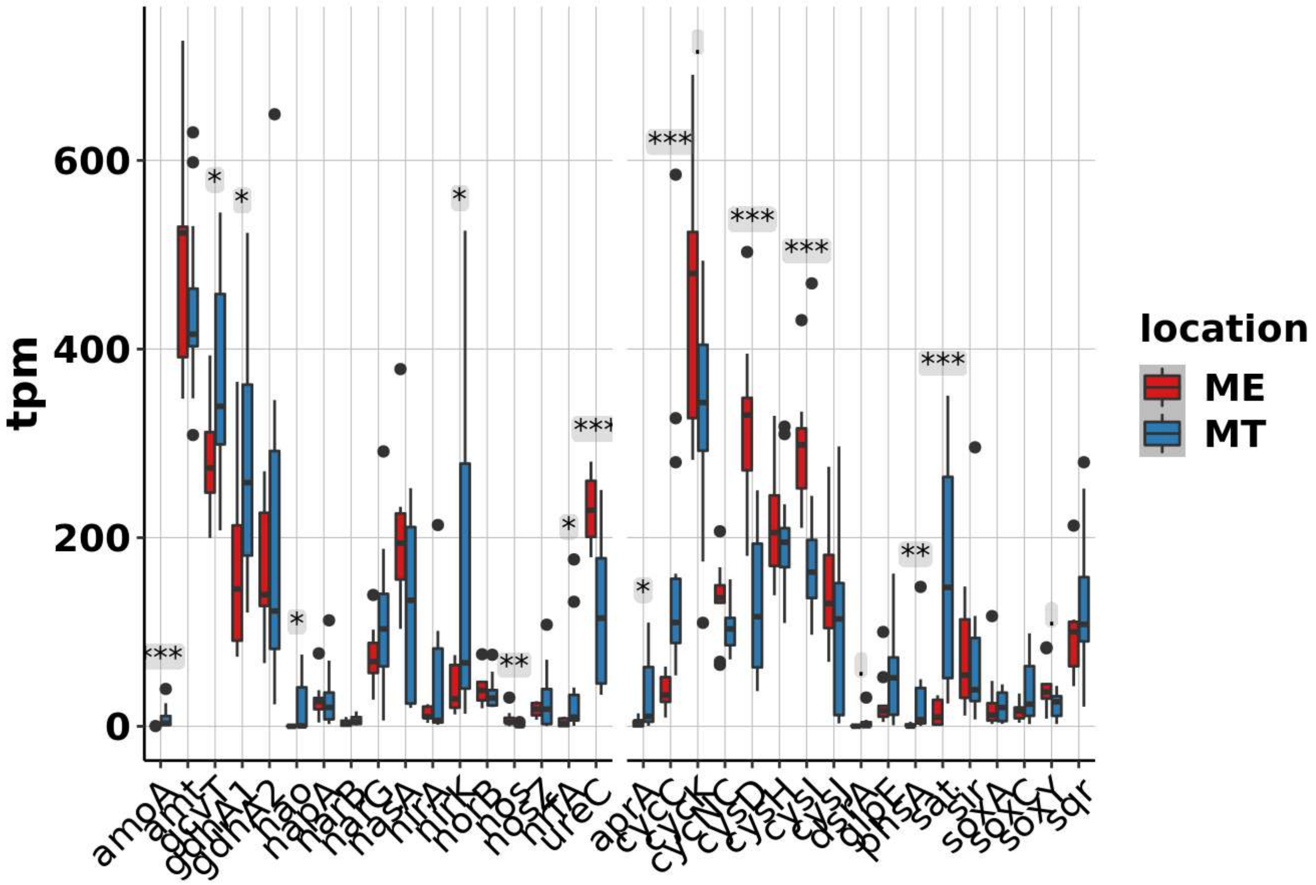


```
print(report__tpm.key(ko_label$label[75:96]))
```

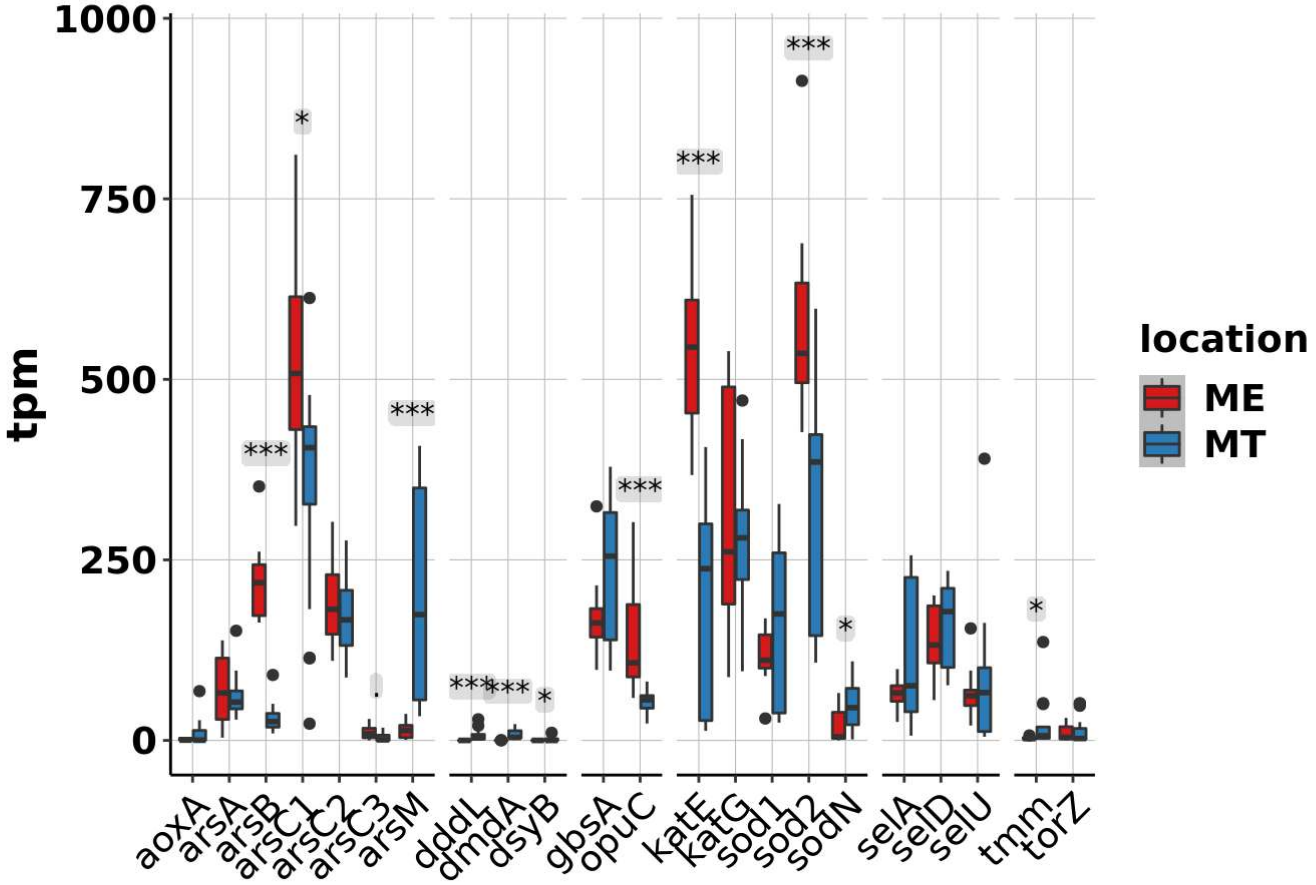

Cooccurrence of different metabolic genes

```
library(igraph)
library(dendextend)
```

```
## Registered S3 method overwritten by 'dendextend':
##   method     from
##   rev.hclust vegan
```

```
##
## ---------------------
## Welcome to dendextend version 1.15.2
## Type citation('dendextend') for how to cite the package.
##
## Type browseVignettes(package = 'dendextend') for the package vignette.
## The github page is: https://github.com/talgalili/dendextend/
##
## Suggestions and bug-reports can be submitted at: https://github.com/talgalili/dendextend/iss
ues
## You may ask questions at stackoverflow, use the r and dendextend tags:
##   https://stackoverflow.com/questions/tagged/dendextend
##
##  To suppress this message use:  suppressPackageStartupMessages(library(dendextend))
## ---------------------
```

```
##
## Attaching package: 'dendextend'
```

```
## The following object is masked from 'package:VennDiagram':
##
##     rotate
```

```
## The following object is masked from 'package:ggpubr':
##
##     rotate
```

```
## The following objects are masked from 'package:ape':
##
##     ladderize, rotate
```

```
## The following object is masked from 'package:permute':
##
##     shuffle
```

```
## The following object is masked from 'package:data.table':
##
##     set
```

```
## The following object is masked from 'package:stats':
##
##     cutree
```

```
ko_jaccard <- function(loc, threshold) {
  m <-
    genomeko %>%
    .[as.character(Wtdb$genome), as.character(ko_label$KO)] %>%
    data.frame(
      .,
      genome = Wtdb$genome,
      location = grepl("^1", Wtdb$genome) %>% ifelse("ME", "MT")
    ) %>%
    as_tibble() %>%
    dplyr::filter(location == loc) %>%
    column_to_rownames("genome") %>%
    dplyr::select(starts_with("K")) %>%
    t()

  m <- m[ko_label$KO, ]
  index <- rowSums(m) > 0
  m.d <- vegdist(m[index, ], method = "jaccard", binary = TRUE)

  dend <- m.d %>%
    hclust(method = "complete") %>%
    as.dendrogram() %>%
    dendextend::set("labels", paste(ko_label$pathway[index], ko_label$label[index], sep = "-"))

  g <- graph_from_adjacency_matrix(1 - as.matrix(m.d), weighted = T,
                                   mode = "undirected", diag = FALSE)

  g.clean <- delete.edges(
    g,
    E(g)[E(g)$weight < threshold]
  )

  g.clean <- delete.vertices(
    g.clean,
    V(g.clean)[degree(g.clean) == 0]
  )

  plot(g.clean,
      vertex.label = with(ko_label, label[match(V(g.clean)$name, KO)]),
      edge.width = E(g.clean)$weight,
      vertex.color =
        paletteer::paletteer_dynamic("cartography::pastel.pal", 17)[
          with(ko_label, pathway[match(V(g.clean)$name, KO)])]
  )
  return(list(dend = dend, g = g, g.clean = g.clean))
}

ko_ME <- ko_jaccard("ME", 0.3)
```

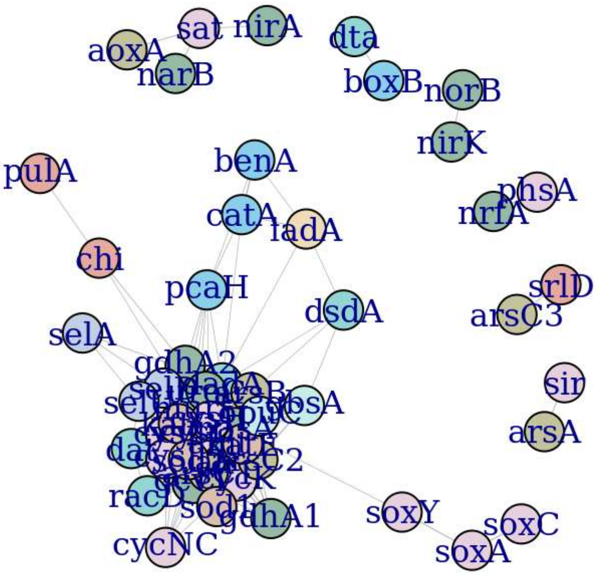


```
ko_MT <- ko_jaccard("MT", 0.3)
```

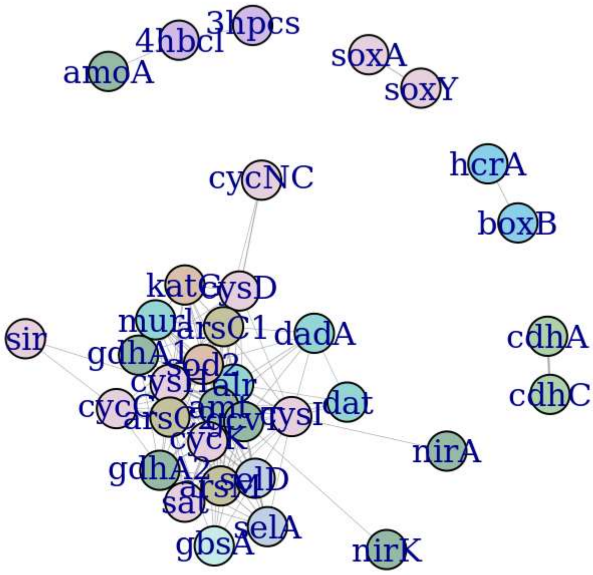

- the cooccurrence of different metabolic genes was quite different across prokaryotic genomes in ME and MT

```
dends <- dendlist(ko_MT$dend, ko_ME$dend)
tanglegram(
  dends,
  main_left = "ME", main_right = "MT",
  common_subtrees_color_branches = TRUE,
  lwd = 2, edge.lwd = 1.5,
  columns_width = c(5,1,5),
  margin_inner = 9)
```

```
## Warning in intersect_trees(dend1, dend2, warn = TRUE): The labels in both tree
## had different values - trees were pruned.
```

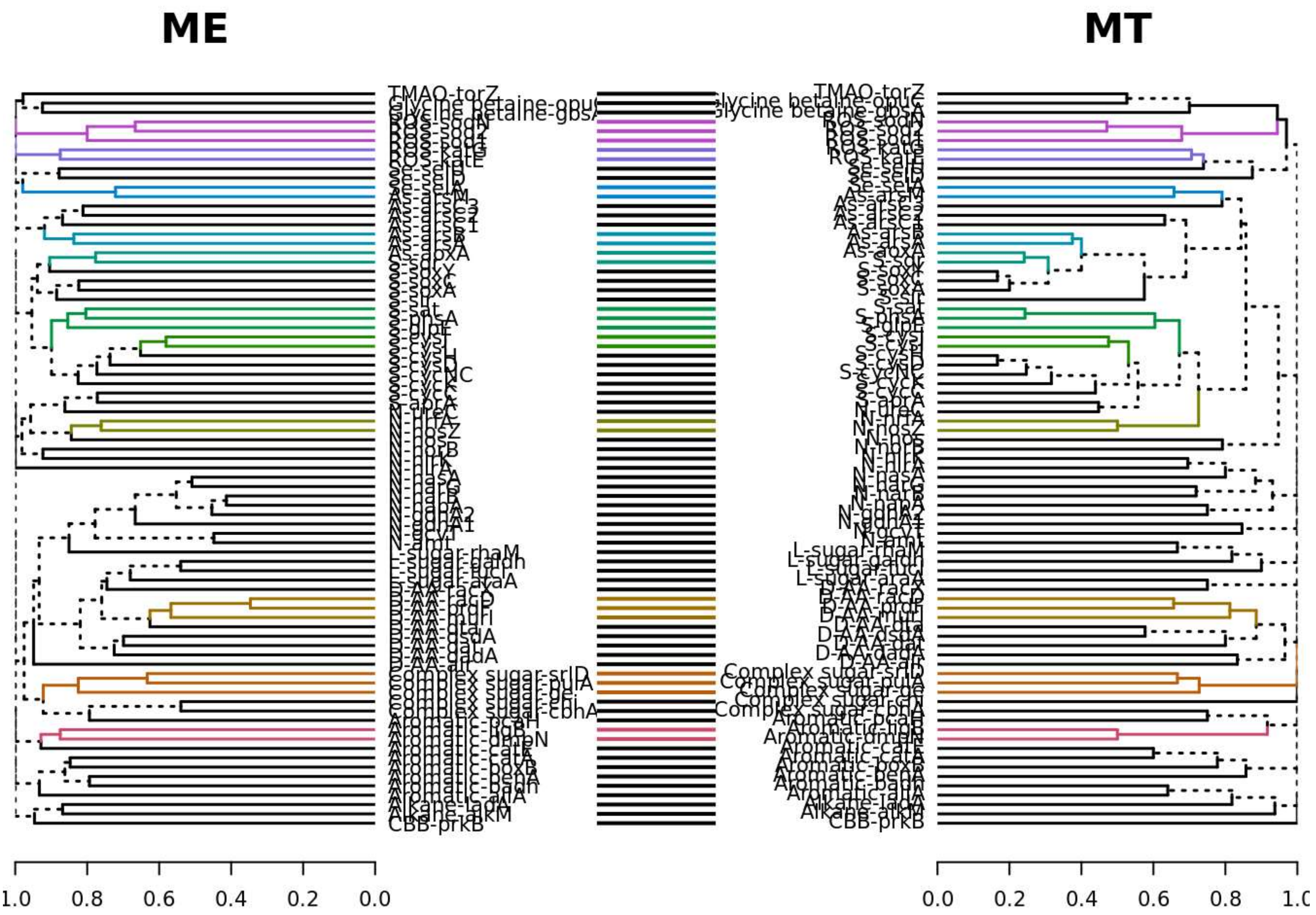

Supplement: Supplementary file 5 — Additional file 4. Workflow. [file 40168_2022_1403_MOESM4_ESM.pdf]
